# Haemoglobin concentration thresholds that discriminate functional outcomes among children aged 6–30 months in eight countries: a pooled analysis of individual participant data

**DOI:** 10.1136/bmjgh-2024-015866

**Published:** 2026-04-28

**Authors:** Elizabeth L Prado, Leila M Larson, Charles D Arnold, K Ryan Wessells, Anuraj H Shankar, Seth Adu-Afarwuah, Benjamin F Arnold, Per Ashorn, Ulla Ashorn, Elodie Becquey, Kenneth H Brown, Jaya Chandna, Bernard Chasekwa, Yue-Mei Fan, Emanuela Galasso, Sonja Y Hess, Lieven Huybregts, Kaniz Jannat, Elizabeth Yakes Jimenez, Anna Lartey, Agnes Le Port, Jef L Leroy, Stephen P Luby, Kenneth Maleta, Andrew Matchado, Susana L Matias, Malay Kanti Mridha, Kuda Mutasa, Clair Null, Harriet Okronipa, Jean Bosco Ouédraogo, Amy Pickering, John Phuka, Anna Pulakka, Lisy Ratsifandrihamanana, Christine P Stewart, Fahmida Tofail, Ann M Weber, Kathryn G Dewey

**Affiliations:** 1 Institute for Global Nutrition & Department of Nutrition, University of California Davis, Davis, California, USA; 2 Department of Health Promotion, Education, and Behavior, University of South Carolina, Columbia, South Carolina, USA; 3 Centre for Tropical Medicine and Global Health, Nuffield Department of Medicine, University of Oxford, Oxford, UK; 4 Oxford Clinical Research Unit Indonesia, Central Jakarta, Indonesia; 5 Department of Nutrition and Food Science, University of Ghana, Accra, Ghana; 6 Francis I. Proctor Foundation, University of California San Francisco, San Francisco, California, USA; 7 Center for Child, Adolescent and Maternal Health Research, Tampere University Faculty of Medicine and Health Technology, Tampere, Finland; 8 Department of Paediatrics, Tampere University Hospital, Tampere, Finland; 9 Nutrition, Diets, and Health Unit, International Food Policy Research Institute, Washington, District of Columbia, USA; 10 Helen Keller International, New York, New York, USA; 11 Department of Infectious Disease Epidemiology, London School of Hygiene & Tropical Medicine, London, UK; 12 Zvitambo Institute for Maternal and Child Health Research, Harare, Zimbabwe; 13 Development Research Group, World Bank, Washington, District of Columbia, USA; 14 School of Health Sciences, Western Sydney University, Penrith, New South Wales, Australia; 15 Departments of Pediatrics and Internal Medicine and College of Population Health, The University of New Mexico Health Sciences Center, Albuquerque, New Mexico, USA; 16 Montpellier Interdisciplinary Center on Sustainable Agri food systems, French National Research Institute for Sustainable Development, Marseille, France; 17 Division of Infectious Diseases and Geographic Medicine, Stanford University, Stanford, California, USA; 18 Department of Public Health, School of Public Health and Family Medicine, University of Malawi College of Medicine, Blantyre, Malawi; 19 Department of Public Health, School of Global and Public Health, Kamuzu University of Health Sciences, Blantyre, Malawi; 20 Department of Medical Biology and Nutrition, University of California Berkeley, Berkeley, California, USA; 21 Center for Non-Communicable Diseases and Nutrition, BRAC University James P Grant School of Public Health, Dhaka, Bangladesh; 22 Mathematica Inc, Washington, Washington, USA; 23 Department of Nutritional Sciences, Oklahoma State University, Stillwater, Oklahoma, USA; 24 Health Sciences Research Institute, Bobo-Dioulasso, Burkina Faso; 25 Tufts University School of Engineering, Medford, Massachusetts, USA; 26 Research Unit of Population Health, University of Oulu, Oulu, Finland; 27 Centre Médico-Educatif "Les Orchidées Blanches”, Antananarivo, Madagascar; 28 Nutrition and Clinical Services Division, International Centre for Diarrhoeal Disease Research, Dhaka, Bangladesh; 29 Department of Biostatistics, Epidemiology and Environmental Health, University of Nevada Reno School of Public Health, Reno, Nevada, USA

**Keywords:** Anaemia, Child health

## Abstract

**Introduction:**

The WHO cut-offs to define anaemia among children (haemoglobin, Hb <105 g/L in 6–23 months and <110 g/L in 24–59 months) are based on the distribution of Hb concentrations in a healthy population. Our objective was to identify Hb values that best discriminate functional outcomes among children aged 6–30 months.

**Methods:**

We used previously compiled datasets from an individual participant data meta-analysis of effects of small quantity lipid-based nutrient supplements. Participants were eligible for this pooled analysis if they had Hb measured at 6–30 months and data on at least one functional outcome of interest, which included physical activity and sleep patterns, and language, socioemotional and motor development. We stratified the datasets by child age in 3-month intervals and analysed associations of Hb with both concurrent and subsequently measured (longitudinal) outcomes. If Hb significantly discriminated the 25th, 50th or 75th percentile of the outcome based on the pooled area under the receiver operating characteristic curve (AUC), we identified the Hb value with the highest concordance probability as the best discriminatory threshold.

**Results:**

11 datasets from 8 countries including 27 626 children were analysed. Hb significantly discriminated 21 of 47 concurrent and 11 of 32 longitudinal Hb-outcome associations. Best Hb discriminatory thresholds ranged from 102 to 111 g/L for concurrent physical activity outcomes, 103–116 g/L for concurrent developmental outcomes, and 109–117 g/L for longitudinal developmental outcomes. The I^2^ for the pooled AUC analysis indicated generally low to moderate heterogeneity across studies.

**Conclusions:**

The current WHO cut-offs to define anaemia among children are in the middle to upper range of Hb values that best discriminate concurrent physical activity and development, and are in the lower range of values that best discriminate subsequent child development. Along with other types of evidence, this study provides additional evidence to inform Hb cut-offs to define anaemia among young children.

**WHAT IS ALREADY KNOWN ON THIS TOPIC**

- Until recently, the WHO recommended haemoglobin (Hb) <110 g/L to define anaemia for children aged 6–59 months. The selection of this cut-off in 1968 was based on a study of 129 children aged 6–18 months deemed to have normal Hb concentrations, none of which were lower than 112 g/L.
- In 2024, updated WHO guidelines, using a statistical approach to determine an abnormally low Hb concentration, recommended new cut-offs to define anaemia as Hb <105 g/L in children aged 6–23 months and <110 g/L in children aged 24–59 months.
- The WHO guideline indicated that a preferred approach to establish Hb cut-offs to define anaemia would be based on Hb values that correspond with declining functional outcomes, such as physical activity and motor development, but evidence for this approach was insufficient.

**WHAT THIS STUDY ADDS**

- This study identified Hb concentrations that best discriminate functional outcomes (physical activity, sleep and child development) among children aged 6–30 months by a pooled analysis of individual participant data from 11 datasets from 8 low-income and middle-income countries (LMICs), including 27 626 children.
- Best Hb discriminatory thresholds ranged from 102 to 111 g/L for concurrent physical activity outcomes (measured at age 18 months), 103 to 110 g/L for concurrent developmental outcomes (measured at 12–24 months, with one 27-month best Hb threshold at 116 g/L), and 109 to 117 g/L for longitudinal developmental outcomes.
- Best Hb thresholds tended to be higher for longitudinal associations compared with concurrent associations and tended to be higher for older compared with younger children.

**HOW THIS STUDY MIGHT AFFECT RESEARCH, PRACTICE OR POLICY**

- The current WHO cut-offs to define anaemia among children aged 6–23 months (Hb <105 g/L) and 24–59 months (Hb <110 g/L) are in the middle to upper range of the Hb values that best discriminate concurrent physical activity and development, and are in the lower range of values that best discriminate subsequent child development.
- This evidence should be considered along with other evidence, such as studies examining the distribution of Hb in healthy populations, to determine the cut-off to define anaemia for a given age group.

## Introduction

 In 2019, an estimated 40% of children aged 6–59 months globally were anaemic, defined as haemoglobin concentration (Hb) <110 g/L.^1^ One way to inform a diagnostic cut-off value to define anaemia is to use a statistical approach to determine an abnormally low Hb concentration, such as the fifth percentile of a healthy population. Another type of evidence that can be considered along with the statistical approach is to identify Hb values that best discriminate functional outcomes. Anaemia is a condition in which insufficient red blood cells result in inadequate oxygen delivery throughout the body. Some common causes of anaemia in low-income and middle-income countries (LMICs) are iron deficiency, inherited red blood cell disorders, malaria, other infections and inflammation.^2^ Anaemia causes fatigue,^3^ thus low Hb may increase time spent in sedentary behaviour and sleeping, decrease time spent in moderate to vigorous physical activity (MVPA), and reduce exploration of the environment and interaction with caregivers, which could affect motor, cognitive and socioemotional development.^4^ The fatigue and poor developmental outcomes associated with anaemia^3 5^ may be caused by lower oxygen delivery to cells or altered energy metabolism^6^ or may be caused independently by the underlying causes of anaemia, such as iron deficiency or malaria.^7^,^10^ It is also possible that the association of anaemia with functional outcomes could be confounded by poverty and other environmental factors.^2^

Although the WHO recommends Hb <105 g/L in children 6–23 months and <110 g/L in children 24–59 months to define anaemia, it is not known whether these are the best Hb thresholds to discriminate functional consequences of low Hb during early childhood.^11^ The 2024 WHO guidelines were based on statistical cut-offs (ie, fifth percentile) of Hb concentrations in apparently healthy populations using data from ethnically diverse healthy populations, general population databases and systematic reviews.^12^ The guidelines indicated a preference for establishing Hb cut-offs to define anaemia using an approach that is based on outcomes related to clinical symptoms and functional impairment, but evidence for this approach was insufficient to be included in the current guidelines.

The aim of the present study was to identify Hb concentration values that best discriminate the functional outcomes of physical activity, sleep and child development among children aged 6–30 months. We concluded from a previous systematic review and meta-analysis of published studies that evidence from the existing literature was inadequate to identify Hb values that best discriminate functional outcomes.^13^ In this study, we address this evidence gap by using a pooled analysis of individual participant data from 11 datasets from 8 LMICs including 27 626 children. Our first objective was to determine which Hb values best discriminate higher versus lower functional concurrent and longitudinal outcomes across contexts. Concurrent analyses capture deficits in outcomes (such as physical activity or motor development) at the time that the child experiences anaemia, while longitudinal analyses capture faltering in outcomes in the subsequent 6 to 18 months. Our second objective was to describe the shape of the association of Hb with each outcome.

## Methods

The protocol for this pooled analysis was posted to Open Science Framework prior to analysis (https://osf.io/5qrjh/).

### Inclusion and exclusion criteria for this pooled analysis

For this study, we used a convenience sample of datasets that were included in an individual participant data meta-analysis of effects of small quantity lipid-based nutrient supplements (SQ-LNS) on child outcomes; study- and individual-level eligibility criteria were previously reported.^14^ Individual participants were eligible for the present analysis if they had at least one Hb concentration measurement at any age between 6 and 30 months and data on at least one functional outcome of interest measured either concurrently or at a subsequent age (longitudinal) ie, we used a complete case analysis.

### Data collection

From the previously compiled dataset, variables used for this pooled analysis were (1) Hb concentration measured at any age from 6 to 30 months, (2) functional outcomes of interest, as described below and (3) variables included as covariates and potential effect modifiers, also described below.

### Specification of exposures

The exposure of interest was child Hb concentration, as measured in each trial. All trials assessed Hb using a Hemocue System (Hemocue AB, Angelholm, Sweden); five studies used a venous sample and six studies used a capillary sample (table 1).

**Table 1:** In each trial, haemoglobin (Hb) assessment and developmental assessment methods used and sample characteristics*

Trial, country, and reference(s)	Hb sample and method	Language	Motor	Socioemotional	Child mean (SD) age at latest biomarker assessment: months	Mean (SD) Hb at latest biomarker assessment: g/L	Anaemia (Hb <110 g/L) at latest biomarker assessment: %	Maternal education, completed primary: %
RDNS Bangladesh Dewey, 2017^33^; Matias, 2018^34^	Capillary; Hemocue Hb 301	CDI	DMC	DMC	18.1 (0.09)	115.2 (13.0)	31.9	73.6
WASH-B-Bangladesh Luby, 2018^35^ Stewart, 2019^36^	Venous; Hemocue Hb 301	CDI	EASQ	EASQ	27.8 (1.86)	120.1 (8.9)	11.4	71.2
iLiNS-Zinc Burkina Faso Hess, 2015^37^; Abbeddou, 2017^38^	Capillary; Hemocue Hb 201+	DMC	DMC	DMC	18.3 (0.40)	95.1 (15.8)	81.2	3.7
Ghana Adu-Afarwuah, 2007^39^	Venous; HemoCue B-Haemoglobin	–	–	–	12.0 (0.15)	111.0 (14.1)	42.1	86.7
iLiNS-DYAD-Ghana Adu-Afarwuah, 2016^40^; Adu-Afarwuah, 2019^41^	Venous; Hemocue Hb 301	CDI	KDI	PSED	18.1 (0.58)	112.3 (10.4)	43.0	78.8
WASH-B-Kenya Null, 2018^42^; Stewart, 2019^36^	Venous; Hemocue Hb 301 (altitude adjusted)^43^	EASQ	EASQ	EASQ	22.4 (1.83)	112.0 (12.9)	39.3	47.6
MAHAY Madagascar Galasso, 2019^44^	Capillary; HemoCue Hb 301	ASQ:I	ASQ:I	ASQ:I	25.8 (3.85)	111.3 (12.2)	41.0	23.5
iLiNS-DYAD-M Malawi Ashorn, 2015^45 46^	Capillary; HemoCue Hb 201+	CDI	KDI	PSED	18.0 (0.11)	108.7 (15.3)	49.2	16.6
iLiNS-DOSE Malawi Maleta, 2015^47^	Capillary; HemoCue Hb 201+	CDI	KDI	PSED	18.1 (0.31)	102.9 (15.4)	66.5	23.5
PROMIS-Mali Huybregts, 2019^48^	Capillary; Haemocue Hb 201+	DMC	DMC	DMC	14.8 (5.13)	99.6 (13.4)	76.2	10.7
SHINE Zimbabwe Humphrey, 2019^49^; Prendergast 2019^50^	Venous; Haemocue Hb 301 (altitude adjusted)^43^	CDI	MDAT	MDAT	18.1 (0.73)	116.4 (11.7)	29.0	96.1

All developmental assessments were conducted when the children were ages 12–30 months. In this age range, all of these tools assess similar developmental skills and many items overlap between the tools.

* Values are mean (SD) or %; Adu-Afarwuah (2007) measured individual milestones only (walking at 12 months); Physical activity was measured in iLiNS-DYAD-M and iLiNS-DOSE; Sleep was measured in iLiNS-DYAD-M and iLiNS-DYAD-G.

ASQ:I, Ages and Stages Questionnaire Inventory; CDI, MacArthur-Bates Communicative Development Inventory; DMC, Developmental Milestones Checklist; EASQ, Extended Ages and Stages Questionnaire; iLiNS-DOSE, Prevention of Linear Growth Failure in Infants and Young Children With Lipid-based Nutrient Supplements Trial; iLiNS-DYAD-M, International LIpid-based Nutrient Supplement trial of mother-child dyads in Malawi; KDI, Kilifi Developmental Inventory; MDAT, Malawi Developmental Assessment Tool; PROMIS, Innovative Approaches for the Prevention of Childhood Malnutrition; PSED, Profile of Social and Emotional Development; RDNS, Rang-Din Nutrition Study; SHINE, Sanitation Hygiene Infant Nutrition Efficacy Trial; WASH-B, Water Quality, Sanitation, and Hand Washing Benefits Study.

### Specification of functional outcomes

The following outcomes were prespecified in the analysis plan. For further details, see online supplemental methods.

#### Physical activity

In two trials, 18-month-old children wore Actigraph GT3X+accelerometers for 7 days.^15 16^ Outcomes were (1) the mean vector magnitude (MVM) counts/min during waking hours, (2) percentage of waking hours spent in MVPA and (3) percentage of waking hours spent in sedentary behaviour. All physical activity outcomes were standardised in the same way as the developmental scores (standardised residuals by child age and sex; see below).

#### Sleep

In two trials, sleep was characterised as a (1) night time sleep duration index and (2) day time nap index at age 6, 12 and 18 months. Higher values indicated longer sleep/naps. Sleep outcomes were standardised in the same way as the developmental scores.

#### Developmental outcomes

Language, socioemotional and gross and fine motor development were measured in 10 trials and were defined in the same way as the previous IPD meta-analysis, based on the same rationale (see Online supplemental methods).^17^ Raw scores were calculated according to the established method for the tool used in each study (table 1) then standardised within each study by regressing the raw developmental score on child age and sex and calculating the standardised residuals. Using this approach, the score represents deviations from the predicted score for a given child’s age and sex in units of SD. We also analysed whether the child was walking alone at age 12 months using reports or observations of the child’s ability collected within the range of 11.0–13.0 months.

### Specification of ages of interest

We stratified the datasets by child age and analysed all ages (6, 9, 12, 15, 18, 21, 24, 27 and 30±1.5 months) for which the minimum adequate sample size was available. For each potential analysis (ie, the combination of Hb at a given age and a given outcome), minimum was defined as: (1) measurements available from ≥30 children for a study to contribute, (2) at least two studies had to contribute data and (3) the total sample size for the analysis had to include at least 352 children. The latter is the minimum sample size to detect a meaningful 2-group mean difference of 0.3 SD^18^,^21^ with 80% power and alpha of 0.05. Adequate sample size in the main analysis was considered a sufficient criterion to conduct the sensitivity and stratified analyses (for further details, see online supplemental methods).

At each age, we examined (1) concurrent discriminatory thresholds, that is, associations of child Hb with concurrently measured functional outcomes and (2) longitudinal discriminatory thresholds, that is, associations of child Hb with subsequently measured functional outcomes. For the longitudinal analyses, we used walking at 12 months for Hb measured at any age before 12 months and we used the other outcomes at 18 or 24 months, whichever was available, for Hb measured at any age before 18 or 24 months. If both were available, we used 18 months for Hb measured at any age before 18 months.

### Synthesis methods and exploration of variation in effects

**Figure 1:**
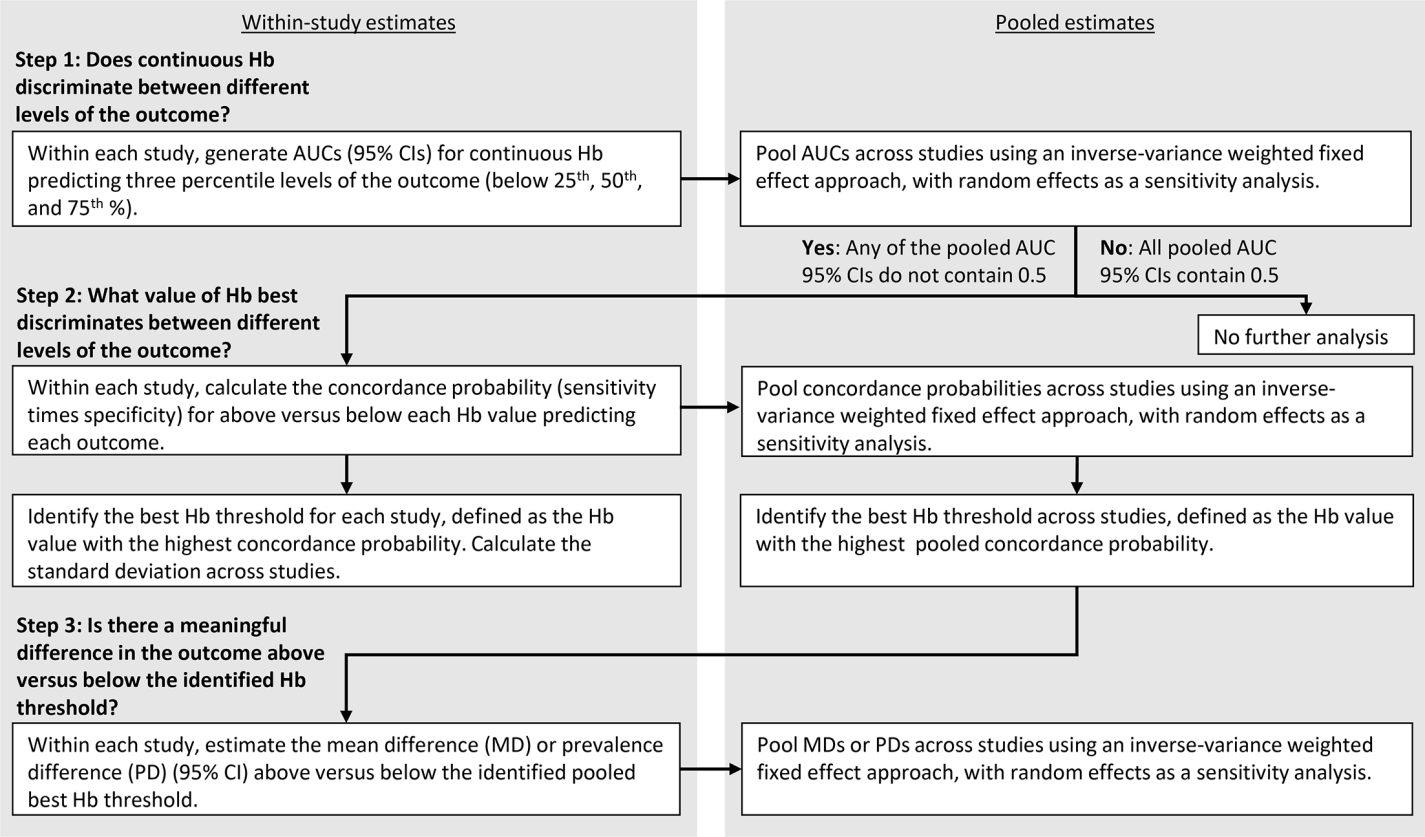
Analysis steps for objective 1, to determine which Hb values best discriminate higher versus lower functional outcomes across contexts. AUC, area under the curve; Hb, haemoglobin.

Analyses were conducted in R V.4.1.1 (Vienna, Austria; R Core Team) and Stata SE V.15-16 (StataCorp). For the first objective, to determine which Hb values best discriminated higher versus lower functional outcomes, the first step was to determine whether Hb was able to discriminate between different levels of the outcome (figure 1). For each continuous outcome within each study, we generated three area under the receiver operating characteristic curves (AUCs) for continuous Hb predicting three binary outcomes defined respectively as <25^th^, < 50^th^ and < 75^th^ percentile of the continuous outcome.^22^ These cut-points were used for several reasons (detailed in the online supplemental methods), but primarily because internal standardisation allowed harmonisation of the calculation of developmental assessment scores across trials given that different tools were used in the different trials and there is no external reference to standardise scores. Then, for each binary outcome, the study-level estimates (AUCs and 95% CIs) were pooled using an inverse-variance weighted fixed effect approach, with random effects models as a sensitivity analysis. We used a fixed effect approach as our primary approach, given that we expected the studied relationships to be biological and therefore similar across contexts. I^2^ statistics were used to describe the percentage of the variability in effect estimates that is due to heterogeneity.

Second, if any of the three pooled AUCs indicated that continuous Hb had any ability to discriminate between different levels of the functional outcome (ie, the pooled AUC 95% CI did not contain 0.5), then we determined which value of Hb best discriminated between the different levels of the outcome, defined as the Hb value with the highest concordance probability (sensitivity multiplied by specificity).^23^ Within each study, we calculated the concordance probability for the dichotomous variable above versus below each Hb value predicting the outcome. To measure variance across study samples, we calculated the SD of the best Hb thresholds across studies. The study level estimates (concordance probabilities) were then pooled using an inverse-variance weighted fixed effect approach, with random effects models as a sensitivity analysis. The best Hb threshold across studies was defined as the Hb value with the highest pooled concordance probability (figure 1).

Third, we determined whether the thresholds identified in step 2 corresponded to meaningful differences in the outcome between subgroups above versus below the identified best threshold. We used analysis of variance models to estimate the mean difference in the outcome above versus below the best Hb threshold for continuous outcomes and prevalence difference for the binary outcome (walking alone). We pooled the study-level estimates using an inverse-variance weighted fixed effect approach, with random effects as a sensitivity analysis. We considered a mean difference between high and low Hb groups to be meaningful if it was >0.07 SD. This magnitude mean difference is analogous to 1 IQ point on an IQ test, which is typically standardised to a mean of 100 and SD of 15 (1/15=0.07), and represents a meaningful difference at the population level in relation to educational achievement and human capital.^24^

We also conducted this analysis adjusting for intervention arm (SQ-LNS vs control) and any of the following covariates that were available in the given trial dataset: maternal age, maternal education, household asset index, household food insecurity, number of children <5 years in the household, child sex and home environment. All datasets included at least one covariate, thus the datasets used in the unadjusted and adjusted analyses were identical. Heterogeneity was assessed using I^2^ statistics.

For objective 2, we visualised the shape of the association between Hb and each outcome by plotting the pooled outcome mean for each 5 g/L Hb bin. If the bin plot showed an inverse U-shape, we explored whether there was a threshold in the upper range of Hb at which higher Hb was associated with relatively poorer functional outcomes. We grouped the data into two overlapping groups based on consensus visual inspection of the bin plot. One group included the low Hb values and the mid-range of Hb values, while the second group included the high Hb values and the mid-range of Hb values. We repeated all analysis steps described above for Objective 1 in these subgroups separately to determine the Hb values, if any, that best discriminated the upper (vs middle) and lower (vs middle) ranges of the Hb distribution. For details, see online supplemental methods.

### Sensitivity analyses to assess robustness of the results

In addition to the sensitivity analysis using random effects models, we conducted sensitivity analyses stratified by intervention and control group to examine differences in either sub-group compared with the full group. A detailed description of the intervention and control groups is described elsewhere.^14^

### Exploratory stratified analyses

We conducted exploratory stratified analyses by the following variables, if available: (1) child sex (female/male), (2) inflammation-adjusted^25^ iron deficiency (ferritin <12 µg/L or zinc protoporphyrin >70 µmol/mol heme; yes/no), (3) malaria (yes/no), (4) inflammation (C reactive protein >5 mg/L; yes/no), (5) blood sampling site (capillary/venous) and (6) HIV exposure, based on maternal HIV positive (yes/no). For iron deficiency, malaria and inflammation, we used measurements taken concurrently with Hb measurement.

Our analyses did not correct for multiplicity given the limited number of outcomes, all of which were specified a priori and are based on evidence of biological plausibility. Furthermore, our analysis is not assuming a universal null hypothesis that Hb is not associated with any of the functional outcomes of interest.^26^

### Patient and public involvement

Patients and the public were not involved in the design of these analyses because the analyses were not part of the original objectives of the studies and were designed post hoc.

## Results

### Trial and participant characteristics

11 trials and 27 626 children were included in this analysis. Two trials were conducted in Bangladesh and the rest in seven countries in sub-Saharan Africa (table 1). Mean child Hb concentration at latest biomarker assessment (age 12–28 months) in the sample ranged from 95.1 to 120.1 g/L across studies (table 1; online supplemental figure 1). Anaemia prevalence (Hb <110 g/L) ranged from 11.4% to 81.2%, iron deficiency from 11.4% to 43.7%, and malaria prevalence from 1.1% to 18.6% (table 1; online supplemental table 1). Other characteristics are shown in online supplemental table 1. The ages at which Hb and each outcome were measured in each trial are shown in online supplemental table 2.

### Identification of discriminatory thresholds

A total of 79 Hb-outcome combinations met the minimum sample size criteria, 47 concurrent associations and 32 longitudinal (online supplemental table 3). Of these 79, Hb significantly discriminated the outcome (ie, pooled AUC 95% CI did not contain 0.5 for at least one of the three quartile-derived binary outcomes) for 21 concurrent and 11 longitudinal associations in fixed effect models (online supplemental table 4).

We identified discriminatory thresholds for five longitudinal outcomes for Hb measured at age 6 months, ranging from 106 to 113 g/L. At 6 months, Hb >109 g/L was associated with a 5 percentage points higher prevalence of walking alone at 12 months, with a pooled prevalence of 43% walking alone at 12 months among children with 6-month Hb >109 g/L. The prevalence of children walking alone at 12 months in each study is reported in online supplemental table 1. For the 12-month sleep index, 18-month nap index and gross motor score and 24-month socioemotional score, we did not find meaningful differences in outcomes (ie, differences larger than 0.07 SD) in subgroups above versus below the identified 6-month Hb thresholds (online supplemental table 4).

For Hb measured at 9 months, Hb did not significantly discriminate any concurrent or longitudinal outcomes. For Hb measured at age 12 months, we identified the best discriminatory thresholds for four outcomes ranging from 104 to 111 g/L (online supplemental table 4). Hb >106 g/L at 12 months was associated with a 6 percentage points higher prevalence of walking alone at 12 months (online supplemental table 4). Hb >111 g/L at 12 months was associated with 0.12 SD higher language score at 24 months. However, both of these associations were attenuated and became non-significant when adjusting for covariates. Hb >109 g/L at 12 months was associated with 0.16 SD higher socioemotional score at 24 months. Although a discriminatory Hb threshold was identified, no meaningful difference was found in 12-month concurrent motor score between subgroups above and below the threshold (105 g/L).

For Hb measured at age 15 months, we identified best discriminatory thresholds ranging from 97 to 105 g/L for three outcomes. Hb >104 g/L at 15 months was associated with 0.18 SD higher gross motor score at 15 months. No meaningful differences were found in 15-month language or socioemotional scores between subgroups above versus below the identified Hb thresholds.

For Hb measured at 18 months, we identified discriminatory thresholds for 12 outcomes. The best Hb thresholds ranged from 102 to 113 g/L for the nine concurrently measured outcomes, and from 116 to 117 g/L for the three longitudinal outcomes. For 18-month physical activity indicators, Hb >102 g/L was associated with a 0.38 SD higher MVM, Hb >106 g/L was associated with 0.30 SD lower % time in sedentary behaviour, and Hb >107 g/L was associated with 0.11 SD higher % time in MVPA. 18-month Hb >103 g/L was associated with 0.21 higher 18-month language score. For the 18-month socioemotional score, mean differences were 0.09–0.10 SD above versus below the identified best Hb thresholds (104–109 g/L; online supplemental table 4). For 18-month motor, gross motor and fine motor scores, mean differences above versus below the best Hb thresholds (104–113 g/L) ranged from 0.08 to 0.19 SD (online supplemental table 4). For the 18-month nap index and 24-month gross motor score, there was no meaningful difference above versus below the identified Hb thresholds (112–117 g/L). For 24-month outcomes, mean differences were 0.07–0.10 SD above versus below the best thresholds (116–117 g/L).

For Hb measured at 21 months, we identified best discriminatory thresholds for four outcomes ranging from 103 to 117 g/L. Language, socioemotional and motor scores at 21 months were 0.11–0.15 SD higher among children with Hb above 105 g/L compared with those with lower Hb; however, these associations were attenuated and became non-significant when adjusting for covariates. Hb >116 g/L at 21 months was associated with 0.16 SD higher 24-month language score; however, this difference was attenuated and became non-significant when adjusting for covariates.

For Hb measured at 24 months, best thresholds for three outcomes ranged from 107 to 108 g/L. Language, socioemotional and motor scores at 24 months were 0.10–0.16 SD higher among children with Hb above 107–108 g/L compared with those with lower Hb; however, the association of Hb with motor score was attenuated and became non-significant when adjusting for covariates.

For Hb measured at 27 months, we identified a discriminatory threshold for one outcome; Hb >115 g/L was associated with 0.12 SD higher concurrent gross motor score.

**Figure 2:**
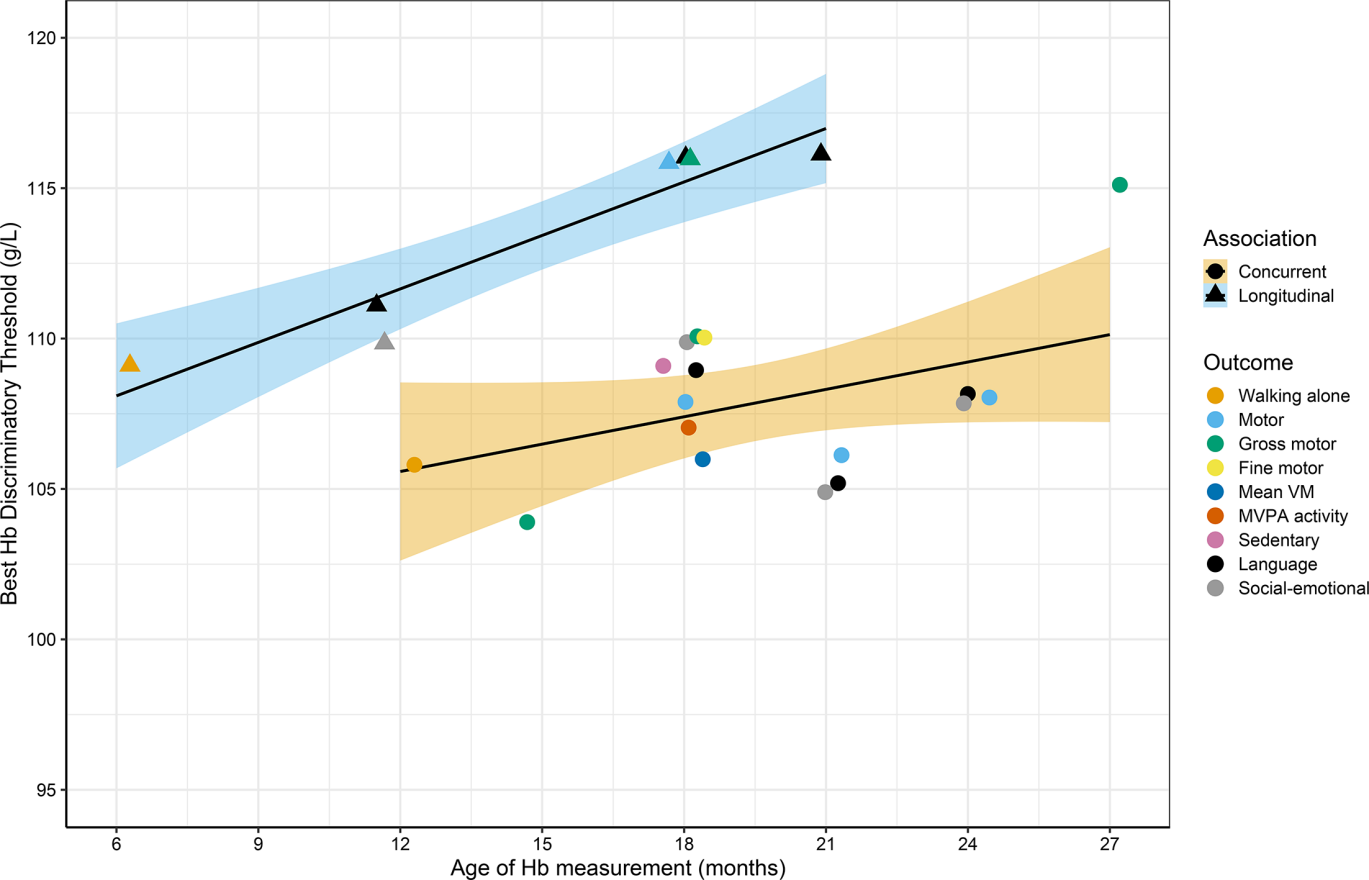
Best Hb discriminatory thresholds with a meaningful mean difference above versus below the threshold, by child age. *Trend lines are from simple linear regressions fitting selected Hb threshold on age of Hb measurement within the concurrent and longitudinal subgroups separately. The shaded regions are the 95% CIs around the regression line. Hb, haemoglobin; MVPA, moderate to vigorous physical activity; VM, vector magnitude.

**Figure 3:**
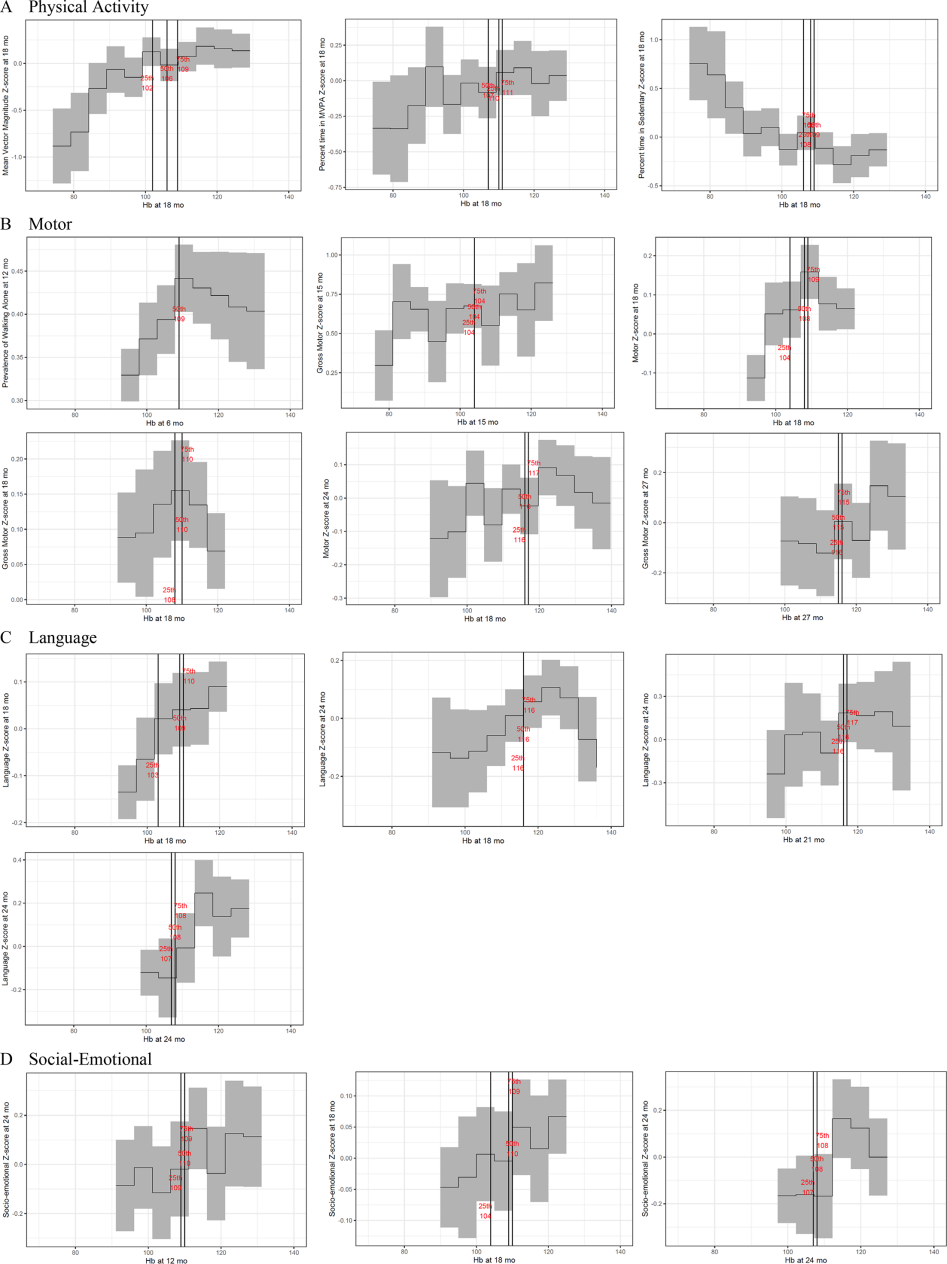
Bin plots showing the mean plus or minus one SE of the outcome in 5 g/L Hb bins. *Bin plots are shown for all outcomes that showed a meaningful difference in the outcome above versus below the best Hb threshold and were not attenuated when adjusting for covariates. Vertical lines showed the best Hb thresholds that discriminated each percentile of the outcome (25th, 50th and 75th). The x-axis range was harmonised across bin plots so that it could be compared across plots. The outcome means presented do not cover the full range of Hb in every plot because each bin was required to include participants from every study that contributed to that pooled analysis. Since the range of Hb varied across studies, the cut-off for the rightmost bin was defined as the top 2.5 percentile Hb value that was the lowest across studies. All Hb values that were higher than this cut-off were included in the rightmost bin. The cut-off for the leftmost bin was defined as the bottom 2.5 percentile Hb value that was the highest across studies. All Hb values that were lower than this cut-off were included in the leftmost bin. The y-axis range was allowed to vary across plots due to variance in the range of outcome values across different outcomes. Hb, haemoglobin.

In summary, out of 32 outcomes for which Hb thresholds were identified through the AUC analysis, 24 showed meaningful differences in outcomes above versus below the Hb threshold (figure 2), with 8 outcomes demonstrating an inverse U-shape through visual inspection of the bin plot (figure 3). For these eight Hb-outcome combinations, after repeating the AUC and mean differences analysis steps in the upper and lower ranges (see Methods section), we did not find any meaningful difference in outcomes in the upper versus mid-range of Hb. Specifically, in the upper range of Hb, only two pooled AUCs were significant (18-month Hb predicting 18-month fine motor and 24-month language); however, in both cases the mean difference in the outcome between subgroups in the upper versus mid-range of Hb was small (0.05 SD) and non-significant.

### Heterogeneity across contexts

The I^2^ for the pooled AUC analysis was low (I^2^≤0.40) for 61/92 (66%) analyses, moderate (0.40<I^2^≤0.6) for 12/92 (13%) analyses and substantial (I^2^>0.60) for 19/92 (21%) analyses (online supplemental table 4), which indicates generally low to moderate heterogeneity across studies in whether or not continuous Hb significantly discriminated between different levels of the outcome. For further details, see online supplemental results.

For the SD of the individual study best thresholds, this was less than 2 g/L for only 15/92 (16%) analyses. For 21/92 (23%) analyses, the SD was 2–5 g/L, while for the majority of analyses, the SD was greater than 5 g/L (56/92; 61%), indicating substantial heterogeneity in the identified best Hb threshold across studies.

### Sensitivity and stratified analyses

Results of random effects models were similar to those of fixed effect models (online supplemental table 3 and 5). Results of models stratified by intervention group were also similar. For all stratified analyses, there was no clear pattern of Hb better discriminating outcomes in either subgroup. For details, see online supplemental results.

## Discussion

We used a novel pooled analysis approach to identify the Hb thresholds that best discriminated several functional outcomes among 11 studies of 27 626 children aged 6–30 months in 8 LMICs. Of the three types of outcomes examined, physical activity and child development, but not sleep indicators, showed meaningful differences between subgroups above versus below best Hb thresholds. For concurrent associations, the best Hb thresholds with meaningful mean differences in the outcome above versus below the threshold ranged from 103 to 110 g/L for developmental outcomes measured at 12–24 months (with one 27-month best Hb threshold at 116 g/L), and ranged from 102 to 111 g/L for physical activity outcomes, which were measured at age 18 months only. In the longitudinal analyses, the best Hb thresholds with meaningful mean differences ranged from 109 to 117 g/L for developmental outcomes.

Although the best Hb thresholds covered a range of values, we observed several consistent patterns. First, the best Hb thresholds for longitudinal associations tended to be higher than the best thresholds for concurrent associations. This suggests that children with a marginally low Hb in the range 105–110 g/L may not show concurrent faltering in development, but may falter in the subsequent 6 to 18 months. However, children in this marginal range showed concurrent deficits in physical activity. The data show a striking pattern of MVM and % time spent in MVPA sloping downward, as well as % time spent in sedentary behaviour sloping upward, below the best Hb thresholds of 102 to 111 g/L (figure 3A). While the longitudinal analyses have the advantage of stronger inference of causality, they have the disadvantage that the Hb measurements were taken 6 to 18 months before the outcome measurements during a period in which Hb is likely to fluctuate over time, introducing noise. Concurrent Hb is likely an accurate indicator of Hb during the past few weeks. Thus, both types of analyses add novel evidence to be considered in defining anaemia.

Second, the best Hb thresholds to discriminate functional outcomes tended to be lower for younger children. This pattern is consistent with a study that identified the lowest fifth percentile of Hb values among apparently healthy children in 22 population-based surveys. The lowest fifth percentile increased with age across the range 6 to 59 months.^27^ This suggests that the cut-off to define abnormally low Hb should be lower for infants and young children compared with older children. This is also consistent with a review of 12 studies that reported Hb reference values for children aged 6–12 months defined based on the distribution of a healthy sample, all of which used a cut-off to define anaemia that was lower than 110 g/L, ranging from 68 to 105 g/L.^28^

We explored the possibility that values in the higher range of Hb may be associated with lower functional outcomes. A study in the UK found that Hb at 8 months of age above 150 g/L was associated with lower gross and fine motor scores at age 18 months.^29^ Although some bin plots seemed to show an inverse U-shape (figure 3), we did not find any Hb thresholds that meaningfully discriminated the upper versus the mid-range of Hb in this smaller subsample. The range of Hb among children included in our analysis was lower, on average, compared with samples of children in high-income countries. Future studies in populations with a greater representation of children with high Hb should further explore this possibility of functional outcomes sloping downward in the upper range of Hb.

There are several potential explanations for associations between Hb and functional outcomes. Anaemia may be a marker of an environment that is associated with deficits in the outcome, such as poverty. We explored this possibility by adjusting mean differences between subgroups above versus below the best Hb thresholds for socioeconomic status and other background characteristics. Generally, we found slightly smaller mean differences between high and low Hb groups in adjusted compared with unadjusted analyses (online supplemental table 4). Out of the 24 outcomes that showed meaningful differences between subgroups above versus below the identified Hb thresholds, 16 associations remained significant when adjusting for covariates. This suggests that Hb generally was not simply a marker for poverty.

Another possibility is that low Hb is a marker of an underlying condition that causes deficits in the outcome, such as iron deficiency or malaria. In the majority of stratified analyses, results for the AUC and mean difference analyses were similar in subgroups of children with iron deficiency or inflammation. Our findings suggest that children with malaria experienced worse functional outcomes at low Hb concentrations. These analyses, however, were exploratory and do not allow us to draw firm conclusions.

We observed substantial heterogeneity in the Hb value that best discriminated outcomes across datasets. Several factors may account for heterogeneity across contexts. First, the aetiology of anaemia may differ in different contexts due to both genetic and environmental factors.^30^ As just described, we were not able to draw conclusions from analyses stratified by factors such as iron deficiency, and we were not able to explore other factors, such as genetic Hb disorders. Therefore, further studies disaggregated by different aetiologies of anaemia are needed to understand how anaemia aetiology affects associations with functional outcomes. Second, methods to measure Hb differed across trials, with five trials using venous samples and six using single drop capillary samples. Hb measured from capillary samples, particularly single drop, has been found to be associated with substantial imprecision compared with Hb based on venous samples.^31 32^ Imprecision in Hb measurements may have caused us to underestimate associations of Hb with outcomes in some datasets by introducing noise. However, Hb measured from single drop capillary samples is widely used, thus our results reflect associations that would be typical for the blood collection methods that are currently used in public health practice. Furthermore, Hb estimates from two included trials, in Kenya and Zimbabwe, adjusted Hb values for altitude, which also introduced heterogeneity between datasets. These were the only two study sites in high altitude settings. We expected that high-altitude adjusted Hb would be more comparable to values from lower-altitude settings, therefore more appropriate for our aims than unadjusted values.

This pooled analysis had many strengths. A substantial number of high-quality datasets from LMICs were included and the sample size was very large. The availability of individual participant data allowed harmonisation of calculation of outcomes across trials. We developed and applied novel methodology to examine our research questions using a robust pooled analysis approach. The consistency of findings across fixed and random effects models and the sensitivity analysis by intervention group strengthens confidence in the conclusions. This pooled analysis also had limitations. Although we were able to harmonise the calculation of developmental assessment scores across trials, different tools were used in different trials, and we did not have an external reference to standardise scores. Thus, although all developmental scores were calculated in units of SD based on the within-study distribution, if SDs varied across studies, the point value of 1 SD could be larger in one trial than another, and cut-offs at the 25^th^, 50^th^ and 75^th^ percentile could represent different developmental ability levels. Given the need to pool the data, however, the approach used was considered the most appropriate option. Additionally, the range of Hb in our datasets may be lower than in a healthy population, which could limit our ability to detect any potential deficit in outcomes at high Hb concentrations. Some analyses had few studies contributing to the pooled analysis, which could bias the I^2^ and reduce the generalisability of the results. Lastly, the majority of studies included in this pooled analysis were from sub-Saharan Africa, and only two from South Asia (Bangladesh), potentially limiting generalisability of findings to other contexts.

In conclusion, the current WHO cut-offs to define anaemia among children (Hb <105 g/L in children 6–23 months and Hb <110 g/L in children 24–59 months), which were based on the distribution of Hb in a healthy population, are in the middle to upper range of the Hb values that best discriminate concurrent physical activity and development, and are in the lower range of values that best discriminate subsequent child development. How this evidence of best Hb discriminatory thresholds is weighed in the context of other evidence by various guideline development groups will depend on their goals and priorities. For example, a group may choose to prioritise certain types of evidence or outcomes over others, or may make different decisions depending on the purpose of the cut-off and whether it will be used in a specific context or across contexts. Our study provides novel evidence to inform such decisions, as well as novel pooled analysis methodology to identify thresholds of continuous biomarkers that best discriminate functional outcomes.

## Supplementary material

10.1136/bmjgh-2024-015866 online supplemental file 1

## Data Availability

No data are available.
