# Supplementary material for: Haemoglobin concentration thresholds that discriminate functional outcomes among children aged 6–30 months in eight countries: a pooled analysis of individual participant data
Source: BMJ Glob Health. 2026 Apr 28;11(4):e015866. doi: 10.1136/bmjgh-2024-015866 (PMC13141236; doi:10.1136/bmjgh-2024-015866)

Supplemental Material for the Manuscript “Hemoglobin concentration thresholds that discriminate functional outcomes among children aged 6-30 months in 8 countries: A pooled analysis of individual participant data”

Supplemental Methods

*Objectives*

Our second objective was to describe the shape of the association of Hb with each outcome. While determining the best Hb threshold identifies which Hb value best discriminates the outcome, understanding how the association of Hb with the outcome may change above versus below that threshold requires further data visualization. For example, it is possible that the discriminatory threshold identified through the AUC analysis represents a plateau point at which the association of Hb with the outcome begins to slope downwards. Alternatively, it is possible that the outcome increases across the full range of Hb, both above and below the discriminatory threshold. Another possibility is that the outcome slopes downward at both higher and lower values of Hb (inverted U-shape).

*Inclusion and exclusion criteria for this pooled analysis*

Eligible studies were those that (1) were included in the previous individual participant data (IPD) meta-analysis of small-quantity lipid-based nutrient supplements (SQ-LNS),^1^ (2) measured child Hb concentration and at least one outcome of interest concurrent with or subsequent to Hb assessment, and (3) whose investigators agreed to participate in this analysis.

*Data collection*

We invited all principal investigators of eligible studies to participate in this pooled analysis. For the previous IPD meta-analysis of effects of SQ-LNS, we had provided to investigators a data dictionary listing definitions of variables requested for pooled analysis. For this pooled analysis, the dataset was already compiled, therefore we did not request additional data from the investigators.

*Pooled analysis integrity*

We checked data for completeness by evaluating whether the study sample sizes in our pooled dataset were the same as in study protocols and publications. We also checked summary statistics, such as means and standard deviations (SD), in our dataset against published values for each trial. Implausible values were inspected for errors and truncated to 5 or -5 SD from the mean z-score (≤0.2% of values for each outcome, with the highest percentage for motor, gross and fine motor scores: 0.18-0.20%).

*Specification of outcomes*

*Physical activity.* In the iLiNS-DYAD-M and iLiNS-DOSE trials, 18-month-old children wore Actigraph GT3X+ accelerometers for 7 days.^2 3^ The ActiGraph GT3X device is a small tri-axial accelerometer, which records accelerations in three different axes: vertical, anterior-posterior, and medial-lateral. Vector magnitude is a variable that combines information from these three axes and is calculated by taking the square root of the sum of the squared activity counts of each axis. In both trials, the validated cut points used for the hip-worn accelerometers were ≤48 counts/15 sec for sedentary behavior and ≥419 counts/15 sec for moderate-to-vigorous physical activity, based on the vertical axis. A measurement day was considered valid if it had a minimum of six hours of accelerometer data between 5:00 am and 8:00 pm, after excluding strings of ⩾20 min of zeroes. Participants with ⩾4 valid days of data were included in the analyses.

*Sleep*. In the iLiNS-DYAD-G^4^ and iLiNS-DYAD-M^5^ trials sleep was quantified as a (1) night time sleep duration index and (2) day time nap index at age 6, 12, and 18 months as follows. The night time sleep duration was defined as the number of hours between the time the child went to bed (for the overnight sleep) and the time the child woke up in the morning. Daytime nap was defined as a nap taken after s/he had woken up from the previous night’s sleep and before s/he went to bed that evening. In the iLiNS-DYAD-G trial, sleep data were collected at age 6, 12, and 18 months, including parent-reported usual duration of night time sleep and the number of naps in the past 24 hours. In the iLiNS-DYAD-M trial, sleep data were collected monthly from 6 to 18 months. Night time sleep duration index was calculated as the mean of parent-reported duration of night time sleep during the past 24 hours for all available forms collected at age (1) 6-9 months, (2) 11-13 months, and (3) 16-18 months. Day time nap index was calculated as the mean of parent-reported number of day time naps during the past 24 hours for all available forms collected at age (1) 6-9 months, (2) 11-13 months, and (3) 16-18 months. Sleep outcomes were standardized in the same way as the developmental outcomes (standardized residual by child age and sex).

*Developmental outcomes.* Language score was defined as the total language score calculated according to the established method for the tool used in each study. Motor score was defined as the total motor score calculated according to the established method for the tool used in each study (total of both gross and fine motor). Social-emotional score was defined as the total social-emotional score calculated according to the established method for the tool used in each study. If a higher score reflects greater social-emotional difficulties, this score was reversed so that for all scores a higher score will reflect better performance. Gross motor score was defined as the total gross motor score calculated according to the established method for the tool used in each study. Fine motor score was defined as the total fine motor score calculated according to the established method for the tool used in each study. If any study used multiple tools or scores to assess the same domain (e.g. both DMC and CDI to assess language), we used the tool or score that was used in the greatest number of other studies.

Z-scores were standardized within each study by regressing the unstandardized developmental score on child age and sex and calculating the standardized residuals. This approach is analogous to calculating length-for-age z-score (LAZ) in that the score represents deviations from the predicted score for a given child’s age and sex in units of standard deviation. However, developmental outcome z-scores were calculated in reference to each within-study distribution, rather than an external standard. For example, a female child with a language z-score of -1 scored one SD below the predicted value for female children of the same age in her study sample. Given that different tools were used in different trials and we did not have an external reference to standardize scores, this method allowed harmonization of the calculation of developmental assessment scores across trials. An advantage of using this method is that the score in every trial has the same meaning (i.e., a child’s score in relation to other children in the study sample in units of SD). However, a disadvantage is that if SDs varied across studies, the point value of 1 SD could be larger in one trial than another. For example, among the 5 studies that used a 100-word vocabulary checklist to assess language, SDs ranged from 18.9 to 23.5. Ongoing efforts to develop a standardized scale of developmental scores will greatly improve future meta-analyses of developmental outcomes.^6^

*Walking alone at age 12 months*. We used reports or observations of the child’s ability on the day of assessment, not retrospective reports of age of milestone achievement, as the latter is subject to potential recall inaccuracy. If both observation and parent report data were collected at the same time point, we used observation data.

*Specification of ages of interest*

We stratified the datasets by child age and analyzed all ages (6, 9, 12, 15, 18, 21, 24, 27, 30 months) for which the minimum samples size was available (see below). For each time point, we included data collected within 1.5 months of the target age. We used the following three criteria to determine the minimum sample size for any given analysis. (1) Data must be available from at least 30 children for a study to contribute data to an analysis. An N of 30 is the minimum number of observations to reasonably assume the distribution of sample means will be approximately normal.^7^ (2) At least two studies must contribute data to the analysis. (3) The total sample size for the analysis must be at least 352 children. This is the minimum sample size to detect a 2-group mean difference of 0.3 SD with 80% power and alpha of 0.05.

At each age, we examined (1) concurrent discriminatory thresholds, i.e., associations of child Hb with concurrently measured functional outcomes and (2) longitudinal discriminatory thresholds, i.e., associations of child Hb with subsequently measured functional outcomes. There are advantages and disadvantages of both the concurrent and longitudinal analyses. While longitudinal associations are considered to have stronger inference of causality than concurrent associations, a disadvantage of our longitudinal analyses is that Hb can fluctuate widely over time during early childhood. Thus, the longitudinal analyses introduce more noise because the outcomes were measured 6 to 12 months after Hb was measured. Concurrent Hb is likely a more accurate reflection of Hb in the past few weeks than Hb 12 months ago. For some outcomes, the onset of the deficit is likely to coincide with the onset of anemia, for example an increase in sedentary behavior due to fatigue. Thus, both the concurrent and longitudinal results add meaningful information to answer our research questions and inform Hb cut-offs to define anemia.

*Synthesis methods and exploration of variation in effects*

For the first objective, we used area under the receiver operating characteristic curve (AUC) analysis to determine what Hb values best discriminated higher versus lower functional outcomes (step 1). Since this analysis requires binary outcomes, for each continuous outcome, we used three cut-offs: less than the 25^th^ percentile, 50^th^ percentile, and 75^th^ percentile. We used these three cut-offs for several reasons. First, our aim was to determine inflection points in the association of Hb with functional outcomes using a data-driven approach, therefore we did not have an a priori hypothesis where along the outcome continuum the inflection point might fall. Second, there is no standard developmental norm across contexts, therefore no universal definition of normal versus abnormal status. In addition, each developmental score was standardized within each study sample (see above), thus the 25^th^ percentile in one study may not be comparable to the 25^th^ percentile in another study. The 25^th^ percentile in any given study sample also may not be comparable to the 25^th^ percentile in a healthy population, given that many children in LMICs experience multiple risk factors for poor child development. Among the 9 studies in our analysis that reported the prevalence of walking alone at age 12 months, 7 were lower than the prevalence expected in a healthy population, which is 50% based on the WHO norms. In those 7 studies, the prevalence ranged from 12 to 40%, while two studies had a prevalence of 49-52% of children walking alone at 12 months (Supplemental Table 1). Including all three cut-offs (25^th^, 50^th^, and 75^th^ percentile) for the continuous developmental outcomes allowed us to examine potential inflection points across the full range of the outcome distribution.

If none of the three pooled AUCs (for the 25^th^, 50^th^, and 75th percentile) were able to discriminate between different levels of the outcome (pooled AUC 95% CI contains 0.5), we concluded that Hb did not discriminate between higher and lower values of that outcome at that time point and did not conduct further analyses.

For the mean difference analysis (step 3), for dealing with participant dependence in clustered trials we used robust standard errors with clusters as the independent units.

To describe the shape of the association between Hb and each outcome (Objective 2), we used bin plots instead of splines because spline plots did not permit us to objectively and precisely identify peaks and troughs. Bin plots are also less influenced by outliers than spline plots. In order to pool means across studies, we specified 5 g/L bins that were common across studies by constructing a set of potential bin combinations, fitting linear models within each study, calculating the total Akaike Information Criterion (AIC) across studies, and selecting the bin combination that maximized the total AIC. Then, the mean and 95% CI of the outcome within each bin was calculated within each study and pooled using inverse-variance weighted random effects to generate a pooled bin plot.

For any association that showed an inverse U shape, we conducted exploratory analyses to determine whether there was a value in the upper range of Hb that significantly discriminated the outcome. The following Figure shows an association with an inverse U-shape. In this example, we subset the data into two overlapping samples: (1) All Hb values above 116 and (2) all Hb values below 130. We repeated all Objective 1 analysis steps (described in the main text and summarized in the main text Figure 1) on these two subsets separately to determine the Hb values, if any, that best discriminated the upper (versus middle) and lower (versus middle) ranges of the Hb distribution.


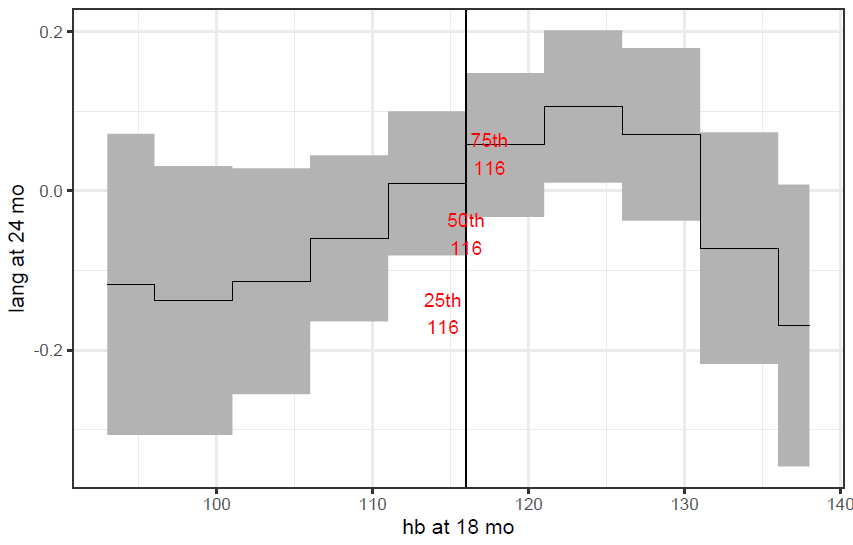


*Sensitivity analyses*

Associations between Hb and functional outcomes may differ by intervention group for several reasons, for example, the SQ-LNS intervention included nutrients that can affect brain development independent of changes in Hb.

*Stratified Analyses*

All stratified analyses were considered exploratory. We stratified by child sex because physiological differences between males and females may lead to differences in associations of Hb with outcomes between male and female children. We stratified by iron deficiency, malaria, and inflammation because these are potential underlying causes of anemia and associations of Hb with functional outcomes may differ by anemia etiology. We stratified by HIV exposure because developmental outcomes tend to be lower in HIV-exposed compared to unexposed children, which could also change the association of Hb with developmental outcomes. Lastly, we stratified by type of blood sample (i.e., venous or capillary) because emerging evidence suggests that hemoglobin concentration measured using capillary blood is lower than hemoglobin concentration measured using venous blood.^8^

Adequate sample size in the main analysis was considered sufficient criterion to conduct the stratified analyses and sensitivity analyses. We also applied two of the three criteria we used for the main analyses to determine the minimum sample size for the stratified analyses. (1) Data must be available from at least 30 children for a study to contribute data to an analysis. An N of 30 is the minimum number of observations to reasonably expect an analysis based on a normal distribution is valid. (2) At least two studies must contribute data to the analysis. However, we did not apply the third criterion that the total sample size for the analysis must be at least 352 children. For the stratified analyses, we used the same cut-off to define the 25^th^, 50^th^, and 75^th^ percentile of the outcome as the main analysis, instead of re-calculating these cut-offs on the subsample for which data were available for the stratified analysis.

Supplemental Results

*Pooled analysis integrity*

After truncating implausible values to 5 or -5 SD from the mean z-score (≤0.2% of values for each outcome, with the highest percentage for motor, gross and fine motor scores: 0.18-0.20%), no other issues were identified.

*Heterogeneity across contexts*

We also assessed the percentage of the variability in effect estimates that is due to heterogeneity by calculating the I^2^ for the pooled mean difference or prevalence difference. This indicates heterogeneity across studies in whether there was a significant difference in the outcome above versus below the identified Hb threshold. This type of heterogeneity was low to moderate (I^2^ ≤ 0.60) for 72/92 (78%) analyses and substantial (I^2^ > 0.60) for 20/92 (22%) analyses. We did not report these in Supplemental Table 4 because the results were similar to the I^2^ for the pooled AUC, therefore these results did not add to the interpretation or conclusions. Results are available from the authors upon request.

*Differences between the 25^th^, 50^th^, and 75^th^ quartile-derived binary outcomes*

The best Hb thresholds to discriminate the 50^th^ and 75^th^ percentile of the outcome were similar; of the 15 continuous outcomes shown in Figure 2, these were within 1 g/L for 12 (80%) analyses and differed by 3-4 g/L for 3 analyses. The best Hb value to discriminate the lowest 25^th^ percentile of the outcome tended to be lower. Although this was also within 1 g/L of the best Hb threshold for the 50^th^ percentile for 9/15 (60%) analyses, it differed by 2-6 g/L in 6 analyses.

*Sensitivity analyses*

Of the 32 associations for which Hb significantly discriminated the outcome (pooled AUC did not contain 0.5 for at least one cut-off) in fixed effect models (21 concurrent and 11 longitudinal associations), conclusions from random effects models were similar to fixed effect models, with Hb significantly discriminating the outcome for 18 concurrent and 9 longitudinal associations. Of the 24 unadjusted mean differences that were considered meaningful in fixed effect models (MD ≥ 0.07 and p < 0.1; Supplemental Table 4), 20 were also meaningful in random effects models.

Of the 92 AUCs reported in Supplemental Table 4, the AUCs stratified by intervention group were similar (within 0.03 of the overall AUC) for 75 AUCs (82%) for the control group and 68 AUCs (75%) for the intervention group. Among the AUCs that were not similar (absolute difference > 0.03), 16 were closer to 0.5 (no discrimination) in the intervention group and 13 were closer to 0.5 in the control group. Thus there was no clear pattern that Hb was better able to discriminate the outcome in one group or the other, and in the majority of cases the ability of Hb to discriminate the outcome was similar between groups.

We also examined the interaction of intervention group with Hb group (above versus below the identified cut-off) for the mean difference analysis to determine whether the mean differences in outcomes above versus below the identified Hb cut-offs were significantly different in the intervention versus control group. These interactions were significant for six analyses, three showing greater Hb group differences in the intervention group (association of 6-mo Hb with 12-mo sleep index, 18-mo Hb with 18-mo % time in sedentary behavior and 18-mo mean vector magnitude) and three showing greater Hb group differences in the control group (association of 18-mo Hb with 18-mo motor score, 21-mo Hb with 24-mo language and 24-mo Hb with 24-mo motor score). Thus, we again concluded that there was no consistent pattern of greater associations of Hb with outcomes in one group or the other.

*Stratified analyses*

Stratified by child sex, the AUCs were similar (within 0.03 of the overall AUC) for 81/92 AUCs (88%) for females and 82/92 AUCs (89%) for males. Among the AUCs that were not similar (absolute difference > 0.03), six were closer to 0.5 (no discrimination) among females and seven were closer to 0.5 among males. Thus, there was no clear pattern of Hb better discriminating outcomes in males or females. The interaction of child sex with Hb group (above versus below the identified cut-off) was significant for three mean differences (concurrent associations of 15-mo Hb with language and social-emotional scores and 18-mo Hb with gross motor scores). All of these showed effects in the expected direction among males (higher Hb associated with better outcomes) and in the opposite direction among females. However, in the majority of cases the interactions were not significant, suggesting no differences between males and females.

Stratified by child iron deficiency, data were available to calculate 22 AUCs among non-iron deficient children and 10 among iron-deficient children. Among non-iron deficient children, AUCs were similar (within 0.03 of the overall AUC) for 16/22 AUCs (73%). For iron-deficient children, AUCs were similar for 7/10 AUCs (70%). Among the AUCs that were not similar (absolute difference > 0.03), six were closer to 0.5 (no discrimination) among iron-deficient children and three were closer to 0.5 among non-iron-deficient children. Thus, there was no clear pattern of Hb better discriminating outcomes in either group. The interaction of iron deficiency with Hb group was significant for two mean differences (concurrent associations of 18-mo Hb with language and social-emotional scores), both of which showed a greater difference between Hb groups among non-iron-deficient children.

Stratified by child malaria infection, data were available to calculate 37 AUCs among children without malaria and 24 among children with malaria. Among children without malaria, all (100%) AUCs were similar, within 0.03 of the overall AUC. For children with malaria, AUCs were similar for only 5/24 AUCs (21%). Among the AUCs that were not similar (absolute difference > 0.03), 14 were closer to 0.5 (no discrimination) among children without malaria and 5 were closer to 0.5 among children with malaria. This suggests that Hb may better discriminate outcomes among children with malaria. The interaction of malaria with Hb group was significant for five mean differences (concurrent associations of 18-mo Hb with language, motor, nap index, MVM, and % time in sedentary behavior). Three of these showed greater differences among children with malaria (language, motor, and nap index), while two showed greater differences among children without malaria (MVM and % time in sedentary behavior).

Stratified by inflammation, data were available to calculate 61 AUCs among children who did not have high CRP and 49 among children who had high CRP. Among children without inflammation, AUCs were similar (within 0.03 of the overall AUC) for 52/61 AUCs (85%). For children with inflammation, AUCs were similar for 33/49 AUCs (67%). Among the AUCs that were not similar (absolute difference > 0.03), 17 were closer to 0.5 (no discrimination) among children without high CRP and 7 were closer to 0.5 among children with high CRP. The interaction of inflammation with Hb group was significant for four mean differences (association of 6-mo Hb with 12-mo sleep index, and 18-mo Hb with 18-mo MVPA, and 24-month language and gross motor scores). Two of these showed a greater difference between Hb groups among children without inflammation (language and gross motor), while the other two showed a greater difference between Hb groups among children with inflammation.

Stratified by HIV exposure, sufficient data were not available to conduct the analysis.

In addition to participant level stratification above, we also stratified analyses on study blood collection method. Unfortunately, stratification by blood collection method limited available sample size to conclusively compare differences driven by blood sample type. Specifically, data were available to calculate 25 AUCs among studies with venous blood collection and 128 among studies with capillary blood collection. Among studies with venous blood collection, AUCs were similar (within 0.03 of the overall AUC) for 22/25 AUCs (88%). For studies with capillary blood collection, AUCs were similar for 154/154 AUCs (100%). Among the venous blood collection AUCs that were not similar, 2 were closer to 0.5 (no discrimination) and 1 had improved discrimination.

References

1. Dewey KG, Stewart CP, Wessells KR, et al. Small-quantity lipid-based nutrient supplements for the prevention of child malnutrition and promotion of healthy development: overview of individual participant data meta-analysis and programmatic implications. *The American Journal of Clinical Nutrition* 2021;114(Supplement_1):3S-14S. doi: 10.1093/ajcn/nqab279 [published Online First: 2021/10/01]

2. Pulakka A, Ashorn U, Cheung YB, et al. Effect of 12-month intervention with lipid-based nutrient supplements on physical activity of 18-month-old Malawian children: a randomised, controlled trial. *Eur J Clin Nutr* 2015;69(2):173-8. doi: 10.1038/ejcn.2014.138

3. Pulakka A, Cheung YB, Maleta K, et al. Effect of 12-month intervention with lipid-based nutrient supplement on the physical activity of Malawian toddlers: a randomised, controlled trial. *Br J Nutr* 2017;117(4):511-18. doi: 10.1017/S0007114517000290 [published Online First: 2017/04/07]

4. Adu-Afarwuah S, Lartey A, Okronipa H, et al. Small-quantity, lipid-based nutrient supplements provided to women during pregnancy and 6 mo postpartum and to their infants from 6 mo of age increase the mean attained length of 18-mo-old children in semi-urban Ghana: a randomized controlled trial. *Am J Clin Nutr* 2016;104(3):797-808. doi: 10.3945/ajcn.116.134692

5. Ashorn P, Alho L, Ashorn U, et al. The impact of lipid-based nutrient supplement provision to pregnant women on newborn size in rural Malawi: a randomized controlled trial. *Am J Clin Nutr* 2015;101(2):387-97. doi: 10.3945/ajcn.114.088617

6. Weber AM, Rubio-Codina M, Walker SP, et al. The D-score: a metric for interpreting the early development of infants and toddlers across global settings. *BMJ Glob Health* 2019;4(6):e001724. doi: 10.1136/bmjgh-2019-001724 [published Online First: 2019/12/06]

7. Hogg RV, Tanis EA, Zimmerman DL. Probability and statistical inference. Upper Saddle River, NJ: Pearson/Prentice Hall, 2010.

8. Larson LM, Braat S, Hasan MI, et al. Preanalytic and analytic factors affecting the measurement of haemoglobin concentration: impact on global estimates of anaemia prevalence. *BMJ global health* 2021;6(7):e005756. doi: 10.1136/bmjgh-2021-005756

*List Supplemental Tables and Figures*

**Supplemental Table 1**. Descriptive information by trial

**Supplemental Table 2**. Ages at which Hb and outcomes were measured in each trial

**Supplemental Table 3**. Pooled AUCs for all Hb-outcome associations that met the minimum sample size criteria

**Supplemental Table 4**. Best Hb discriminatory thresholds that were identified for each outcome and age.

**Supplemental Table 5**. Mean difference in Hb above vs below candidate Hb threshold for all Hb-outcome associations that met the minimum sample size criteria

**Supplemental Figure 1**. Hb distributions by trial

| **Supplemental Table 1: Descriptive information, by trial** | | | | | | | |  |  |  |  |  |  |  |  |
| --- | --- | --- | --- | --- | --- | --- | --- | --- | --- | --- | --- | --- | --- | --- | --- |
|  |  |  |  |  |  |  |  |  |  |  |  |  |  |  |  |
| **Country** | **First author, year** | **N children** | **Region** | **Maternal age: Mean (SD)** | **Number of children under 5 years:  Mean (SD)** | **Child sex, male (%)** | **Child birth order, first born (%)** | **Iron deficiency at last biomarker assessment (%)** | **Malaria at last biomarker assessment (%)** | **Inflammation at last biomarker assessment (%)** | **Walking alone at 12 mo (%)** | **Stunting at 18 mo (%)** | **Moderate to severe food insecurity (%)** | **Improved source water quality (%)** | **Improved sanitation access (%)** |
| Bangladesh | Dewey, 2017 | 3504 | SEAR | 21.9 (4.9) | 0.42 (0.60) | 50.3 | 39.7 | 15.6 | - | 12.9 | 31.3 | 34.7 | 37.6 | 100.0 | 70.9 |
| Bangladesh | Luby, 2018 | 4572 | SEAR | 23.9 (5.2) | 0.65 (0.88) | 49.9 | 33.6 | 17.7 | - | 8.9 | 26.7 | 40.8 | 21.9 | 87.2 | 94.3 |
| Burkina Faso | Hess, 2015 | 1121 | AFR | 27.1 (6.7) | 1.00 (1.01) | 50.4 | 21.8 | 43.0 | 50.4 | 29.1 | - | 32.0 | 50.8 | 26.9 | 2.4 |
| Ghana | Adu Afarwuah,2007 | 359 | AFR | 28 (5.8) | 0.54 (0.89) | 50.1 | 40.0 | 31.4 | 3.7 | - | 34.3 | - | - | 92.5 | 92.1 |
| Ghana | Adu Afarwuah, 2016 | 1141 | AFR | 26.7 (5.4) | 0.51 (0.64) | 48.6 | 33.3 | - | 1.3 | 14.6 | 49.2 | 11.5 | 31.4 | 98.3 | 97.4 |
| Kenya | Null, 2018 | 6366 | AFR | 26.3 (6.3) | 0.61 (0.65) | 48.2 | 20.9 | 40.6 | 18.5 | 16.6 | 40.0 | - | 10.3 | 67.6 | 16.0 |
| Madagascar | Galasso, 2019 | 4049 | AFR | 26.5 (7.6) | 0.93 (0.91) | 49.7 | 27.7 | 31.7 | - | 16.5 | 12.6 | 59.4 | 32.7 | 28.4 | 0.0 |
| Malawi | Ashorn, 2015 | 722 | AFR | 25.1 (6.0) | 0.34 (0.69) | 47.3 | 21.1 | - | 5.7 | 28.3 | 52.1 | 36.2 | 69.7 | 91.2 | 9.9 |
| Malawi | Maleta, 2015 | 1542 | AFR | 26.4 (6.4) | 0.65 (0.71) | 50.0 | 23.2 | - | 5.9 | 35.1 | 35.4 | 43.7 | 72.7 | 92.2 | 1.9 |
| Mali | Huybregts, 2019 | 2289 | AFR | 25.8 (6.7) | 0.75 (0.86) | 52.0 | 14.8 | - | - | - | 29.4 | 37.8 | 2.0 | 60.1 | 74.9 |
| Zimbabwe | Humphrey, 2019;  Prendergast, 2019 | 1961 | AFR | 27.2 (6.8) | 0.69 (0.66) | 49.7 | 23.4 | - | - | - | - | 30.6 | 20.5 | 62.5 | 34.1 |
|  |  |  |  |  |  |  |  |  |  |  |  |  |  |  |  |
|  |  |  |  |  |  |  |  |  |  |  |  |  |  |  |  |
| Geograhic region based on WHO regions | | | | | | | |  |  |  |  |  |  |  |  |
| Number of children under 5 years excludes the child enrolled in the study. | | | | |  |  |  |  |  |  |  |  |  |  |  |
| Data on birth order were not available for all children. Consequently, first-born vs later-born status was estimated based on the number of children under 5 years old in the household. | | | | | | | | | | |  |  |  |  |  |
| Iron deficiency defined as inflammation corrected ferritin less than 12 ug/L | | | | |  |  |  |  |  |  |  |  |  |  |  |
| Inflammation is define as AGP > 1g/L or CRP > 5mg/L | | |  |  |  |  |  |  |  |  |  |  |  |  |  |
| Stunting is defined as WHO Length-For-Age Z-Score < -2 SD | | | |  |  |  |  |  |  |  |  |  |  |  |  |
| Improved water source includes piped water, boreholes or tubewells, protected dug wells or springs, rainwater, and packaged or delivered water based on WHO, UNICEF Joint Monitoring Program | | | | | | | | | | |  |  |  |  |  |
| Improved sanitation includes flush/pour flush to piped sewer system, septic tanks, or pit latrines; ventilated improved pit latrines, composting toilets, or pit latrines with slabs based on WHO, UNICEF Joint Monitoring PRogram | | | | | | | | | | | | |  |  |  |
| Food security scales used: a) Food Access Survey Tool; b) Household Food Insecurity Access Scale (HFAIS); c) Coping Strategy Index | | | | | | | | | | | | | | | |

**Supplemental Table 2**. Ages at which Hb and outcomes were measured in each trial

|  |  | **Age at Measurement** | |  |  |  |  |  |  |  |
| --- | --- | --- | --- | --- | --- | --- | --- | --- | --- | --- |
| **Country** | **First author, year** | **6 mo** | **9 mo** | **12 mo** | **15 mo** | **18 mo** | **21 mo** | **24 mo** | **27 mo** | **30 mo** |
| Bangladesh | Dewey, 2017 | Hb, Dev |  | Dev |  | Hb, Dev |  | Dev |  |  |
| Bangladesh | Luby, 2018 |  |  | Dev |  |  |  | Hb, Dev | Hb, Dev | Hb, Dev |
| Burkina Faso | Hess, 2015 |  | Hb |  |  | Hb, Dev |  |  |  |  |
| Ghana | Adu Afarwuah, 2007 | Hb |  | Hb, Dev |  |  |  |  |  |  |
| Ghana | Adu Afarwuah, 2016 | Hb, Sleep |  | Dev, Sleep |  | Hb, Dev, Sleep |  |  |  |  |
| Kenya | Null, 2018 |  |  | Dev |  | Hb | Hb, Dev | Hb, Dev | Dev | Dev |
| Madagascar | Galasso, 2019 | Hb, Dev | Hb, Dev | Hb, Dev | Hb, Dev | Hb, Dev | Hb, Dev | Hb, Dev | Hb, Dev | Hb, Dev |
| Malawi | Ashorn, 2015 | Hb, Sleep | Hb | Dev, Sleep |  | Hb, Dev, PA, Sleep |  |  |  |  |
| Malawi | Maleta, 2015 | Hb |  | Dev |  | Hb, Dev, PA | Dev |  |  |  |
| Mali | Huybregts, 2019 | Hb, Dev | Hb, Dev | Hb, Dev | Hb, Dev | Hb, Dev | Hb, Dev | Hb, Dev |  |  |
| Zimbabwe | Humphrey, 2019; Prendergast, 2019 | Hb | Hb | Hb | Hb | Hb | Hb | Hb, Dev | Hb, Dev |  |
| Hb indicates age category has at least 30 observations with hemoglobin measured | | | | |  |  |  |  |  |  |
| Dev indicates age category has at least 30 observations with development measured | | | | |  |  |  |  |  |  |
| PA indicates age category has at least 30 observations with physical activity measured | | | | |  |  |  |  |  |  |
| Sleep indicates age category has at least 30 observations with sleep patterns measured | | | | |  |  |  |  |  |  |

**Supplemental Table 3**. Pooled AUCs for all Hb-outcome associations that met the minimum sample size criteria

| **Age of Hb measurement** | **Age at outcome measurement** | **Outcome** | **Outcome percentile** | **Pooling method** | **Selected Hb (g/L)** | **N children** | **AUC (95% CI)** | **I^2^** | **SD of selected HB across studies** |
| --- | --- | --- | --- | --- | --- | --- | --- | --- | --- |
| 6 | 6 | Gross Motor Z-Score | 25th | Fixed | 107 | 1458 | 0.50 (0.47, 0.54) | 0.80 | 3.6 |
| 6 | 6 | Gross Motor Z-Score | 25th | Random | 107 | 1458 | 0.48 (0.37, 0.59) | 0.80 | 3.6 |
| 6 | 6 | Gross Motor Z-Score | 50th | Fixed | 105 | 1458 | 0.49 (0.46, 0.52) | 0.68 | 3.1 |
| 6 | 6 | Gross Motor Z-Score | 50th | Random | 105 | 1458 | 0.49 (0.41, 0.57) | 0.68 | 3.1 |
| 6 | 6 | Gross Motor Z-Score | 75th | Fixed | 105 | 1458 | 0.49 (0.46, 0.52) | 0.00 | 0.6 |
| 6 | 6 | Gross Motor Z-Score | 75th | Random | 105 | 1458 | 0.49 (0.46, 0.52) | 0.00 | 0.6 |
| 6 | 6 | Nap Index Z-Score | 25th | Fixed | 108 | 1544 | 0.52 (0.48, 0.56) | 0.00 | 7.1 |
| 6 | 6 | Nap Index Z-Score | 25th | Random | 108 | 1544 | 0.52 (0.48, 0.56) | 0.00 | 7.1 |
| 6 | 6 | Nap Index Z-Score | 50th | Fixed | 113 | 1544 | 0.55 (0.52, 0.58) | 0.02 | 4.9 |
| 6 | 6 | Nap Index Z-Score | 50th | Random | 113 | 1544 | 0.55 (0.52, 0.58) | 0.02 | 4.9 |
| 6 | 6 | Nap Index Z-Score | 75th | Fixed | 111 | 1544 | 0.50 (0.47, 0.53) | 0.00 | 4.9 |
| 6 | 6 | Nap Index Z-Score | 75th | Random | 111 | 1544 | 0.50 (0.47, 0.53) | 0.00 | 4.9 |
| 6 | 6 | Sleep Index Z-Score | 25th | Fixed | 112 | 1522 | 0.50 (0.46, 0.53) | 0.00 | 8.5 |
| 6 | 6 | Sleep Index Z-Score | 25th | Random | 112 | 1522 | 0.50 (0.46, 0.53) | 0.00 | 8.5 |
| 6 | 6 | Sleep Index Z-Score | 50th | Fixed | 112 | 1522 | 0.49 (0.46, 0.52) | 0.00 | 7.1 |
| 6 | 6 | Sleep Index Z-Score | 50th | Random | 112 | 1522 | 0.49 (0.46, 0.52) | 0.00 | 7.1 |
| 6 | 6 | Sleep Index Z-Score | 75th | Fixed | 113 | 1522 | 0.49 (0.45, 0.52) | 0.00 | 4.2 |
| 6 | 6 | Sleep Index Z-Score | 75th | Random | 113 | 1522 | 0.49 (0.45, 0.52) | 0.00 | 4.2 |
| 6 | 12 | Nap Index Z-Score | 25th | Fixed | 111 | 1453 | 0.52 (0.49, 0.56) | 0.69 | 7.1 |
| 6 | 12 | Nap Index Z-Score | 25th | Random | 111 | 1453 | 0.53 (0.47, 0.59) | 0.69 | 7.1 |
| 6 | 12 | Nap Index Z-Score | 50th | Fixed | 114 | 1453 | 0.49 (0.46, 0.52) | 0.51 | 9.9 |
| 6 | 12 | Nap Index Z-Score | 50th | Random | 114 | 1453 | 0.49 (0.45, 0.54) | 0.51 | 9.9 |
| 6 | 12 | Nap Index Z-Score | 75th | Fixed | 113 | 1453 | 0.55 (0.52, 0.58) | 0.00 | 8.5 |
| 6 | 12 | Nap Index Z-Score | 75th | Random | 113 | 1453 | 0.55 (0.52, 0.58) | 0.00 | 8.5 |
| 6 | 12 | Sleep Index Z-Score | 25th | Fixed | 113 | 1455 | 0.50 (0.46, 0.54) | 0.52 | 7.8 |
| 6 | 12 | Sleep Index Z-Score | 25th | Random | 113 | 1455 | 0.50 (0.44, 0.55) | 0.52 | 7.8 |
| 6 | 12 | Sleep Index Z-Score | 50th | Fixed | 113 | 1455 | 0.46 (0.43, 0.49) | 0.09 | 7.8 |
| 6 | 12 | Sleep Index Z-Score | 50th | Random | 113 | 1455 | 0.46 (0.43, 0.50) | 0.09 | 7.8 |
| 6 | 12 | Sleep Index Z-Score | 75th | Fixed | 113 | 1455 | 0.45 (0.42, 0.48) | 0.00 | 8.5 |
| 6 | 12 | Sleep Index Z-Score | 75th | Random | 113 | 1455 | 0.45 (0.42, 0.48) | 0.00 | 8.5 |
| 6 | 12 | Walking alone |  | Fixed | 109 | 3982 | 0.53 (0.51, 0.55) | 0.00 | 3.1 |
| 6 | 12 | Walking alone |  | Random | 109 | 3982 | 0.53 (0.51, 0.55) | 0.00 | 3.1 |
| 6 | 18 | % time spent in MVPA Z-Score | 25th | Fixed | 104 | 1525 | 0.49 (0.45, 0.52) | 0.00 | 0.7 |
| 6 | 18 | % time spent in MVPA Z-Score | 25th | Random | 104 | 1525 | 0.49 (0.45, 0.52) | 0.00 | 0.7 |
| 6 | 18 | % time spent in MVPA Z-Score | 50th | Fixed | 104 | 1525 | 0.49 (0.46, 0.51) | 0.67 | 0.7 |
| 6 | 18 | % time spent in MVPA Z-Score | 50th | Random | 104 | 1525 | 0.49 (0.44, 0.55) | 0.67 | 0.7 |
| 6 | 18 | % time spent in MVPA Z-Score | 75th | Fixed | 103 | 1525 | 0.47 (0.44, 0.51) | 0.48 | 0.7 |
| 6 | 18 | % time spent in MVPA Z-Score | 75th | Random | 103 | 1525 | 0.48 (0.43, 0.52) | 0.48 | 0.7 |
| 6 | 18 | % time spent in sedentary behavior Z-Score | 25th | Fixed | 105 | 1525 | 0.51 (0.47, 0.54) | 0.00 | 0.7 |
| 6 | 18 | % time spent in sedentary behavior Z-Score | 25th | Random | 105 | 1525 | 0.51 (0.47, 0.54) | 0.00 | 0.7 |
| 6 | 18 | % time spent in sedentary behavior Z-Score | 50th | Fixed | 106 | 1525 | 0.48 (0.45, 0.51) | 0.00 | 3.5 |
| 6 | 18 | % time spent in sedentary behavior Z-Score | 50th | Random | 106 | 1525 | 0.48 (0.45, 0.51) | 0.00 | 3.5 |
| 6 | 18 | % time spent in sedentary behavior Z-Score | 75th | Fixed | 106 | 1525 | 0.49 (0.46, 0.53) | 0.00 | 1.4 |
| 6 | 18 | % time spent in sedentary behavior Z-Score | 75th | Random | 106 | 1525 | 0.49 (0.46, 0.53) | 0.00 | 1.4 |
| 6 | 18 | Fine Motor Z-Score | 25th | Fixed | 106 | 3701 | 0.49 (0.47, 0.52) | 0.00 | 4.7 |
| 6 | 18 | Fine Motor Z-Score | 25th | Random | 106 | 3701 | 0.49 (0.47, 0.52) | 0.00 | 4.7 |
| 6 | 18 | Fine Motor Z-Score | 50th | Fixed | 106 | 3701 | 0.48 (0.46, 0.50) | 0.54 | 3.5 |
| 6 | 18 | Fine Motor Z-Score | 50th | Random | 106 | 3701 | 0.48 (0.45, 0.51) | 0.54 | 3.5 |
| 6 | 18 | Fine Motor Z-Score | 75th | Fixed | 110 | 3701 | 0.48 (0.46, 0.50) | 0.00 | 3.6 |
| 6 | 18 | Fine Motor Z-Score | 75th | Random | 110 | 3701 | 0.48 (0.46, 0.50) | 0.00 | 3.6 |
| 6 | 18 | Gross Motor Z-Score | 25th | Fixed | 108 | 3621 | 0.52 (0.50, 0.54) | 0.36 | 4.9 |
| 6 | 18 | Gross Motor Z-Score | 25th | Random | 108 | 3621 | 0.52 (0.49, 0.55) | 0.36 | 4.9 |
| 6 | 18 | Gross Motor Z-Score | 50th | Fixed | 108 | 3621 | 0.51 (0.49, 0.53) | 0.00 | 4.2 |
| 6 | 18 | Gross Motor Z-Score | 50th | Random | 108 | 3621 | 0.51 (0.49, 0.53) | 0.00 | 4.2 |
| 6 | 18 | Gross Motor Z-Score | 75th | Fixed | 108 | 3621 | 0.51 (0.49, 0.53) | 0.00 | 4.4 |
| 6 | 18 | Gross Motor Z-Score | 75th | Random | 108 | 3621 | 0.51 (0.49, 0.53) | 0.00 | 4.4 |
| 6 | 18 | Language Z-Score | 25th | Fixed | 105 | 3780 | 0.52 (0.50, 0.54) | 0.41 | 6.6 |
| 6 | 18 | Language Z-Score | 25th | Random | 105 | 3780 | 0.51 (0.48, 0.55) | 0.41 | 6.6 |
| 6 | 18 | Language Z-Score | 50th | Fixed | 107 | 3780 | 0.52 (0.50, 0.54) | 0.00 | 4.9 |
| 6 | 18 | Language Z-Score | 50th | Random | 107 | 3780 | 0.52 (0.50, 0.54) | 0.00 | 4.9 |
| 6 | 18 | Language Z-Score | 75th | Fixed | 110 | 3780 | 0.52 (0.50, 0.54) | 0.41 | 3.8 |
| 6 | 18 | Language Z-Score | 75th | Random | 110 | 3780 | 0.52 (0.49, 0.55) | 0.41 | 3.8 |
| 6 | 18 | Mean Vector Magnitude Z-Score | 25th | Fixed | 104 | 1525 | 0.52 (0.49, 0.55) | 0.00 | 0.7 |
| 6 | 18 | Mean Vector Magnitude Z-Score | 25th | Random | 104 | 1525 | 0.52 (0.49, 0.55) | 0.00 | 0.7 |
| 6 | 18 | Mean Vector Magnitude Z-Score | 50th | Fixed | 104 | 1525 | 0.50 (0.47, 0.53) | 0.00 | 3.5 |
| 6 | 18 | Mean Vector Magnitude Z-Score | 50th | Random | 104 | 1525 | 0.50 (0.47, 0.53) | 0.00 | 3.5 |
| 6 | 18 | Mean Vector Magnitude Z-Score | 75th | Fixed | 107 | 1525 | 0.51 (0.48, 0.55) | 0.00 | 1.4 |
| 6 | 18 | Mean Vector Magnitude Z-Score | 75th | Random | 107 | 1525 | 0.51 (0.48, 0.55) | 0.00 | 1.4 |
| 6 | 18 | Motor Z-Score | 25th | Fixed | 108 | 3616 | 0.51 (0.49, 0.53) | 0.33 | 5.3 |
| 6 | 18 | Motor Z-Score | 25th | Random | 108 | 3616 | 0.51 (0.48, 0.54) | 0.33 | 5.3 |
| 6 | 18 | Motor Z-Score | 50th | Fixed | 109 | 3616 | 0.50 (0.48, 0.52) | 0.00 | 2.9 |
| 6 | 18 | Motor Z-Score | 50th | Random | 109 | 3616 | 0.50 (0.48, 0.52) | 0.00 | 2.9 |
| 6 | 18 | Motor Z-Score | 75th | Fixed | 109 | 3616 | 0.49 (0.47, 0.52) | 0.22 | 3.0 |
| 6 | 18 | Motor Z-Score | 75th | Random | 109 | 3616 | 0.49 (0.46, 0.52) | 0.22 | 3.0 |
| 6 | 18 | Nap Index Z-Score | 25th | Fixed | 112 | 1456 | 0.51 (0.47, 0.55) | 0.87 | 9.2 |
| 6 | 18 | Nap Index Z-Score | 25th | Random | 112 | 1456 | 0.54 (0.41, 0.66) | 0.87 | 9.2 |
| 6 | 18 | Nap Index Z-Score | 50th | Fixed | 113 | 1456 | 0.47 (0.43, 0.50) | 0.72 | 5.7 |
| 6 | 18 | Nap Index Z-Score | 50th | Random | 113 | 1456 | 0.47 (0.41, 0.53) | 0.72 | 5.7 |
| 6 | 18 | Nap Index Z-Score | 75th | Fixed | 112 | 1456 | 0.52 (0.49, 0.55) | 0.00 | 5.7 |
| 6 | 18 | Nap Index Z-Score | 75th | Random | 112 | 1456 | 0.52 (0.49, 0.55) | 0.00 | 5.7 |
| 6 | 18 | Sleep Index Z-Score | 25th | Fixed | 112 | 1456 | 0.49 (0.46, 0.53) | 0.68 | 6.4 |
| 6 | 18 | Sleep Index Z-Score | 25th | Random | 112 | 1456 | 0.49 (0.42, 0.55) | 0.68 | 6.4 |
| 6 | 18 | Sleep Index Z-Score | 50th | Fixed | 112 | 1456 | 0.48 (0.45, 0.51) | 0.11 | 8.5 |
| 6 | 18 | Sleep Index Z-Score | 50th | Random | 112 | 1456 | 0.48 (0.45, 0.51) | 0.11 | 8.5 |
| 6 | 18 | Sleep Index Z-Score | 75th | Fixed | 113 | 1456 | 0.48 (0.45, 0.52) | 0.66 | 8.5 |
| 6 | 18 | Sleep Index Z-Score | 75th | Random | 113 | 1456 | 0.48 (0.42, 0.54) | 0.66 | 8.5 |
| 6 | 18 | Social-Emotional Z-Score | 25th | Fixed | 107 | 3771 | 0.50 (0.48, 0.52) | 0.00 | 4.2 |
| 6 | 18 | Social-Emotional Z-Score | 25th | Random | 107 | 3771 | 0.50 (0.48, 0.52) | 0.00 | 4.2 |
| 6 | 18 | Social-Emotional Z-Score | 50th | Fixed | 109 | 3771 | 0.50 (0.48, 0.51) | 0.00 | 5.1 |
| 6 | 18 | Social-Emotional Z-Score | 50th | Random | 109 | 3771 | 0.50 (0.48, 0.51) | 0.00 | 5.1 |
| 6 | 18 | Social-Emotional Z-Score | 75th | Fixed | 110 | 3771 | 0.49 (0.46, 0.51) | 0.28 | 4.5 |
| 6 | 18 | Social-Emotional Z-Score | 75th | Random | 110 | 3771 | 0.48 (0.42, 0.53) | 0.28 | 4.5 |
| 6 | 24 | Fine Motor Z-Score | 25th | Fixed | 106 | 1520 | 0.51 (0.48, 0.55) | 0.00 | 3.5 |
| 6 | 24 | Fine Motor Z-Score | 25th | Random | 106 | 1520 | 0.51 (0.48, 0.55) | 0.00 | 3.5 |
| 6 | 24 | Fine Motor Z-Score | 50th | Fixed | 109 | 1520 | 0.48 (0.45, 0.50) | 0.00 | 5.7 |
| 6 | 24 | Fine Motor Z-Score | 50th | Random | 109 | 1520 | 0.48 (0.45, 0.50) | 0.00 | 5.7 |
| 6 | 24 | Fine Motor Z-Score | 75th | Fixed | 109 | 1520 | 0.50 (0.47, 0.53) | 0.00 | 6.4 |
| 6 | 24 | Fine Motor Z-Score | 75th | Random | 109 | 1520 | 0.50 (0.47, 0.53) | 0.00 | 6.4 |
| 6 | 24 | Gross Motor Z-Score | 25th | Fixed | 107 | 1480 | 0.51 (0.48, 0.55) | 0.65 | 3.5 |
| 6 | 24 | Gross Motor Z-Score | 25th | Random | 107 | 1480 | 0.52 (0.46, 0.58) | 0.65 | 3.5 |
| 6 | 24 | Gross Motor Z-Score | 50th | Fixed | 108 | 1480 | 0.52 (0.48, 0.55) | 0.66 | 5.7 |
| 6 | 24 | Gross Motor Z-Score | 50th | Random | 108 | 1480 | 0.52 (0.46, 0.57) | 0.66 | 5.7 |
| 6 | 24 | Gross Motor Z-Score | 75th | Fixed | 108 | 1480 | 0.49 (0.46, 0.52) | 0.00 | 6.4 |
| 6 | 24 | Gross Motor Z-Score | 75th | Random | 108 | 1480 | 0.49 (0.46, 0.52) | 0.00 | 6.4 |
| 6 | 24 | Language Z-Score | 25th | Fixed | 107 | 1527 | 0.52 (0.49, 0.56) | 0.00 | 4.9 |
| 6 | 24 | Language Z-Score | 25th | Random | 107 | 1527 | 0.52 (0.49, 0.56) | 0.00 | 4.9 |
| 6 | 24 | Language Z-Score | 50th | Fixed | 107 | 1527 | 0.52 (0.49, 0.55) | 0.00 | 6.4 |
| 6 | 24 | Language Z-Score | 50th | Random | 107 | 1527 | 0.52 (0.49, 0.55) | 0.00 | 6.4 |
| 6 | 24 | Language Z-Score | 75th | Fixed | 110 | 1527 | 0.52 (0.48, 0.55) | 0.00 | 6.4 |
| 6 | 24 | Language Z-Score | 75th | Random | 110 | 1527 | 0.52 (0.48, 0.55) | 0.00 | 6.4 |
| 6 | 24 | Motor Z-Score | 25th | Fixed | 106 | 1475 | 0.51 (0.47, 0.55) | 0.00 | 6.4 |
| 6 | 24 | Motor Z-Score | 25th | Random | 106 | 1475 | 0.51 (0.47, 0.55) | 0.00 | 6.4 |
| 6 | 24 | Motor Z-Score | 50th | Fixed | 108 | 1475 | 0.50 (0.47, 0.53) | 0.00 | 5.7 |
| 6 | 24 | Motor Z-Score | 50th | Random | 108 | 1475 | 0.50 (0.47, 0.53) | 0.00 | 5.7 |
| 6 | 24 | Motor Z-Score | 75th | Fixed | 108 | 1475 | 0.49 (0.46, 0.52) | 0.00 | 5.7 |
| 6 | 24 | Motor Z-Score | 75th | Random | 108 | 1475 | 0.49 (0.46, 0.52) | 0.00 | 5.7 |
| 6 | 24 | Social-Emotional Z-Score | 25th | Fixed | 107 | 1529 | 0.53 (0.50, 0.56) | 0.00 | 7.1 |
| 6 | 24 | Social-Emotional Z-Score | 25th | Random | 107 | 1529 | 0.53 (0.50, 0.56) | 0.00 | 7.1 |
| 6 | 24 | Social-Emotional Z-Score | 50th | Fixed | 106 | 1529 | 0.52 (0.49, 0.55) | 0.00 | 7.1 |
| 6 | 24 | Social-Emotional Z-Score | 50th | Random | 106 | 1529 | 0.52 (0.49, 0.55) | 0.00 | 7.1 |
| 6 | 24 | Social-Emotional Z-Score | 75th | Fixed | 109 | 1529 | 0.52 (0.49, 0.56) | 0.58 | 6.4 |
| 6 | 24 | Social-Emotional Z-Score | 75th | Random | 109 | 1529 | 0.53 (0.47, 0.58) | 0.58 | 6.4 |
| 9 | 9 | Fine Motor Z-Score | 25th | Fixed | 100 | 783 | 0.52 (0.47, 0.57) | 0.00 | 4.9 |
| 9 | 9 | Fine Motor Z-Score | 25th | Random | 100 | 783 | 0.52 (0.47, 0.57) | 0.00 | 4.9 |
| 9 | 9 | Fine Motor Z-Score | 50th | Fixed | 99 | 783 | 0.52 (0.47, 0.56) | 0.00 | 2.1 |
| 9 | 9 | Fine Motor Z-Score | 50th | Random | 99 | 783 | 0.52 (0.47, 0.56) | 0.00 | 2.1 |
| 9 | 9 | Fine Motor Z-Score | 75th | Fixed | 103 | 783 | 0.53 (0.48, 0.57) | 0.00 | 2.8 |
| 9 | 9 | Fine Motor Z-Score | 75th | Random | 103 | 783 | 0.53 (0.48, 0.57) | 0.00 | 2.8 |
| 9 | 9 | Gross Motor Z-Score | 25th | Fixed | 102 | 793 | 0.47 (0.42, 0.52) | 0.76 | 3.5 |
| 9 | 9 | Gross Motor Z-Score | 25th | Random | 102 | 793 | 0.47 (0.37, 0.58) | 0.76 | 3.5 |
| 9 | 9 | Gross Motor Z-Score | 50th | Fixed | 100 | 793 | 0.49 (0.45, 0.53) | 0.33 | 0.0 |
| 9 | 9 | Gross Motor Z-Score | 50th | Random | 100 | 793 | 0.49 (0.44, 0.54) | 0.33 | 0.0 |
| 9 | 9 | Gross Motor Z-Score | 75th | Fixed | 101 | 793 | 0.51 (0.46, 0.55) | 0.59 | 0.7 |
| 9 | 9 | Gross Motor Z-Score | 75th | Random | 101 | 793 | 0.51 (0.44, 0.58) | 0.59 | 0.7 |
| 9 | 9 | Language Z-Score | 25th | Fixed | 104 | 797 | 0.44 (0.39, 0.49) | 0.88 | 1.4 |
| 9 | 9 | Language Z-Score | 25th | Random | 104 | 797 | 0.45 (0.30, 0.60) | 0.88 | 1.4 |
| 9 | 9 | Language Z-Score | 50th | Fixed | 105 | 797 | 0.48 (0.43, 0.52) | 0.93 | 0.7 |
| 9 | 9 | Language Z-Score | 50th | Random | 105 | 797 | 0.48 (0.31, 0.65) | 0.93 | 0.7 |
| 9 | 9 | Language Z-Score | 75th | Fixed | 103 | 797 | 0.48 (0.43, 0.53) | 0.88 | 0.0 |
| 9 | 9 | Language Z-Score | 75th | Random | 103 | 797 | 0.48 (0.35, 0.62) | 0.88 | 0.0 |
| 9 | 9 | Motor Z-Score | 25th | Fixed | 102 | 779 | 0.49 (0.44, 0.54) | 0.62 | 4.2 |
| 9 | 9 | Motor Z-Score | 25th | Random | 102 | 779 | 0.49 (0.40, 0.58) | 0.62 | 4.2 |
| 9 | 9 | Motor Z-Score | 50th | Fixed | 100 | 779 | 0.49 (0.44, 0.53) | 0.45 | 0.0 |
| 9 | 9 | Motor Z-Score | 50th | Random | 100 | 779 | 0.49 (0.43, 0.54) | 0.45 | 0.0 |
| 9 | 9 | Motor Z-Score | 75th | Fixed | 100 | 779 | 0.50 (0.45, 0.55) | 0.13 | 2.8 |
| 9 | 9 | Motor Z-Score | 75th | Random | 100 | 779 | 0.50 (0.45, 0.55) | 0.13 | 2.8 |
| 9 | 9 | Social-Emotional Z-Score | 25th | Fixed | 104 | 797 | 0.43 (0.38, 0.48) | 0.82 | 2.1 |
| 9 | 9 | Social-Emotional Z-Score | 25th | Random | 104 | 797 | 0.44 (0.32, 0.55) | 0.82 | 2.1 |
| 9 | 9 | Social-Emotional Z-Score | 50th | Fixed | 105 | 797 | 0.46 (0.42, 0.51) | 0.89 | 0.7 |
| 9 | 9 | Social-Emotional Z-Score | 50th | Random | 105 | 797 | 0.46 (0.33, 0.60) | 0.89 | 0.7 |
| 9 | 9 | Social-Emotional Z-Score | 75th | Fixed | 100 | 797 | 0.47 (0.42, 0.52) | 0.83 | 2.1 |
| 9 | 9 | Social-Emotional Z-Score | 75th | Random | 100 | 797 | 0.48 (0.35, 0.61) | 0.83 | 2.1 |
| 12 | 12 | Fine Motor Z-Score | 25th | Fixed | 104 | 755 | 0.46 (0.41, 0.51) | 0.28 | 2.8 |
| 12 | 12 | Fine Motor Z-Score | 25th | Random | 104 | 755 | 0.46 (0.40, 0.52) | 0.28 | 2.8 |
| 12 | 12 | Fine Motor Z-Score | 50th | Fixed | 98 | 755 | 0.48 (0.44, 0.52) | 0.00 | 4.9 |
| 12 | 12 | Fine Motor Z-Score | 50th | Random | 98 | 755 | 0.48 (0.44, 0.52) | 0.00 | 4.9 |
| 12 | 12 | Fine Motor Z-Score | 75th | Fixed | 102 | 755 | 0.51 (0.46, 0.56) | 0.00 | 1.4 |
| 12 | 12 | Fine Motor Z-Score | 75th | Random | 102 | 755 | 0.51 (0.46, 0.56) | 0.00 | 1.4 |
| 12 | 12 | Gross Motor Z-Score | 25th | Fixed | 104 | 772 | 0.53 (0.48, 0.58) | 0.00 | 0.0 |
| 12 | 12 | Gross Motor Z-Score | 25th | Random | 104 | 772 | 0.53 (0.48, 0.58) | 0.00 | 0.0 |
| 12 | 12 | Gross Motor Z-Score | 50th | Fixed | 104 | 772 | 0.52 (0.48, 0.55) | 0.00 | 0.7 |
| 12 | 12 | Gross Motor Z-Score | 50th | Random | 104 | 772 | 0.52 (0.48, 0.55) | 0.00 | 0.7 |
| 12 | 12 | Gross Motor Z-Score | 75th | Fixed | 105 | 772 | 0.54 (0.49, 0.59) | 0.00 | 2.8 |
| 12 | 12 | Gross Motor Z-Score | 75th | Random | 105 | 772 | 0.54 (0.49, 0.59) | 0.00 | 2.8 |
| 12 | 12 | Language Z-Score | 25th | Fixed | 103 | 770 | 0.50 (0.45, 0.55) | 0.53 | 2.8 |
| 12 | 12 | Language Z-Score | 25th | Random | 103 | 770 | 0.50 (0.43, 0.57) | 0.53 | 2.8 |
| 12 | 12 | Language Z-Score | 50th | Fixed | 100 | 770 | 0.50 (0.46, 0.54) | 0.80 | 2.8 |
| 12 | 12 | Language Z-Score | 50th | Random | 100 | 770 | 0.49 (0.41, 0.58) | 0.80 | 2.8 |
| 12 | 12 | Language Z-Score | 75th | Fixed | 100 | 770 | 0.49 (0.44, 0.53) | 0.81 | 2.8 |
| 12 | 12 | Language Z-Score | 75th | Random | 100 | 770 | 0.49 (0.38, 0.60) | 0.81 | 2.8 |
| 12 | 12 | Motor Z-Score | 25th | Fixed | 104 | 752 | 0.48 (0.43, 0.53) | 0.00 | 2.8 |
| 12 | 12 | Motor Z-Score | 25th | Random | 104 | 752 | 0.48 (0.43, 0.53) | 0.00 | 2.8 |
| 12 | 12 | Motor Z-Score | 50th | Fixed | 105 | 752 | 0.50 (0.46, 0.55) | 0.00 | 0.7 |
| 12 | 12 | Motor Z-Score | 50th | Random | 105 | 752 | 0.50 (0.46, 0.55) | 0.00 | 0.7 |
| 12 | 12 | Motor Z-Score | 75th | Fixed | 105 | 752 | 0.56 (0.51, 0.60) | 0.00 | 0.7 |
| 12 | 12 | Motor Z-Score | 75th | Random | 105 | 752 | 0.56 (0.51, 0.60) | 0.00 | 0.7 |
| 12 | 12 | Social-Emotional Z-Score | 25th | Fixed | 103 | 770 | 0.47 (0.42, 0.52) | 0.00 | 2.1 |
| 12 | 12 | Social-Emotional Z-Score | 25th | Random | 103 | 770 | 0.47 (0.42, 0.52) | 0.00 | 2.1 |
| 12 | 12 | Social-Emotional Z-Score | 50th | Fixed | 100 | 770 | 0.49 (0.45, 0.53) | 0.73 | 2.1 |
| 12 | 12 | Social-Emotional Z-Score | 50th | Random | 100 | 770 | 0.49 (0.41, 0.57) | 0.73 | 2.1 |
| 12 | 12 | Social-Emotional Z-Score | 75th | Fixed | 100 | 770 | 0.49 (0.44, 0.53) | 0.79 | 5.7 |
| 12 | 12 | Social-Emotional Z-Score | 75th | Random | 100 | 770 | 0.49 (0.38, 0.59) | 0.79 | 5.7 |
| 12 | 12 | Walking alone |  | Fixed | 106 | 832 | 0.55 (0.51, 0.59) | 0.00 | 5.0 |
| 12 | 12 | Walking alone |  | Random | 106 | 832 | 0.55 (0.51, 0.59) | 0.00 | 5.0 |
| 12 | 24 | Fine Motor Z-Score | 25th | Fixed | 109 | 865 | 0.48 (0.44, 0.53) | 0.62 | 5.7 |
| 12 | 24 | Fine Motor Z-Score | 25th | Random | 109 | 865 | 0.47 (0.39, 0.55) | 0.62 | 5.7 |
| 12 | 24 | Fine Motor Z-Score | 50th | Fixed | 114 | 865 | 0.51 (0.47, 0.55) | 0.00 | 7.1 |
| 12 | 24 | Fine Motor Z-Score | 50th | Random | 114 | 865 | 0.51 (0.47, 0.55) | 0.00 | 7.1 |
| 12 | 24 | Fine Motor Z-Score | 75th | Fixed | 111 | 865 | 0.49 (0.45, 0.54) | 0.00 | 6.4 |
| 12 | 24 | Fine Motor Z-Score | 75th | Random | 111 | 865 | 0.49 (0.45, 0.54) | 0.00 | 6.4 |
| 12 | 24 | Gross Motor Z-Score | 25th | Fixed | 109 | 865 | 0.47 (0.43, 0.52) | 0.67 | 8.5 |
| 12 | 24 | Gross Motor Z-Score | 25th | Random | 109 | 865 | 0.46 (0.38, 0.54) | 0.67 | 8.5 |
| 12 | 24 | Gross Motor Z-Score | 50th | Fixed | 111 | 865 | 0.49 (0.46, 0.53) | 0.00 | 7.1 |
| 12 | 24 | Gross Motor Z-Score | 50th | Random | 111 | 865 | 0.49 (0.46, 0.53) | 0.00 | 7.1 |
| 12 | 24 | Gross Motor Z-Score | 75th | Fixed | 114 | 865 | 0.51 (0.46, 0.55) | 0.00 | 7.1 |
| 12 | 24 | Gross Motor Z-Score | 75th | Random | 114 | 865 | 0.51 (0.46, 0.55) | 0.00 | 7.1 |
| 12 | 24 | Language Z-Score | 25th | Fixed | 111 | 855 | 0.51 (0.47, 0.55) | 0.56 | 7.1 |
| 12 | 24 | Language Z-Score | 25th | Random | 111 | 855 | 0.50 (0.42, 0.57) | 0.56 | 7.1 |
| 12 | 24 | Language Z-Score | 50th | Fixed | 111 | 855 | 0.55 (0.51, 0.59) | 0.00 | 7.1 |
| 12 | 24 | Language Z-Score | 50th | Random | 111 | 855 | 0.55 (0.51, 0.59) | 0.00 | 7.1 |
| 12 | 24 | Language Z-Score | 75th | Fixed | 111 | 855 | 0.55 (0.50, 0.59) | 0.62 | 6.4 |
| 12 | 24 | Language Z-Score | 75th | Random | 111 | 855 | 0.56 (0.48, 0.64) | 0.62 | 6.4 |
| 12 | 24 | Motor Z-Score | 25th | Fixed | 107 | 865 | 0.48 (0.44, 0.53) | 0.16 | 7.1 |
| 12 | 24 | Motor Z-Score | 25th | Random | 107 | 865 | 0.48 (0.43, 0.53) | 0.16 | 7.1 |
| 12 | 24 | Motor Z-Score | 50th | Fixed | 114 | 865 | 0.49 (0.45, 0.53) | 0.00 | 7.1 |
| 12 | 24 | Motor Z-Score | 50th | Random | 114 | 865 | 0.49 (0.45, 0.53) | 0.00 | 7.1 |
| 12 | 24 | Motor Z-Score | 75th | Fixed | 110 | 865 | 0.52 (0.47, 0.56) | 0.52 | 7.1 |
| 12 | 24 | Motor Z-Score | 75th | Random | 110 | 865 | 0.51 (0.43, 0.58) | 0.52 | 7.1 |
| 12 | 24 | Social-Emotional Z-Score | 25th | Fixed | 109 | 865 | 0.49 (0.44, 0.54) | 0.65 | 7.8 |
| 12 | 24 | Social-Emotional Z-Score | 25th | Random | 109 | 865 | 0.47 (0.39, 0.56) | 0.65 | 7.8 |
| 12 | 24 | Social-Emotional Z-Score | 50th | Fixed | 110 | 865 | 0.53 (0.49, 0.57) | 0.00 | 7.8 |
| 12 | 24 | Social-Emotional Z-Score | 50th | Random | 110 | 865 | 0.53 (0.49, 0.57) | 0.00 | 7.8 |
| 12 | 24 | Social-Emotional Z-Score | 75th | Fixed | 109 | 865 | 0.56 (0.51, 0.60) | 0.32 | 7.1 |
| 12 | 24 | Social-Emotional Z-Score | 75th | Random | 109 | 865 | 0.55 (0.49, 0.61) | 0.32 | 7.1 |
| 15 | 15 | Fine Motor Z-Score | 25th | Fixed | 104 | 528 | 0.51 (0.45, 0.56) | 0.00 | 9.2 |
| 15 | 15 | Fine Motor Z-Score | 25th | Random | 104 | 528 | 0.51 (0.45, 0.56) | 0.00 | 9.2 |
| 15 | 15 | Fine Motor Z-Score | 50th | Fixed | 105 | 528 | 0.50 (0.45, 0.55) | 0.53 | 7.1 |
| 15 | 15 | Fine Motor Z-Score | 50th | Random | 105 | 528 | 0.51 (0.43, 0.59) | 0.53 | 7.1 |
| 15 | 15 | Fine Motor Z-Score | 75th | Fixed | 104 | 528 | 0.51 (0.44, 0.58) | 0.47 | 6.4 |
| 15 | 15 | Fine Motor Z-Score | 75th | Random | 104 | 528 | 0.52 (0.42, 0.61) | 0.47 | 6.4 |
| 15 | 15 | Gross Motor Z-Score | 25th | Fixed | 104 | 541 | 0.55 (0.50, 0.61) | 0.00 | 4.9 |
| 15 | 15 | Gross Motor Z-Score | 25th | Random | 104 | 541 | 0.55 (0.50, 0.61) | 0.00 | 4.9 |
| 15 | 15 | Gross Motor Z-Score | 50th | Fixed | 104 | 541 | 0.58 (0.53, 0.63) | 0.00 | 3.5 |
| 15 | 15 | Gross Motor Z-Score | 50th | Random | 104 | 541 | 0.58 (0.53, 0.63) | 0.00 | 3.5 |
| 15 | 15 | Gross Motor Z-Score | 75th | Fixed | 104 | 541 | 0.56 (0.51, 0.62) | 0.00 | 4.9 |
| 15 | 15 | Gross Motor Z-Score | 75th | Random | 104 | 541 | 0.56 (0.51, 0.62) | 0.00 | 4.9 |
| 15 | 15 | Language Z-Score | 25th | Fixed | 104 | 539 | 0.51 (0.45, 0.56) | 0.81 | 5.7 |
| 15 | 15 | Language Z-Score | 25th | Random | 104 | 539 | 0.52 (0.38, 0.66) | 0.81 | 5.7 |
| 15 | 15 | Language Z-Score | 50th | Fixed | 101 | 539 | 0.49 (0.45, 0.53) | 0.84 | 8.5 |
| 15 | 15 | Language Z-Score | 50th | Random | 101 | 539 | 0.51 (0.40, 0.63) | 0.84 | 8.5 |
| 15 | 15 | Language Z-Score | 75th | Fixed | 105 | 539 | 0.56 (0.52, 0.61) | 0.83 | 6.4 |
| 15 | 15 | Language Z-Score | 75th | Random | 105 | 539 | 0.59 (0.46, 0.71) | 0.83 | 6.4 |
| 15 | 15 | Motor Z-Score | 25th | Fixed | 104 | 527 | 0.55 (0.49, 0.60) | 0.00 | 6.4 |
| 15 | 15 | Motor Z-Score | 25th | Random | 104 | 527 | 0.55 (0.49, 0.60) | 0.00 | 6.4 |
| 15 | 15 | Motor Z-Score | 50th | Fixed | 104 | 527 | 0.55 (0.50, 0.60) | 0.00 | 4.9 |
| 15 | 15 | Motor Z-Score | 50th | Random | 104 | 527 | 0.55 (0.50, 0.60) | 0.00 | 4.9 |
| 15 | 15 | Motor Z-Score | 75th | Fixed | 104 | 527 | 0.53 (0.47, 0.59) | 0.67 | 5.7 |
| 15 | 15 | Motor Z-Score | 75th | Random | 104 | 527 | 0.54 (0.42, 0.65) | 0.67 | 5.7 |
| 15 | 15 | Social-Emotional Z-Score | 25th | Fixed | 97 | 539 | 0.53 (0.47, 0.58) | 0.89 | 4.9 |
| 15 | 15 | Social-Emotional Z-Score | 25th | Random | 97 | 539 | 0.54 (0.37, 0.71) | 0.89 | 4.9 |
| 15 | 15 | Social-Emotional Z-Score | 50th | Fixed | 98 | 539 | 0.50 (0.46, 0.54) | 0.92 | 7.1 |
| 15 | 15 | Social-Emotional Z-Score | 50th | Random | 98 | 539 | 0.54 (0.37, 0.72) | 0.92 | 7.1 |
| 15 | 15 | Social-Emotional Z-Score | 75th | Fixed | 104 | 539 | 0.56 (0.51, 0.61) | 0.79 | 9.2 |
| 15 | 15 | Social-Emotional Z-Score | 75th | Random | 104 | 539 | 0.58 (0.46, 0.71) | 0.79 | 9.2 |
| 18 | 18 | % time spent in MVPA Z-Score | 25th | Fixed | 110 | 955 | 0.54 (0.50, 0.58) | 0.17 | 4.9 |
| 18 | 18 | % time spent in MVPA Z-Score | 25th | Random | 110 | 955 | 0.54 (0.49, 0.59) | 0.17 | 4.9 |
| 18 | 18 | % time spent in MVPA Z-Score | 50th | Fixed | 107 | 955 | 0.54 (0.50, 0.57) | 0.00 | 3.5 |
| 18 | 18 | % time spent in MVPA Z-Score | 50th | Random | 107 | 955 | 0.54 (0.50, 0.57) | 0.00 | 3.5 |
| 18 | 18 | % time spent in MVPA Z-Score | 75th | Fixed | 111 | 955 | 0.56 (0.52, 0.60) | 0.49 | 0.7 |
| 18 | 18 | % time spent in MVPA Z-Score | 75th | Random | 111 | 955 | 0.56 (0.51, 0.62) | 0.49 | 0.7 |
| 18 | 18 | % time spent in sedentary behavior Z-Score | 25th | Fixed | 108 | 955 | 0.41 (0.37, 0.45) | 0.66 | 1.4 |
| 18 | 18 | % time spent in sedentary behavior Z-Score | 25th | Random | 108 | 955 | 0.40 (0.34, 0.47) | 0.66 | 1.4 |
| 18 | 18 | % time spent in sedentary behavior Z-Score | 50th | Fixed | 109 | 955 | 0.41 (0.37, 0.44) | 0.50 | 1.4 |
| 18 | 18 | % time spent in sedentary behavior Z-Score | 50th | Random | 109 | 955 | 0.41 (0.35, 0.46) | 0.50 | 1.4 |
| 18 | 18 | % time spent in sedentary behavior Z-Score | 75th | Fixed | 106 | 955 | 0.41 (0.37, 0.46) | 0.00 | 2.8 |
| 18 | 18 | % time spent in sedentary behavior Z-Score | 75th | Random | 106 | 955 | 0.41 (0.37, 0.46) | 0.00 | 2.8 |
| 18 | 18 | Fine Motor Z-Score | 25th | Fixed | 109 | 3753 | 0.53 (0.51, 0.55) | 0.73 | 4.8 |
| 18 | 18 | Fine Motor Z-Score | 25th | Random | 109 | 3753 | 0.54 (0.50, 0.58) | 0.73 | 4.8 |
| 18 | 18 | Fine Motor Z-Score | 50th | Fixed | 110 | 3753 | 0.52 (0.50, 0.54) | 0.63 | 5.1 |
| 18 | 18 | Fine Motor Z-Score | 50th | Random | 110 | 3753 | 0.53 (0.50, 0.56) | 0.63 | 5.1 |
| 18 | 18 | Fine Motor Z-Score | 75th | Fixed | 113 | 3753 | 0.53 (0.51, 0.55) | 0.41 | 4.9 |
| 18 | 18 | Fine Motor Z-Score | 75th | Random | 113 | 3753 | 0.53 (0.50, 0.56) | 0.41 | 4.9 |
| 18 | 18 | Gross Motor Z-Score | 25th | Fixed | 108 | 3684 | 0.54 (0.52, 0.56) | 0.47 | 5.7 |
| 18 | 18 | Gross Motor Z-Score | 25th | Random | 108 | 3684 | 0.55 (0.51, 0.58) | 0.47 | 5.7 |
| 18 | 18 | Gross Motor Z-Score | 50th | Fixed | 110 | 3684 | 0.53 (0.51, 0.55) | 0.14 | 5.1 |
| 18 | 18 | Gross Motor Z-Score | 50th | Random | 110 | 3684 | 0.53 (0.51, 0.55) | 0.14 | 5.1 |
| 18 | 18 | Gross Motor Z-Score | 75th | Fixed | 110 | 3684 | 0.52 (0.50, 0.54) | 0.03 | 5.3 |
| 18 | 18 | Gross Motor Z-Score | 75th | Random | 110 | 3684 | 0.52 (0.50, 0.54) | 0.03 | 5.3 |
| 18 | 18 | Language Z-Score | 25th | Fixed | 103 | 4963 | 0.56 (0.54, 0.58) | 0.31 | 6.1 |
| 18 | 18 | Language Z-Score | 25th | Random | 103 | 4963 | 0.56 (0.53, 0.58) | 0.31 | 6.1 |
| 18 | 18 | Language Z-Score | 50th | Fixed | 109 | 4963 | 0.55 (0.53, 0.56) | 0.65 | 6.8 |
| 18 | 18 | Language Z-Score | 50th | Random | 109 | 4963 | 0.55 (0.52, 0.58) | 0.65 | 6.8 |
| 18 | 18 | Language Z-Score | 75th | Fixed | 110 | 4963 | 0.55 (0.53, 0.57) | 0.36 | 6.6 |
| 18 | 18 | Language Z-Score | 75th | Random | 110 | 4963 | 0.55 (0.53, 0.58) | 0.36 | 6.6 |
| 18 | 18 | Mean Vector Magnitude Z-Score | 25th | Fixed | 102 | 954 | 0.60 (0.56, 0.65) | 0.00 | 6.4 |
| 18 | 18 | Mean Vector Magnitude Z-Score | 25th | Random | 102 | 954 | 0.60 (0.56, 0.65) | 0.00 | 6.4 |
| 18 | 18 | Mean Vector Magnitude Z-Score | 50th | Fixed | 106 | 954 | 0.59 (0.55, 0.63) | 0.44 | 4.9 |
| 18 | 18 | Mean Vector Magnitude Z-Score | 50th | Random | 106 | 954 | 0.59 (0.54, 0.64) | 0.44 | 4.9 |
| 18 | 18 | Mean Vector Magnitude Z-Score | 75th | Fixed | 109 | 954 | 0.59 (0.55, 0.63) | 0.12 | 6.4 |
| 18 | 18 | Mean Vector Magnitude Z-Score | 75th | Random | 109 | 954 | 0.59 (0.55, 0.64) | 0.12 | 6.4 |
| 18 | 18 | Motor Z-Score | 25th | Fixed | 104 | 4781 | 0.56 (0.54, 0.58) | 0.61 | 6.9 |
| 18 | 18 | Motor Z-Score | 25th | Random | 104 | 4781 | 0.56 (0.53, 0.58) | 0.61 | 6.9 |
| 18 | 18 | Motor Z-Score | 50th | Fixed | 108 | 4781 | 0.55 (0.53, 0.57) | 0.75 | 7.8 |
| 18 | 18 | Motor Z-Score | 50th | Random | 108 | 4781 | 0.54 (0.52, 0.57) | 0.75 | 7.8 |
| 18 | 18 | Motor Z-Score | 75th | Fixed | 109 | 4781 | 0.54 (0.52, 0.55) | 0.45 | 7.7 |
| 18 | 18 | Motor Z-Score | 75th | Random | 109 | 4781 | 0.54 (0.51, 0.56) | 0.45 | 7.7 |
| 18 | 18 | Nap Index Z-Score | 25th | Fixed | 112 | 1597 | 0.48 (0.44, 0.51) | 0.00 | 2.8 |
| 18 | 18 | Nap Index Z-Score | 25th | Random | 112 | 1597 | 0.48 (0.44, 0.51) | 0.00 | 2.8 |
| 18 | 18 | Nap Index Z-Score | 50th | Fixed | 112 | 1597 | 0.46 (0.44, 0.49) | 0.00 | 2.1 |
| 18 | 18 | Nap Index Z-Score | 50th | Random | 112 | 1597 | 0.46 (0.44, 0.49) | 0.00 | 2.1 |
| 18 | 18 | Nap Index Z-Score | 75th | Fixed | 112 | 1597 | 0.50 (0.47, 0.53) | 0.07 | 2.1 |
| 18 | 18 | Nap Index Z-Score | 75th | Random | 112 | 1597 | 0.50 (0.47, 0.53) | 0.07 | 2.1 |
| 18 | 18 | Sleep Index Z-Score | 25th | Fixed | 110 | 1597 | 0.48 (0.45, 0.52) | 0.00 | 1.4 |
| 18 | 18 | Sleep Index Z-Score | 25th | Random | 110 | 1597 | 0.48 (0.45, 0.52) | 0.00 | 1.4 |
| 18 | 18 | Sleep Index Z-Score | 50th | Fixed | 110 | 1597 | 0.49 (0.47, 0.52) | 0.40 | 3.5 |
| 18 | 18 | Sleep Index Z-Score | 50th | Random | 110 | 1597 | 0.50 (0.46, 0.53) | 0.40 | 3.5 |
| 18 | 18 | Sleep Index Z-Score | 75th | Fixed | 114 | 1597 | 0.48 (0.45, 0.51) | 0.55 | 2.1 |
| 18 | 18 | Sleep Index Z-Score | 75th | Random | 114 | 1597 | 0.48 (0.44, 0.53) | 0.55 | 2.1 |
| 18 | 18 | Social-Emotional Z-Score | 25th | Fixed | 104 | 4952 | 0.52 (0.50, 0.54) | 0.19 | 6.5 |
| 18 | 18 | Social-Emotional Z-Score | 25th | Random | 104 | 4952 | 0.52 (0.50, 0.54) | 0.19 | 6.5 |
| 18 | 18 | Social-Emotional Z-Score | 50th | Fixed | 110 | 4952 | 0.52 (0.51, 0.54) | 0.00 | 7.6 |
| 18 | 18 | Social-Emotional Z-Score | 50th | Random | 110 | 4952 | 0.52 (0.51, 0.54) | 0.00 | 7.6 |
| 18 | 18 | Social-Emotional Z-Score | 75th | Fixed | 109 | 4952 | 0.53 (0.51, 0.55) | 0.08 | 6.5 |
| 18 | 18 | Social-Emotional Z-Score | 75th | Random | 109 | 4952 | 0.53 (0.51, 0.55) | 0.08 | 6.5 |
| 18 | 24 | Fine Motor Z-Score | 25th | Fixed | 117 | 2870 | 0.52 (0.49, 0.54) | 0.00 | 0.7 |
| 18 | 24 | Fine Motor Z-Score | 25th | Random | 117 | 2870 | 0.52 (0.49, 0.54) | 0.00 | 0.7 |
| 18 | 24 | Fine Motor Z-Score | 50th | Fixed | 116 | 2870 | 0.51 (0.49, 0.53) | 0.00 | 1.4 |
| 18 | 24 | Fine Motor Z-Score | 50th | Random | 116 | 2870 | 0.51 (0.49, 0.53) | 0.00 | 1.4 |
| 18 | 24 | Fine Motor Z-Score | 75th | Fixed | 116 | 2870 | 0.52 (0.50, 0.55) | 0.28 | 0.0 |
| 18 | 24 | Fine Motor Z-Score | 75th | Random | 116 | 2870 | 0.52 (0.49, 0.55) | 0.28 | 0.0 |
| 18 | 24 | Gross Motor Z-Score | 25th | Fixed | 116 | 2829 | 0.54 (0.51, 0.56) | 0.00 | 2.1 |
| 18 | 24 | Gross Motor Z-Score | 25th | Random | 116 | 2829 | 0.54 (0.51, 0.56) | 0.00 | 2.1 |
| 18 | 24 | Gross Motor Z-Score | 50th | Fixed | 116 | 2829 | 0.51 (0.49, 0.53) | 0.00 | 0.7 |
| 18 | 24 | Gross Motor Z-Score | 50th | Random | 116 | 2829 | 0.51 (0.49, 0.53) | 0.00 | 0.7 |
| 18 | 24 | Gross Motor Z-Score | 75th | Fixed | 117 | 2829 | 0.52 (0.49, 0.54) | 0.24 | 1.4 |
| 18 | 24 | Gross Motor Z-Score | 75th | Random | 117 | 2829 | 0.52 (0.49, 0.54) | 0.24 | 1.4 |
| 18 | 24 | Language Z-Score | 25th | Fixed | 116 | 2839 | 0.52 (0.50, 0.55) | 0.00 | 0.0 |
| 18 | 24 | Language Z-Score | 25th | Random | 116 | 2839 | 0.52 (0.50, 0.55) | 0.00 | 0.0 |
| 18 | 24 | Language Z-Score | 50th | Fixed | 116 | 2839 | 0.52 (0.50, 0.54) | 0.00 | 0.0 |
| 18 | 24 | Language Z-Score | 50th | Random | 116 | 2839 | 0.52 (0.50, 0.54) | 0.00 | 0.0 |
| 18 | 24 | Language Z-Score | 75th | Fixed | 116 | 2839 | 0.50 (0.48, 0.52) | 0.00 | 0.0 |
| 18 | 24 | Language Z-Score | 75th | Random | 116 | 2839 | 0.50 (0.48, 0.52) | 0.00 | 0.0 |
| 18 | 24 | Motor Z-Score | 25th | Fixed | 116 | 2824 | 0.54 (0.52, 0.57) | 0.00 | 2.1 |
| 18 | 24 | Motor Z-Score | 25th | Random | 116 | 2824 | 0.54 (0.52, 0.57) | 0.00 | 2.1 |
| 18 | 24 | Motor Z-Score | 50th | Fixed | 116 | 2824 | 0.52 (0.50, 0.54) | 0.00 | 1.4 |
| 18 | 24 | Motor Z-Score | 50th | Random | 116 | 2824 | 0.52 (0.50, 0.54) | 0.00 | 1.4 |
| 18 | 24 | Motor Z-Score | 75th | Fixed | 117 | 2824 | 0.51 (0.49, 0.54) | 0.00 | 0.0 |
| 18 | 24 | Motor Z-Score | 75th | Random | 117 | 2824 | 0.51 (0.49, 0.54) | 0.00 | 0.0 |
| 18 | 24 | Social-Emotional Z-Score | 25th | Fixed | 116 | 2879 | 0.52 (0.49, 0.54) | 0.00 | 2.1 |
| 18 | 24 | Social-Emotional Z-Score | 25th | Random | 116 | 2879 | 0.52 (0.49, 0.54) | 0.00 | 2.1 |
| 18 | 24 | Social-Emotional Z-Score | 50th | Fixed | 116 | 2879 | 0.49 (0.47, 0.51) | 0.00 | 3.5 |
| 18 | 24 | Social-Emotional Z-Score | 50th | Random | 116 | 2879 | 0.49 (0.47, 0.51) | 0.00 | 3.5 |
| 18 | 24 | Social-Emotional Z-Score | 75th | Fixed | 116 | 2879 | 0.49 (0.47, 0.52) | 0.00 | 3.5 |
| 18 | 24 | Social-Emotional Z-Score | 75th | Random | 116 | 2879 | 0.49 (0.47, 0.52) | 0.00 | 3.5 |
| 21 | 21 | Fine Motor Z-Score | 25th | Fixed | 105 | 901 | 0.54 (0.49, 0.58) | 0.44 | 4.2 |
| 21 | 21 | Fine Motor Z-Score | 25th | Random | 105 | 901 | 0.54 (0.48, 0.60) | 0.44 | 4.2 |
| 21 | 21 | Fine Motor Z-Score | 50th | Fixed | 106 | 901 | 0.50 (0.46, 0.55) | 0.49 | 7.8 |
| 21 | 21 | Fine Motor Z-Score | 50th | Random | 106 | 901 | 0.51 (0.45, 0.57) | 0.49 | 7.8 |
| 21 | 21 | Fine Motor Z-Score | 75th | Fixed | 106 | 901 | 0.49 (0.45, 0.54) | 0.76 | 6.4 |
| 21 | 21 | Fine Motor Z-Score | 75th | Random | 106 | 901 | 0.50 (0.41, 0.59) | 0.76 | 6.4 |
| 21 | 21 | Gross Motor Z-Score | 25th | Fixed | 105 | 959 | 0.50 (0.46, 0.55) | 0.57 | 6.0 |
| 21 | 21 | Gross Motor Z-Score | 25th | Random | 105 | 959 | 0.53 (0.42, 0.64) | 0.57 | 6.0 |
| 21 | 21 | Gross Motor Z-Score | 50th | Fixed | 105 | 959 | 0.52 (0.48, 0.55) | 0.83 | 8.3 |
| 21 | 21 | Gross Motor Z-Score | 50th | Random | 105 | 959 | 0.57 (0.41, 0.74) | 0.83 | 8.3 |
| 21 | 21 | Gross Motor Z-Score | 75th | Fixed | 107 | 959 | 0.51 (0.47, 0.55) | 0.74 | 8.3 |
| 21 | 21 | Gross Motor Z-Score | 75th | Random | 107 | 959 | 0.55 (0.41, 0.70) | 0.74 | 8.3 |
| 21 | 21 | Language Z-Score | 25th | Fixed | 103 | 948 | 0.56 (0.52, 0.61) | 0.21 | 7.5 |
| 21 | 21 | Language Z-Score | 25th | Random | 103 | 948 | 0.56 (0.51, 0.61) | 0.21 | 7.5 |
| 21 | 21 | Language Z-Score | 50th | Fixed | 105 | 948 | 0.55 (0.52, 0.59) | 0.14 | 12.5 |
| 21 | 21 | Language Z-Score | 50th | Random | 105 | 948 | 0.56 (0.52, 0.60) | 0.14 | 12.5 |
| 21 | 21 | Language Z-Score | 75th | Fixed | 109 | 948 | 0.53 (0.49, 0.57) | 0.41 | 11.5 |
| 21 | 21 | Language Z-Score | 75th | Random | 109 | 948 | 0.53 (0.47, 0.60) | 0.41 | 11.5 |
| 21 | 21 | Motor Z-Score | 25th | Fixed | 105 | 942 | 0.56 (0.52, 0.61) | 0.40 | 6.0 |
| 21 | 21 | Motor Z-Score | 25th | Random | 105 | 942 | 0.57 (0.51, 0.64) | 0.40 | 6.0 |
| 21 | 21 | Motor Z-Score | 50th | Fixed | 106 | 942 | 0.52 (0.48, 0.56) | 0.85 | 8.9 |
| 21 | 21 | Motor Z-Score | 50th | Random | 106 | 942 | 0.58 (0.42, 0.74) | 0.85 | 8.9 |
| 21 | 21 | Motor Z-Score | 75th | Fixed | 109 | 942 | 0.53 (0.48, 0.57) | 0.77 | 8.3 |
| 21 | 21 | Motor Z-Score | 75th | Random | 109 | 942 | 0.57 (0.43, 0.71) | 0.77 | 8.3 |
| 21 | 21 | Social-Emotional Z-Score | 25th | Fixed | 105 | 948 | 0.56 (0.52, 0.61) | 0.75 | 9.0 |
| 21 | 21 | Social-Emotional Z-Score | 25th | Random | 105 | 948 | 0.59 (0.47, 0.70) | 0.75 | 9.0 |
| 21 | 21 | Social-Emotional Z-Score | 50th | Fixed | 105 | 948 | 0.54 (0.50, 0.57) | 0.42 | 8.7 |
| 21 | 21 | Social-Emotional Z-Score | 50th | Random | 105 | 948 | 0.54 (0.49, 0.59) | 0.42 | 8.7 |
| 21 | 21 | Social-Emotional Z-Score | 75th | Fixed | 112 | 948 | 0.53 (0.48, 0.58) | 0.53 | 7.5 |
| 21 | 21 | Social-Emotional Z-Score | 75th | Random | 112 | 948 | 0.54 (0.47, 0.61) | 0.53 | 7.5 |
| 21 | 24 | Gross Motor Z-Score | 25th | Fixed | 113 | 411 | 0.50 (0.44, 0.56) | 0.61 | 5.1 |
| 21 | 24 | Gross Motor Z-Score | 25th | Random | 113 | 411 | 0.48 (0.37, 0.58) | 0.61 | 5.1 |
| 21 | 24 | Gross Motor Z-Score | 50th | Fixed | 113 | 411 | 0.50 (0.45, 0.56) | 0.04 | 4.9 |
| 21 | 24 | Gross Motor Z-Score | 50th | Random | 113 | 411 | 0.50 (0.45, 0.56) | 0.04 | 4.9 |
| 21 | 24 | Gross Motor Z-Score | 75th | Fixed | 115 | 411 | 0.54 (0.48, 0.59) | 0.00 | 6.4 |
| 21 | 24 | Gross Motor Z-Score | 75th | Random | 115 | 411 | 0.54 (0.48, 0.59) | 0.00 | 6.4 |
| 21 | 24 | Language Z-Score | 25th | Fixed | 116 | 409 | 0.55 (0.49, 0.62) | 0.00 | 1.5 |
| 21 | 24 | Language Z-Score | 25th | Random | 116 | 409 | 0.55 (0.49, 0.62) | 0.00 | 1.5 |
| 21 | 24 | Language Z-Score | 50th | Fixed | 116 | 409 | 0.55 (0.51, 0.60) | 0.00 | 1.5 |
| 21 | 24 | Language Z-Score | 50th | Random | 116 | 409 | 0.55 (0.51, 0.60) | 0.00 | 1.5 |
| 21 | 24 | Language Z-Score | 75th | Fixed | 117 | 409 | 0.54 (0.48, 0.60) | 0.00 | 1.2 |
| 21 | 24 | Language Z-Score | 75th | Random | 117 | 409 | 0.54 (0.48, 0.60) | 0.00 | 1.2 |
| 21 | 24 | Motor Z-Score | 25th | Fixed | 113 | 411 | 0.49 (0.44, 0.55) | 0.68 | 5.1 |
| 21 | 24 | Motor Z-Score | 25th | Random | 113 | 411 | 0.47 (0.34, 0.59) | 0.68 | 5.1 |
| 21 | 24 | Motor Z-Score | 50th | Fixed | 116 | 411 | 0.52 (0.47, 0.57) | 0.00 | 4.9 |
| 21 | 24 | Motor Z-Score | 50th | Random | 116 | 411 | 0.52 (0.47, 0.57) | 0.00 | 4.9 |
| 21 | 24 | Motor Z-Score | 75th | Fixed | 115 | 411 | 0.53 (0.47, 0.59) | 0.00 | 6.4 |
| 21 | 24 | Motor Z-Score | 75th | Random | 115 | 411 | 0.53 (0.47, 0.59) | 0.00 | 6.4 |
| 21 | 24 | Social-Emotional Z-Score | 25th | Fixed | 114 | 411 | 0.48 (0.41, 0.54) | 0.00 | 1.2 |
| 21 | 24 | Social-Emotional Z-Score | 25th | Random | 114 | 411 | 0.48 (0.41, 0.54) | 0.00 | 1.2 |
| 21 | 24 | Social-Emotional Z-Score | 50th | Fixed | 115 | 411 | 0.49 (0.43, 0.54) | 0.00 | 4.0 |
| 21 | 24 | Social-Emotional Z-Score | 50th | Random | 115 | 411 | 0.49 (0.43, 0.54) | 0.00 | 4.0 |
| 21 | 24 | Social-Emotional Z-Score | 75th | Fixed | 118 | 411 | 0.53 (0.47, 0.60) | 0.56 | 3.6 |
| 21 | 24 | Social-Emotional Z-Score | 75th | Random | 118 | 411 | 0.53 (0.43, 0.63) | 0.56 | 3.6 |
| 24 | 24 | Fine Motor Z-Score | 25th | Fixed | 109 | 881 | 0.54 (0.49, 0.59) | 0.00 | 5.7 |
| 24 | 24 | Fine Motor Z-Score | 25th | Random | 109 | 881 | 0.54 (0.49, 0.59) | 0.00 | 5.7 |
| 24 | 24 | Fine Motor Z-Score | 50th | Fixed | 109 | 881 | 0.52 (0.49, 0.56) | 0.00 | 5.7 |
| 24 | 24 | Fine Motor Z-Score | 50th | Random | 109 | 881 | 0.52 (0.49, 0.56) | 0.00 | 5.7 |
| 24 | 24 | Fine Motor Z-Score | 75th | Fixed | 110 | 881 | 0.49 (0.44, 0.54) | 0.48 | 5.7 |
| 24 | 24 | Fine Motor Z-Score | 75th | Random | 110 | 881 | 0.48 (0.40, 0.56) | 0.48 | 5.7 |
| 24 | 24 | Gross Motor Z-Score | 25th | Fixed | 108 | 1086 | 0.53 (0.49, 0.57) | 0.00 | 10.8 |
| 24 | 24 | Gross Motor Z-Score | 25th | Random | 108 | 1086 | 0.53 (0.49, 0.57) | 0.00 | 10.8 |
| 24 | 24 | Gross Motor Z-Score | 50th | Fixed | 108 | 1086 | 0.52 (0.48, 0.55) | 0.42 | 9.9 |
| 24 | 24 | Gross Motor Z-Score | 50th | Random | 108 | 1086 | 0.51 (0.46, 0.56) | 0.42 | 9.9 |
| 24 | 24 | Gross Motor Z-Score | 75th | Fixed | 108 | 1086 | 0.50 (0.46, 0.54) | 0.00 | 7.5 |
| 24 | 24 | Gross Motor Z-Score | 75th | Random | 108 | 1086 | 0.50 (0.46, 0.54) | 0.00 | 7.5 |
| 24 | 24 | Language Z-Score | 25th | Fixed | 107 | 1091 | 0.54 (0.50, 0.58) | 0.40 | 11.6 |
| 24 | 24 | Language Z-Score | 25th | Random | 107 | 1091 | 0.53 (0.46, 0.60) | 0.40 | 11.6 |
| 24 | 24 | Language Z-Score | 50th | Fixed | 108 | 1091 | 0.54 (0.51, 0.58) | 0.00 | 9.0 |
| 24 | 24 | Language Z-Score | 50th | Random | 108 | 1091 | 0.54 (0.51, 0.58) | 0.00 | 9.0 |
| 24 | 24 | Language Z-Score | 75th | Fixed | 108 | 1091 | 0.54 (0.50, 0.58) | 0.00 | 8.6 |
| 24 | 24 | Language Z-Score | 75th | Random | 108 | 1091 | 0.54 (0.50, 0.58) | 0.00 | 8.6 |
| 24 | 24 | Motor Z-Score | 25th | Fixed | 108 | 1082 | 0.53 (0.49, 0.56) | 0.00 | 10.3 |
| 24 | 24 | Motor Z-Score | 25th | Random | 108 | 1082 | 0.53 (0.49, 0.56) | 0.00 | 10.3 |
| 24 | 24 | Motor Z-Score | 50th | Fixed | 108 | 1082 | 0.53 (0.50, 0.57) | 0.00 | 10.0 |
| 24 | 24 | Motor Z-Score | 50th | Random | 108 | 1082 | 0.53 (0.50, 0.57) | 0.00 | 10.0 |
| 24 | 24 | Motor Z-Score | 75th | Fixed | 107 | 1082 | 0.50 (0.46, 0.55) | 0.00 | 8.6 |
| 24 | 24 | Motor Z-Score | 75th | Random | 107 | 1082 | 0.50 (0.46, 0.55) | 0.00 | 8.6 |
| 24 | 24 | Social-Emotional Z-Score | 25th | Fixed | 107 | 1087 | 0.55 (0.51, 0.59) | 0.00 | 7.0 |
| 24 | 24 | Social-Emotional Z-Score | 25th | Random | 107 | 1087 | 0.55 (0.51, 0.59) | 0.00 | 7.0 |
| 24 | 24 | Social-Emotional Z-Score | 50th | Fixed | 108 | 1087 | 0.52 (0.49, 0.56) | 0.00 | 9.1 |
| 24 | 24 | Social-Emotional Z-Score | 50th | Random | 108 | 1087 | 0.52 (0.49, 0.56) | 0.00 | 9.1 |
| 24 | 24 | Social-Emotional Z-Score | 75th | Fixed | 108 | 1087 | 0.52 (0.48, 0.56) | 0.00 | 8.8 |
| 24 | 24 | Social-Emotional Z-Score | 75th | Random | 108 | 1087 | 0.52 (0.48, 0.56) | 0.00 | 8.8 |
| 27 | 27 | Gross Motor Z-Score | 25th | Fixed | 116 | 871 | 0.53 (0.48, 0.58) | 0.00 | 6.4 |
| 27 | 27 | Gross Motor Z-Score | 25th | Random | 116 | 871 | 0.53 (0.48, 0.58) | 0.00 | 6.4 |
| 27 | 27 | Gross Motor Z-Score | 50th | Fixed | 115 | 871 | 0.54 (0.50, 0.58) | 0.00 | 7.1 |
| 27 | 27 | Gross Motor Z-Score | 50th | Random | 115 | 871 | 0.54 (0.50, 0.58) | 0.00 | 7.1 |
| 27 | 27 | Gross Motor Z-Score | 75th | Fixed | 115 | 871 | 0.54 (0.49, 0.58) | 0.00 | 6.4 |
| 27 | 27 | Gross Motor Z-Score | 75th | Random | 115 | 871 | 0.54 (0.49, 0.58) | 0.00 | 6.4 |
| 27 | 27 | Language Z-Score | 25th | Fixed | 113 | 874 | 0.53 (0.49, 0.57) | 0.00 | 7.8 |
| 27 | 27 | Language Z-Score | 25th | Random | 113 | 874 | 0.53 (0.49, 0.57) | 0.00 | 7.8 |
| 27 | 27 | Language Z-Score | 50th | Fixed | 116 | 874 | 0.51 (0.47, 0.55) | 0.00 | 5.7 |
| 27 | 27 | Language Z-Score | 50th | Random | 116 | 874 | 0.51 (0.47, 0.55) | 0.00 | 5.7 |
| 27 | 27 | Language Z-Score | 75th | Fixed | 117 | 874 | 0.53 (0.48, 0.58) | 0.28 | 7.1 |
| 27 | 27 | Language Z-Score | 75th | Random | 117 | 874 | 0.52 (0.47, 0.58) | 0.28 | 7.1 |
| 27 | 27 | Motor Z-Score | 25th | Fixed | 116 | 871 | 0.52 (0.47, 0.57) | 0.27 | 7.8 |
| 27 | 27 | Motor Z-Score | 25th | Random | 116 | 871 | 0.52 (0.47, 0.58) | 0.27 | 7.8 |
| 27 | 27 | Motor Z-Score | 50th | Fixed | 115 | 871 | 0.53 (0.49, 0.57) | 0.42 | 5.7 |
| 27 | 27 | Motor Z-Score | 50th | Random | 115 | 871 | 0.53 (0.47, 0.59) | 0.42 | 5.7 |
| 27 | 27 | Motor Z-Score | 75th | Fixed | 118 | 871 | 0.53 (0.48, 0.57) | 0.00 | 5.7 |
| 27 | 27 | Motor Z-Score | 75th | Random | 118 | 871 | 0.53 (0.48, 0.57) | 0.00 | 5.7 |
| 27 | 27 | Social-Emotional Z-Score | 25th | Fixed | 111 | 866 | 0.50 (0.45, 0.55) | 0.00 | 6.4 |
| 27 | 27 | Social-Emotional Z-Score | 25th | Random | 111 | 866 | 0.50 (0.45, 0.55) | 0.00 | 6.4 |
| 27 | 27 | Social-Emotional Z-Score | 50th | Fixed | 115 | 866 | 0.51 (0.47, 0.55) | 0.00 | 6.4 |
| 27 | 27 | Social-Emotional Z-Score | 50th | Random | 115 | 866 | 0.51 (0.47, 0.55) | 0.00 | 6.4 |
| 27 | 27 | Social-Emotional Z-Score | 75th | Fixed | 117 | 866 | 0.50 (0.45, 0.54) | 0.00 | 5.7 |
| 27 | 27 | Social-Emotional Z-Score | 75th | Random | 117 | 866 | 0.50 (0.45, 0.54) | 0.00 | 5.7 |
| 30 | 30 | Gross Motor Z-Score | 25th | Fixed | 112 | 737 | 0.51 (0.46, 0.56) | 0.25 | 4.9 |
| 30 | 30 | Gross Motor Z-Score | 25th | Random | 112 | 737 | 0.53 (0.43, 0.62) | 0.25 | 4.9 |
| 30 | 30 | Gross Motor Z-Score | 50th | Fixed | 112 | 737 | 0.52 (0.48, 0.57) | 0.00 | 0.0 |
| 30 | 30 | Gross Motor Z-Score | 50th | Random | 112 | 737 | 0.52 (0.48, 0.57) | 0.00 | 0.0 |
| 30 | 30 | Gross Motor Z-Score | 75th | Fixed | 114 | 737 | 0.51 (0.46, 0.56) | 0.10 | 0.4 |
| 30 | 30 | Gross Motor Z-Score | 75th | Random | 114 | 737 | 0.51 (0.46, 0.57) | 0.10 | 0.4 |
| 30 | 30 | Language Z-Score | 25th | Fixed | 112 | 749 | 0.53 (0.49, 0.58) | 0.00 | 6.4 |
| 30 | 30 | Language Z-Score | 25th | Random | 112 | 749 | 0.53 (0.49, 0.58) | 0.00 | 6.4 |
| 30 | 30 | Language Z-Score | 50th | Fixed | 114 | 749 | 0.52 (0.48, 0.56) | 0.64 | 8.5 |
| 30 | 30 | Language Z-Score | 50th | Random | 114 | 749 | 0.48 (0.37, 0.60) | 0.64 | 8.5 |
| 30 | 30 | Language Z-Score | 75th | Fixed | 114 | 749 | 0.54 (0.49, 0.58) | 0.00 | 9.9 |
| 30 | 30 | Language Z-Score | 75th | Random | 114 | 749 | 0.54 (0.49, 0.58) | 0.00 | 9.9 |
| 30 | 30 | Motor Z-Score | 25th | Fixed | 112 | 737 | 0.49 (0.44, 0.54) | 0.43 | 5.7 |
| 30 | 30 | Motor Z-Score | 25th | Random | 112 | 737 | 0.52 (0.40, 0.65) | 0.43 | 5.7 |
| 30 | 30 | Motor Z-Score | 50th | Fixed | 112 | 737 | 0.51 (0.47, 0.56) | 0.00 | 0.0 |
| 30 | 30 | Motor Z-Score | 50th | Random | 112 | 737 | 0.51 (0.47, 0.56) | 0.00 | 0.0 |
| 30 | 30 | Motor Z-Score | 75th | Fixed | 111 | 737 | 0.52 (0.47, 0.57) | 0.00 | 1.8 |
| 30 | 30 | Motor Z-Score | 75th | Random | 111 | 737 | 0.52 (0.47, 0.57) | 0.00 | 1.8 |
| 30 | 30 | Social-Emotional Z-Score | 25th | Fixed | 112 | 740 | 0.50 (0.46, 0.55) | 0.00 | 6.4 |
| 30 | 30 | Social-Emotional Z-Score | 25th | Random | 112 | 740 | 0.50 (0.46, 0.55) | 0.00 | 6.4 |
| 30 | 30 | Social-Emotional Z-Score | 50th | Fixed | 114 | 740 | 0.49 (0.44, 0.53) | 0.00 | 7.1 |
| 30 | 30 | Social-Emotional Z-Score | 50th | Random | 114 | 740 | 0.49 (0.44, 0.53) | 0.00 | 7.1 |
| 30 | 30 | Social-Emotional Z-Score | 75th | Fixed | 112 | 740 | 0.47 (0.41, 0.52) | 0.61 | 7.1 |
| 30 | 30 | Social-Emotional Z-Score | 75th | Random | 112 | 740 | 0.43 (0.29, 0.57) | 0.61 | 7.1 |

**Supplemental Table 4**. Best Hb discriminatory thresholds that were identified for each outcome and age.

| Age of Hb measurement | Outcome | N studies; children | Hb threshold (SD across studies) that best discriminated the **25^th^ percentile** of the outcome: | Hb threshold (SD across studies) that best discriminated the **50^th^ percentile** of the outcome: | Hb threshold (SD across studies) that best discriminated the **75^th^ percentile** of the outcome: | Shape of the association |
| --- | --- | --- | --- | --- | --- | --- |
|  |  |  | Mean or prevalence difference in outcome below vs above the Hb threshold; | Mean or prevalence difference in outcome below vs above the Hb threshold; | Mean or prevalence difference in outcome below vs above the Hb threshold; |  |
|  |  |  | U = Unadjusted; | U = Unadjusted; | U = Unadjusted; |  |
|  |  |  | A = Adjusted for covariates; | A = Adjusted for covariates; | A = Adjusted for covariates; |  |
|  |  |  | I^2^ for pooled AUC | I^2^ for pooled AUC | I^2^ for pooled AUC |  |
| 6 mo | Walking alone at 12 mo | 5; 3982 |  | 109 (3.1) g/L |  | The threshold represents a plateau point, with steep downward slope in the outcome below 109 g/L and slight downward slope above 109 g/L. |
|  |  |  |  | U: -5 percentage points* |  |  |
|  |  |  |  | A: -4 percentage points* |  |  |
|  |  |  |  | I^2^ = 0.00 |  |  |
| 6 mo | 12-Mo Sleep Index Z-Score | 2; 1455 | 113 (7.8) g/L | 113 (7.8) g/L | 113 (8.5) g/L | No meaningful difference in the outcome above vs below the Hb threshold. |
|  |  |  | U: 0.03 SD | U: 0.03 SD | U: 0.03 SD |  |
|  |  |  | A: 0.03 SD | A: 0.03 SD | A: 0.03 SD |  |
|  |  |  | I^2^ = 0.52 | I^2^ = 0.09 | I^2^ = 0 |  |
| 6 mo | 18-Mo Nap Index Z-Score | 2; 1456 | 112 (9.2) g/L | 113 (5.7) g/L | 112 (5.7) g/L | No meaningful difference in the outcome above vs below the Hb threshold. |
|  |  |  | U: -0.04 SD | U: -0.01 SD | U: -0.04 SD |  |
|  |  |  | A: 0.00 SD | A: -0.03 SD | A: 0.00 SD |  |
|  |  |  | I^2^ = 0.87 | I^2^ = 0.72 | I^2^ = 0 |  |
| 6 mo | 18-Mo Gross Motor Z-Score | 5; 3621 | 108 (4.9) g/L | 108 (4.2) g/L | 108 (4.4) g/L | No meaningful difference in the outcome above vs below the Hb threshold. |
|  |  |  | U: -0.06 SD† | U: -0.06 SD† | U: -0.06 SD† |  |
|  |  |  | A: -0.06 SD† | A: -0.06 SD† | A: -0.06 SD† |  |
|  |  |  | I^2^ = 0.36 | I^2^ = 0 | I^2^ = 0 |  |
| 6 mo | 24-Mo Social-Emotional Z-Score | 2; 1529 | 107 (7.1) g/L | 106 (7.1) g/L | 109 (6.4) g/L | No meaningful difference in the outcome above vs below the Hb threshold. |
|  |  |  | U: -0.05 SD | U: -0.05 SD | U: -0.03 SD |  |
|  |  |  | A: -0.03 SD | A: -0.04 SD | A: -0.01 SD |  |
|  |  |  | I^2^ = 0 | I^2^ = 0 | I^2^ = 0.58 |  |
| 12 mo | Walking alone at 12 mos | 3; 832 |  | 106 (5.0) g/L |  | The threshold represents a plateau point, with the outcome generally higher above 106 g/L, however the difference between high and low Hb groups was attenuated when adjusting for covariates. |
|  |  |  |  | U: -6 percentage points† |  |  |
|  |  |  |  | A: -4 percentage points |  |  |
|  |  |  |  | I^2^ = 0 |  |  |
| 12 mo | 12-Mo Motor Z-Score | 2; 752 | 104 (2.8) g/L | 105 (0.7) g/L | 105 (0.7) g/L | No meaningful difference in the outcome above vs below the Hb threshold. |
|  |  |  | U: -0.03 SD | U: -0.04 SD | U: -0.04 SD |  |
|  |  |  | A: -0.04 SD | A: -0.05 SD | A: -0.05 SD |  |
|  |  |  | I^2^ = 0 | I^2^ = 0 | I^2^ = 0 |  |
| 12 mo | 24-Mo Language Z-Score | 2; 855 | 111 (7.1) g/L | 111 (7.1) g/L | 111 (6.4) g/L | The threshold represents a plateau point, with the outcome generally higher above 111 g/L, however the difference between high and low Hb groups was attenuated when adjusting for covariates. |
|  |  |  | U: -0.12 SD† | U: -0.12 SD† | U: -0.12 SD† |  |
|  |  |  | A: -0.10 SD | A: -0.10 SD | A: -0.10 SD |  |
|  |  |  | I^2^ = 0.56 | I^2^ = 0 | I^2^ = 0.62 |  |
| 12 mo | 24-Mo Social-Emotional Z-Score | 2; 865 | 109 (7.8) g/L | 110 (7.8) g/L | 109 (7.1) g/L | The threshold represents a plateau point, with the outcome generally higher above 110 g/L. |
|  |  |  | U: -0.16 SD* | U: -0.16 SD* | U: -0.16 SD* |  |
|  |  |  | A: -0.15 SD† | A: -0.15 SD† | A: -0.15 SD† |  |
|  |  |  | I^2^ = 0.65 | I^2^ = 0 | I^2^ = 0.32 |  |
| 15 mo | 15-Mo Language Z-Score | 2; 539 | 104 (5.7) g/L | 101 (8.5) g/L | 105 (6.4) g/L | No meaningful difference in the outcome above vs below the Hb threshold. |
|  |  |  | U: -0.02 SD | U: 0.07 SD | U: 0.01 SD |  |
|  |  |  | A: -0.01 SD | A: 0.07 SD | A: -0.01 SD |  |
|  |  |  | I^2^ = 0.81 | I^2^ = 0.84 | I^2^ = 0.83 |  |
| 15 mo | 15-Mo Social-Emotional Z-Score | 2; 539 | 97 (4.9) g/L | 98 (7.1) g/L | 104 (9.2) g/L | No meaningful difference in the outcome above vs below the Hb threshold. |
|  |  |  | U: 0.03 SD | U: 0.06 SD | U: -0.02 SD |  |
|  |  |  | A: 0.03 SD | A: 0.06 SD | A: -0.02 SD |  |
|  |  |  | I^2^ = 0.89 | I^2^ = 0.92 | I^2^ = 0.79 |  |
| 15 mo | 15-Mo Gross Motor Z-Score | 2; 541 | 104 (4.9) g/L | 104 (3.5) g/L | 104 (4.9) g/L | The threshold represents a plateau point, with the outcome generally higher above 104 g/L. |
|  |  |  | U: -0.18 SD* | U: -0.18 SD* | U: -0.18 SD* |  |
|  |  |  | A: -0.14 SD* | A: -0.14 SD* | A: -0.14 SD* |  |
|  |  |  | I^2^ = 0 | I^2^ = 0 | I^2^ = 0 |  |
| 18 mo | 18-Mo Language Z-Score | 7; 4963 | 103 (6.1) g/L | 109 (6.8) g/L | 110 (6.6) g/L | The threshold represents a plateau point, with steep downward slope in the outcome below 103 g/L and slight upward slope above 103 g/L. |
|  |  |  | U: -0.21 SD* | U: -0.18 SD* | U: -0.18 SD* |  |
|  |  |  | A: -0.15 SD* | A: -0.12 SD* | A: -0.13 SD* |  |
|  |  |  | I^2^ = 0.31 | I^2^ = 0.65 | I^2^ = 0.36 |  |
| 18 mo | 18-Mo Social-Emotional Z-Score | 7; 4952 | 104 (6.5) g/L | 110 (7.6) g/L | 109 (6.5) g/L | There is a gradual upward slope in the outcome across the full range of Hb. |
|  |  |  | U: -0.10 SD* | U: -0.09 SD* | U: -0.10 SD* |  |
|  |  |  | A: -0.07 SD* | A: -0.06 SD† | A: -0.07 SD* |  |
|  |  |  | I^2^ = 0.19 | I^2^ = 0 | I^2^ = 0.08 |  |
| 18 mo | 18-Mo Motor Z-Score | 7; 4781 | 104 (6.9) g/L | 108 (7.8) g/L | 109 (7.7) g/L | The threshold represents a plateau point, with steep downward slope in the outcome below 107 g/L and slight downward slope above 112 g/L. |
|  |  |  | U: -0.19 SD* | U: -0.18 SD* | U: -0.16 SD* |  |
|  |  |  | A: -0.14 SD* | A: -0.12 SD* | A: -0.11 SD* |  |
|  |  |  | I^2^ = 0.61 | I^2^ = 0.75 | I^2^ = 0.45 |  |
| 18 mo | 18-Mo Gross Motor Z-Score | 6; 3684 | 108 (5.7) g/L | 110 (5.1) g/L | 110 (5.3) g/L | There is a slight downward slope in the outcome at both higher and lower values of Hb. |
|  |  |  | U: -0.12 SD* | U: -0.10 SD* | U: -0.10 SD* |  |
|  |  |  | A: -0.09 SD* | A: -0.08 SD* | A: -0.08 SD* |  |
|  |  |  | I^2^ = 0.47 | I^2^ = 0.14 | I^2^ = 0.03 |  |
| 18 mo | 18-Mo Fine Motor Z-Score | 6; 3753 | 109 (4.8) g/L | 110 (5.1) g/L | 113 (4.9) g/L | The threshold represents a plateau point, with steep downward slope in the outcome below 110 g/L and slight downward slope above 113 g/L, however the difference between high and low Hb groups was attenuated when adjusting for covariates. |
|  |  |  | U: -0.09 SD* | U: -0.08 SD* | U: -0.05 SD |  |
|  |  |  | A: -0.05 SD | A: -0.04 SD | A: -0.01 SD |  |
|  |  |  | I^2^ = 0.73 | I^2^ = 0.63 | I^2^ = 0.41 |  |
| 18 mo | 18-Mo Mean Vector Magnitude Z-Score | 2; 955 | 102 (6.4) g/L | 106 (4.9) g/L | 109 (6.4) g/L | The threshold represents a plateau point, with steep downward slope in the outcome below 102 g/L |
|  |  |  | U: -0.38 SD* | U: -0.29 SD* | U: -0.28 SD* |  |
|  |  |  | A: -0.38 SD* | A: -0.30 SD* | A: -0.28 SD* |  |
|  |  |  | I^2^ = 0 | I^2^ = 0.44 | I^2^ = 0.12 |  |
| 18 mo | 18-Mo % time spent in MVPA Z-Score | 2; 955 | 110 (4.9) g/L | 107 (3.5) g/L | 111 (0.7) g/L | The threshold represents a plateau point, with slight downward slope in the outcome below 107 g/L |
|  |  |  | U: -0.14 SD* | U: -0.11 SD† | U: -0.15 SD* |  |
|  |  |  | A: -0.13 SD† | A: -0.11 SD | A: -0.16 SD* |  |
|  |  |  | I^2^ = 0.17 | I^2^ = 0 | I^2^ = 0.49 |  |
| 18 mo | 18-Mo % time spent in sedentary behavior Z-Score | 2; 955 | 108 (1.4) g/L | 109 (1.4) g/L | 106 (2.8) g/L | The threshold represents a plateau point, with steep upward slope in the outcome below 106 g/L |
|  |  |  | U: 0.31 SD* | U: 0.30 SD* | U: 0.29 SD* |  |
|  |  |  | A: 0.31 SD* | A: 0.30 SD* | A: 0.30 SD* |  |
|  |  |  | I^2^ = 0.66 | I^2^ = 0.50 | I^2^ = 0 |  |
| 18 mo | 18-Mo Nap Index Z-Score | 2; 1453 | 112 (2.8) g/L | 112 (2.1) g/L | 112 (2.1) g/L | No meaningful difference in the outcome above vs below the Hb threshold. |
|  |  |  | U: -0.04 SD | U: -0.04 SD | U: -0.04 SD |  |
|  |  |  | A: -0.04 SD | A: -0.04 SD | A: -0.04 SD |  |
|  |  |  | I^2^ = 0.00 | I^2^ = 0.00 | I^2^ = 0.07 |  |
| 18 mo | 24-Mo Language Z-Score | 2; 2839 | 116 (0.0) g/L | 116 (0.0) g/L | 116 (0.0) g/L | There is a slight downward slope in the outcome at both higher and lower values of Hb |
|  |  |  | U: -0.10 SD* | U: -0.10 SD* | U: -0.10 SD* |  |
|  |  |  | A: -0.07 SD† | A: -0.07 SD† | A: -0.07 SD† |  |
|  |  |  | I^2^ = 0 | I^2^ = 0 | I^2^ = 0 |  |
| 18 mo | 24-Mo Motor Z-Score | 2; 2824 | 116 (2.1) g/L | 116 (1.4) g/L | 117 (0.0) g/L | There is a slight downward slope in the outcome at both higher and lower values of Hb |
|  |  |  | U: -0.08 SD* | U: -0.08 SD* | U: -0.08 SD* |  |
|  |  |  | A: -0.06 SD† | A: -0.06 SD† | A: -0.07 SD* |  |
|  |  |  | I^2^ = 0 | I^2^ = 0 | I^2^ = 0 |  |
| 18 mo | 24-Mo Gross Motor Z-Score | 2; 2829 | 116 (2.1) g/L | 116 (0.7) g/L | 117 (1.4) g/L | No meaningful difference in the outcome above vs below the Hb threshold. |
|  |  |  | U: -0.06 SD† | U: -0.06 SD† | U: -0.07 SD* |  |
|  |  |  | A: -0.05 SD | A: -0.05 SD | A: -0.06 SD† |  |
|  |  |  | I^2^ = 0 | I^2^ = 0 | I^2^ = 0.24 |  |
| 21 mo | 21-Mo Language Z-Score | 3; 948 | 103 (7.5) g/L | 105 (12.5) g/L | 109 (11.5) g/L | The threshold represents a plateau point, with the outcome generally higher above 105 g/L, however the difference between high and low Hb groups was attenuated when adjusting for covariates. |
|  |  |  | U: -0.11 SD | U: -0.15 SD* | U: -0.16 SD* |  |
|  |  |  | A: -0.08 SD | A: -0.09 SD | A: -0.08 SD |  |
|  |  |  | I^2^ = 0.21 | I^2^ = 0.14 | I^2^ = 0.41 |  |
| 21 mo | 21-Mo Social-Emotional Z-Score | 3; 948 | 105 (9.0) g/L | 105 (8.7) g/L | 112 (7.5) g/L | The threshold represents a plateau point, with the outcome generally higher above 105 g/L, however the difference between high and low Hb groups was attenuated when adjusting for covariates. |
|  |  |  | U: -0.13 SD† | U: -0.13 SD† | U: -0.17 SD* |  |
|  |  |  | A: -0.08 SD | A: -0.08 SD | A: -0.09 SD |  |
|  |  |  | I^2^ = 0.75 | I^2^ = 0.42 | I^2^ = 0.53 |  |
| 21 mo | 21-Mo Motor Z-Score | 3; 942 | 105 (6.0) g/L | 106 (8.9) g/L | 109 (8.3) g/L | The threshold represents a plateau point, with the outcome generally higher above 105 g/L, however the difference between high and low Hb groups was attenuated when adjusting for covariates. |
|  |  |  | U: -0.11 SD† | U: -0.11 SD† | U: -0.10 SD |  |
|  |  |  | A: -0.06 SD | A: -0.05 SD | A: -0.04 SD |  |
|  |  |  | I^2^ = 0.40 | I^2^ = 0.85 | I^2^ = 0.77 |  |
| 21 mo | 24-Mo Language Z-Score | 3; 409 | 116 (1.5) g/L | 116 (1.5) g/L | 117 (1.2) g/L | The threshold represents a plateau point, with the outcome generally higher above 116 g/L. |
|  |  |  | U: -0.24 SD* | U: -0.24 SD* | U: -0.21 SD* |  |
|  |  |  | A: -0.21 SD* | A: -0.21 SD* | A: -0.20 SD* |  |
|  |  |  | I^2^ = 0 | I^2^ = 0 | I^2^ = 0 |  |
| 24 mo | 24-Mo Language Z-Score | 4; 1091 | 107 (11.6) g/L | 108 (9.0) g/L | 108 (8.6) g/L | The threshold represents a plateau point, with the outcome generally higher above 107 g/L. |
|  |  |  | U: -0.16 SD* | U: -0.14 SD* | U: -0.14 SD* |  |
|  |  |  | A: -0.19 SD* | A: -0.18 SD* | A: -0.18 SD* |  |
|  |  |  | I^2^ = 0.40 | I^2^ = 0 | I^2^ = 0 |  |
| 24 mo | 24-Mo Social-Emotional Z-Score | 4; 1087 | 107 (7.0) g/L | 108 (9.1) g/L | 108 (8.8) g/L | The threshold represents a plateau point, with a steep downward slope in the outcome below 108 g/L and slight downward slope above 117 g/L. |
|  |  |  | U: -0.12 SD† | U: -0.13 SD† | U: -0.13 SD† |  |
|  |  |  | A: -0.14 SD† | A: -0.14 SD† | A: -0.14 SD† |  |
|  |  |  | I^2^ = 0 | I^2^ = 0 | I^2^ = 0 |  |
| 24 mo | 24-Mo Motor Z-Score | 4; 1082 | 108 (10.3) g/L | 108 (10.0) g/L | 107 (8.6) g/L | The threshold represents a plateau point, with the outcome generally higher above 108 g/L, however the difference between high and low Hb groups was attenuated when adjusting for covariates. |
|  |  |  | U: -0.10 SD† | U: -0.10 SD† | U: -0.08 SD |  |
|  |  |  | A: -0.08 SD | A: -0.08 SD | A: -0.05 SD |  |
|  |  |  | I^2^ = 0 | I^2^ = 0 | I^2^ = 0 |  |
| 27 mo | 27-Mo Gross Motor Z-Score | 2; 871 | 116 (6.4) g/L | 115 (7.1) g/L | 115 (6.4) g/L | There is a gradual upward slope in the outcome across the full range of Hb. |
|  |  |  | U: -0.11 SD | U: -0.11 SD | U: -0.11 SD |  |
|  |  |  | A: -0.13 SD† | A: -0.12 SD† | A: -0.12 SD† |  |
|  |  |  | I^2^ = 0 | I^2^ = 0 | I^2^ = 0 |  |

U: Unadjusted mean difference or prevalence difference above versus below the identified best Hb threshold

A: Mean difference or prevalence difference adjusted for intervention arm and the following covariates that were available in the dataset: maternal age, maternal education, household asset index, household food insecurity, number of children < 5 y in the household, child sex, and home environment.

**p* < 0.05

†0.05 < *p* < 0.1

**Supplemental Table 5**. Mean difference in Hb above vs below candidate Hb threshold for all Hb-outcome associations that met the minimum sample size criteria

| **Age of Hb measurement** | **Age at outcome measurement** | **Outcome** | **Candidate Hb (g/L)** | **Pooling method** | **N children below Hb** | **N children above Hb** | **Mean difference (95% CI)** | **I^2^** | **P-value for MD** |
| --- | --- | --- | --- | --- | --- | --- | --- | --- | --- |
| 9 | 9 | Language Z-Score | 103 | Fixed | 391 | 406 | 0.06 (-0.08, 0.20) | 0.90 | 0.371 |
| 9 | 9 | Language Z-Score | 103 | Random | 391 | 406 | 0.03 (-0.42, 0.49) | 0.90 | 0.882 |
| 9 | 9 | Language Z-Score | 104 | Fixed | 425 | 372 | 0.07 (-0.08, 0.21) | 0.90 | 0.358 |
| 9 | 9 | Language Z-Score | 104 | Random | 425 | 372 | 0.03 (-0.42, 0.48) | 0.90 | 0.900 |
| 9 | 9 | Language Z-Score | 105 | Fixed | 453 | 344 | 0.07 (-0.07, 0.21) | 0.90 | 0.327 |
| 9 | 9 | Language Z-Score | 105 | Random | 453 | 344 | 0.02 (-0.43, 0.48) | 0.90 | 0.917 |
| 9 | 9 | Language Z-Score | 110 | Fixed | 577 | 220 | 0.17 (0.01, 0.34) | 0.88 | 0.043 |
| 9 | 9 | Language Z-Score | 110 | Random | 577 | 220 | 0.13 (-0.38, 0.63) | 0.88 | 0.626 |
| 9 | 9 | Social-Emotional Z-Score | 100 | Fixed | 308 | 489 | 0.06 (-0.08, 0.20) | 0.91 | 0.410 |
| 9 | 9 | Social-Emotional Z-Score | 100 | Random | 308 | 489 | 0.06 (-0.42, 0.54) | 0.91 | 0.812 |
| 9 | 9 | Social-Emotional Z-Score | 104 | Fixed | 425 | 372 | 0.10 (-0.04, 0.24) | 0.84 | 0.162 |
| 9 | 9 | Social-Emotional Z-Score | 104 | Random | 425 | 372 | 0.08 (-0.26, 0.43) | 0.84 | 0.629 |
| 9 | 9 | Social-Emotional Z-Score | 105 | Fixed | 453 | 344 | 0.10 (-0.04, 0.24) | 0.84 | 0.152 |
| 9 | 9 | Social-Emotional Z-Score | 105 | Random | 453 | 344 | 0.08 (-0.28, 0.43) | 0.84 | 0.677 |
| 9 | 9 | Social-Emotional Z-Score | 110 | Fixed | 577 | 220 | 0.13 (-0.03, 0.29) | 0.90 | 0.102 |
| 9 | 9 | Social-Emotional Z-Score | 110 | Random | 577 | 220 | 0.12 (-0.40, 0.63) | 0.90 | 0.654 |
| 12 | 12 | Motor Z-Score | 104 | Fixed | 427 | 325 | -0.03 (-0.19, 0.12) | 0.00 | 0.664 |
| 12 | 12 | Motor Z-Score | 104 | Random | 427 | 325 | -0.03 (-0.19, 0.12) | 0.00 | 0.664 |
| 12 | 12 | Motor Z-Score | 105 | Fixed | 443 | 309 | -0.04 (-0.20, 0.12) | 0.00 | 0.619 |
| 12 | 12 | Motor Z-Score | 105 | Random | 443 | 309 | -0.04 (-0.20, 0.12) | 0.00 | 0.619 |
| 12 | 12 | Motor Z-Score | 110 | Fixed | 561 | 191 | -0.02 (-0.22, 0.17) | 0.26 | 0.801 |
| 12 | 12 | Motor Z-Score | 110 | Random | 561 | 191 | -0.02 (-0.25, 0.21) | 0.26 | 0.863 |
| 12 | 12 | Walking alone | 106 | Fixed | 412 | 420 | -0.06 (-0.12, 0.00) | 0.57 | 0.059 |
| 12 | 12 | Walking alone | 106 | Random | 412 | 420 | -0.06 (-0.15, 0.03) | 0.57 | 0.215 |
| 12 | 12 | Walking alone | 110 | Fixed | 502 | 330 | -0.04 (-0.10, 0.02) | 0.59 | 0.234 |
| 12 | 12 | Walking alone | 110 | Random | 502 | 330 | -0.04 (-0.13, 0.06) | 0.59 | 0.431 |
| 15 | 15 | Language Z-Score | 101 | Fixed | 261 | 278 | 0.07 (-0.08, 0.23) | 0.66 | 0.360 |
| 15 | 15 | Language Z-Score | 101 | Random | 261 | 278 | 0.03 (-0.25, 0.32) | 0.66 | 0.811 |
| 15 | 15 | Language Z-Score | 104 | Fixed | 315 | 224 | -0.02 (-0.17, 0.14) | 0.84 | 0.834 |
| 15 | 15 | Language Z-Score | 104 | Random | 315 | 224 | -0.06 (-0.47, 0.34) | 0.84 | 0.760 |
| 15 | 15 | Language Z-Score | 105 | Fixed | 334 | 205 | 0.01 (-0.16, 0.18) | 0.76 | 0.887 |
| 15 | 15 | Language Z-Score | 105 | Random | 334 | 205 | -0.04 (-0.42, 0.33) | 0.76 | 0.819 |
| 15 | 15 | Language Z-Score | 110 | Fixed | 403 | 136 | 0.05 (-0.11, 0.22) | 0.81 | 0.536 |
| 15 | 15 | Language Z-Score | 110 | Random | 403 | 136 | -0.01 (-0.41, 0.40) | 0.81 | 0.977 |
| 15 | 15 | Social-Emotional Z-Score | 97 | Fixed | 195 | 344 | 0.03 (-0.14, 0.19) | 0.81 | 0.748 |
| 15 | 15 | Social-Emotional Z-Score | 97 | Random | 195 | 344 | -0.11 (-0.61, 0.39) | 0.81 | 0.671 |
| 15 | 15 | Social-Emotional Z-Score | 98 | Fixed | 209 | 330 | 0.06 (-0.09, 0.22) | 0.81 | 0.424 |
| 15 | 15 | Social-Emotional Z-Score | 98 | Random | 209 | 330 | -0.05 (-0.51, 0.40) | 0.81 | 0.813 |
| 15 | 15 | Social-Emotional Z-Score | 104 | Fixed | 315 | 224 | -0.02 (-0.19, 0.15) | 0.88 | 0.810 |
| 15 | 15 | Social-Emotional Z-Score | 104 | Random | 315 | 224 | -0.12 (-0.65, 0.41) | 0.88 | 0.649 |
| 15 | 15 | Social-Emotional Z-Score | 110 | Fixed | 403 | 136 | -0.01 (-0.18, 0.16) | 0.92 | 0.897 |
| 15 | 15 | Social-Emotional Z-Score | 110 | Random | 403 | 136 | -0.16 (-0.85, 0.54) | 0.92 | 0.661 |
| 15 | 15 | Gross Motor Z-Score | 104 | Fixed | 317 | 224 | -0.18 (-0.31, -0.05) | 0.00 | 0.009 |
| 15 | 15 | Gross Motor Z-Score | 104 | Random | 317 | 224 | -0.18 (-0.31, -0.05) | 0.00 | 0.009 |
| 15 | 15 | Gross Motor Z-Score | 110 | Fixed | 404 | 137 | -0.19 (-0.32, -0.05) | 0.00 | 0.006 |
| 15 | 15 | Gross Motor Z-Score | 110 | Random | 404 | 137 | -0.19 (-0.32, -0.05) | 0.00 | 0.006 |
| 18 | 18 | Language Z-Score | 103 | Fixed | 1834 | 3129 | -0.21 (-0.28, -0.15) | 0.59 | 0.000 |
| 18 | 18 | Language Z-Score | 103 | Random | 1834 | 3129 | -0.22 (-0.33, -0.12) | 0.59 | 0.000 |
| 18 | 18 | Language Z-Score | 109 | Fixed | 2643 | 2320 | -0.18 (-0.24, -0.12) | 0.32 | 0.000 |
| 18 | 18 | Language Z-Score | 109 | Random | 2643 | 2320 | -0.18 (-0.26, -0.09) | 0.32 | 0.000 |
| 18 | 18 | Language Z-Score | 110 | Fixed | 2771 | 2192 | -0.18 (-0.24, -0.12) | 0.44 | 0.000 |
| 18 | 18 | Language Z-Score | 110 | Random | 2771 | 2192 | -0.18 (-0.27, -0.08) | 0.44 | 0.000 |
| 18 | 18 | Social-Emotional Z-Score | 104 | Fixed | 1971 | 2981 | -0.10 (-0.17, -0.04) | 0.00 | 0.001 |
| 18 | 18 | Social-Emotional Z-Score | 104 | Random | 1971 | 2981 | -0.10 (-0.17, -0.04) | 0.00 | 0.001 |
| 18 | 18 | Social-Emotional Z-Score | 109 | Fixed | 2637 | 2315 | -0.10 (-0.16, -0.04) | 0.38 | 0.002 |
| 18 | 18 | Social-Emotional Z-Score | 109 | Random | 2637 | 2315 | -0.10 (-0.18, -0.02) | 0.38 | 0.018 |
| 18 | 18 | Social-Emotional Z-Score | 110 | Fixed | 2765 | 2187 | -0.09 (-0.15, -0.02) | 0.46 | 0.008 |
| 18 | 18 | Social-Emotional Z-Score | 110 | Random | 2765 | 2187 | -0.08 (-0.17, 0.01) | 0.46 | 0.069 |
| 18 | 18 | Motor Z-Score | 104 | Fixed | 1933 | 2848 | -0.19 (-0.25, -0.13) | 0.54 | 0.000 |
| 18 | 18 | Motor Z-Score | 104 | Random | 1933 | 2848 | -0.19 (-0.28, -0.09) | 0.54 | 0.000 |
| 18 | 18 | Motor Z-Score | 108 | Fixed | 2440 | 2341 | -0.18 (-0.24, -0.12) | 0.64 | 0.000 |
| 18 | 18 | Motor Z-Score | 108 | Random | 2440 | 2341 | -0.18 (-0.27, -0.08) | 0.64 | 0.000 |
| 18 | 18 | Motor Z-Score | 109 | Fixed | 2572 | 2209 | -0.16 (-0.22, -0.10) | 0.55 | 0.000 |
| 18 | 18 | Motor Z-Score | 109 | Random | 2572 | 2209 | -0.17 (-0.25, -0.08) | 0.55 | 0.000 |
| 18 | 18 | Motor Z-Score | 110 | Fixed | 2693 | 2088 | -0.15 (-0.21, -0.09) | 0.58 | 0.000 |
| 18 | 18 | Motor Z-Score | 110 | Random | 2693 | 2088 | -0.15 (-0.24, -0.07) | 0.58 | 0.001 |
| 18 | 18 | Gross Motor Z-Score | 108 | Fixed | 1577 | 2107 | -0.12 (-0.18, -0.05) | 0.49 | 0.000 |
| 18 | 18 | Gross Motor Z-Score | 108 | Random | 1577 | 2107 | -0.12 (-0.22, -0.03) | 0.49 | 0.008 |
| 18 | 18 | Gross Motor Z-Score | 110 | Fixed | 1801 | 1883 | -0.10 (-0.16, -0.04) | 0.47 | 0.002 |
| 18 | 18 | Gross Motor Z-Score | 110 | Random | 1801 | 1883 | -0.10 (-0.19, -0.02) | 0.47 | 0.020 |
| 18 | 18 | grossmotor_lowerhb | 102 | Fixed | 966 | 1338 | -0.14 (-0.21, -0.06) | 0.31 | 0.000 |
| 18 | 18 | grossmotor_lowerhb | 102 | Random | 966 | 1338 | -0.15 (-0.24, -0.05) | 0.31 | 0.002 |
| 18 | 18 | grossmotor_lowerhb | 103 | Fixed | 1067 | 1237 | -0.11 (-0.18, -0.04) | 0.34 | 0.002 |
| 18 | 18 | grossmotor_lowerhb | 103 | Random | 1067 | 1237 | -0.12 (-0.22, -0.03) | 0.34 | 0.011 |
| 18 | 18 | grossmotor_lowerhb | 104 | Fixed | 1176 | 1128 | -0.13 (-0.20, -0.05) | 0.22 | 0.001 |
| 18 | 18 | grossmotor_lowerhb | 104 | Random | 1176 | 1128 | -0.13 (-0.22, -0.05) | 0.22 | 0.003 |
| 18 | 18 | grossmotor_lowerhb | 110 | Fixed | 1801 | 503 | -0.13 (-0.21, -0.06) | 0.00 | 0.001 |
| 18 | 18 | grossmotor_lowerhb | 110 | Random | 1801 | 503 | -0.13 (-0.21, -0.06) | 0.00 | 0.001 |
| 18 | 18 | Fine Motor Z-Score | 109 | Fixed | 1714 | 2039 | -0.09 (-0.16, -0.02) | 0.40 | 0.016 |
| 18 | 18 | Fine Motor Z-Score | 109 | Random | 1714 | 2039 | -0.11 (-0.20, -0.01) | 0.40 | 0.028 |
| 18 | 18 | Fine Motor Z-Score | 110 | Fixed | 1823 | 1930 | -0.08 (-0.15, -0.01) | 0.31 | 0.030 |
| 18 | 18 | Fine Motor Z-Score | 110 | Random | 1823 | 1930 | -0.09 (-0.18, 0.00) | 0.31 | 0.040 |
| 18 | 18 | Fine Motor Z-Score | 113 | Fixed | 2135 | 1618 | -0.05 (-0.12, 0.02) | 0.52 | 0.164 |
| 18 | 18 | Fine Motor Z-Score | 113 | Random | 2135 | 1618 | -0.08 (-0.19, 0.03) | 0.52 | 0.155 |
| 18 | 18 | Mean Vector Magnitude Z-Score | 102 | Fixed | 334 | 621 | -21.48 (-29.48, -13.49) | 0.21 | 0.000 |
| 18 | 18 | Mean Vector Magnitude Z-Score | 102 | Random | 334 | 621 | -21.70 (-30.80, -12.61) | 0.21 | 0.000 |
| 18 | 18 | Mean Vector Magnitude Z-Score | 107 | Fixed | 459 | 496 | -16.29 (-23.95, -8.63) | 0.21 | 0.000 |
| 18 | 18 | Mean Vector Magnitude Z-Score | 107 | Random | 459 | 496 | -16.57 (-25.35, -7.79) | 0.21 | 0.000 |
| 18 | 18 | Mean Vector Magnitude Z-Score | 109 | Fixed | 509 | 446 | -15.57 (-23.25, -7.90) | 0.00 | 0.000 |
| 18 | 18 | Mean Vector Magnitude Z-Score | 109 | Random | 509 | 446 | -15.57 (-23.25, -7.90) | 0.00 | 0.000 |
| 18 | 18 | Mean Vector Magnitude Z-Score | 110 | Fixed | 540 | 415 | -15.61 (-23.32, -7.90) | 0.46 | 0.000 |
| 18 | 18 | Mean Vector Magnitude Z-Score | 110 | Random | 540 | 415 | -16.46 (-27.37, -5.55) | 0.46 | 0.003 |
| 18 | 18 | % time spent in MVPA Z-Score | 103 | Fixed | 365 | 590 | -0.01 (-0.01, 0.00) | 0.00 | 0.027 |
| 18 | 18 | % time spent in MVPA Z-Score | 103 | Random | 365 | 590 | -0.01 (-0.01, 0.00) | 0.00 | 0.027 |
| 18 | 18 | % time spent in MVPA Z-Score | 107 | Fixed | 459 | 496 | 0.00 (-0.01, 0.00) | 0.00 | 0.080 |
| 18 | 18 | % time spent in MVPA Z-Score | 107 | Random | 459 | 496 | 0.00 (-0.01, 0.00) | 0.00 | 0.080 |
| 18 | 18 | % time spent in MVPA Z-Score | 110 | Fixed | 540 | 415 | -0.01 (-0.01, 0.00) | 0.00 | 0.040 |
| 18 | 18 | % time spent in MVPA Z-Score | 110 | Random | 540 | 415 | -0.01 (-0.01, 0.00) | 0.00 | 0.040 |
| 18 | 18 | % time spent in MVPA Z-Score | 111 | Fixed | 564 | 391 | -0.01 (-0.01, 0.00) | 0.00 | 0.024 |
| 18 | 18 | % time spent in MVPA Z-Score | 111 | Random | 564 | 391 | -0.01 (-0.01, 0.00) | 0.00 | 0.024 |
| 18 | 18 | % time spent in sedentary behavior Z-Score | 106 | Fixed | 432 | 523 | 0.02 (0.01, 0.04) | 0.62 | 0.000 |
| 18 | 18 | % time spent in sedentary behavior Z-Score | 106 | Random | 432 | 523 | 0.03 (0.01, 0.04) | 0.62 | 0.008 |
| 18 | 18 | % time spent in sedentary behavior Z-Score | 109 | Fixed | 509 | 446 | 0.03 (0.01, 0.04) | 0.00 | 0.000 |
| 18 | 18 | % time spent in sedentary behavior Z-Score | 109 | Random | 509 | 446 | 0.03 (0.01, 0.04) | 0.00 | 0.000 |
| 18 | 18 | % time spent in sedentary behavior Z-Score | 110 | Fixed | 540 | 415 | 0.03 (0.02, 0.04) | 0.00 | 0.000 |
| 18 | 18 | % time spent in sedentary behavior Z-Score | 110 | Random | 540 | 415 | 0.03 (0.02, 0.04) | 0.00 | 0.000 |
| 21 | 21 | Language Z-Score | 103 | Fixed | 415 | 533 | -0.11 (-0.25, 0.03) | 0.32 | 0.110 |
| 21 | 21 | Language Z-Score | 103 | Random | 415 | 533 | -0.12 (-0.29, 0.05) | 0.32 | 0.156 |
| 21 | 21 | Language Z-Score | 105 | Fixed | 478 | 470 | -0.15 (-0.28, -0.02) | 0.00 | 0.020 |
| 21 | 21 | Language Z-Score | 105 | Random | 478 | 470 | -0.15 (-0.28, -0.02) | 0.00 | 0.020 |
| 21 | 21 | Language Z-Score | 109 | Fixed | 576 | 372 | -0.16 (-0.30, -0.03) | 0.00 | 0.017 |
| 21 | 21 | Language Z-Score | 109 | Random | 576 | 372 | -0.16 (-0.30, -0.03) | 0.00 | 0.017 |
| 21 | 21 | Language Z-Score | 110 | Fixed | 599 | 349 | -0.15 (-0.29, 0.00) | 0.00 | 0.047 |
| 21 | 21 | Language Z-Score | 110 | Random | 599 | 349 | -0.15 (-0.29, 0.00) | 0.00 | 0.047 |
| 21 | 21 | Social-Emotional Z-Score | 105 | Fixed | 478 | 470 | -0.13 (-0.27, 0.00) | 0.41 | 0.058 |
| 21 | 21 | Social-Emotional Z-Score | 105 | Random | 478 | 470 | -0.16 (-0.37, 0.05) | 0.41 | 0.136 |
| 21 | 21 | Social-Emotional Z-Score | 110 | Fixed | 599 | 349 | -0.13 (-0.28, 0.02) | 0.34 | 0.082 |
| 21 | 21 | Social-Emotional Z-Score | 110 | Random | 599 | 349 | -0.17 (-0.39, 0.06) | 0.34 | 0.142 |
| 21 | 21 | Social-Emotional Z-Score | 112 | Fixed | 644 | 304 | -0.17 (-0.32, -0.01) | 0.44 | 0.037 |
| 21 | 21 | Social-Emotional Z-Score | 112 | Random | 644 | 304 | -0.24 (-0.54, 0.07) | 0.44 | 0.129 |
| 21 | 21 | Motor Z-Score | 105 | Fixed | 473 | 469 | -0.11 (-0.22, 0.00) | 0.62 | 0.057 |
| 21 | 21 | Motor Z-Score | 105 | Random | 473 | 469 | -0.24 (-0.68, 0.20) | 0.62 | 0.277 |
| 21 | 21 | Motor Z-Score | 106 | Fixed | 506 | 436 | -0.11 (-0.23, 0.01) | 0.67 | 0.071 |
| 21 | 21 | Motor Z-Score | 106 | Random | 506 | 436 | -0.26 (-0.73, 0.21) | 0.67 | 0.279 |
| 21 | 21 | Motor Z-Score | 109 | Fixed | 573 | 369 | -0.10 (-0.23, 0.03) | 0.72 | 0.147 |
| 21 | 21 | Motor Z-Score | 109 | Random | 573 | 369 | -0.25 (-0.73, 0.22) | 0.72 | 0.298 |
| 21 | 21 | Motor Z-Score | 110 | Fixed | 597 | 345 | -0.10 (-0.24, 0.04) | 0.71 | 0.170 |
| 21 | 21 | Motor Z-Score | 110 | Random | 597 | 345 | -0.25 (-0.73, 0.22) | 0.71 | 0.299 |
| 24 | 24 | Language Z-Score | 107 | Fixed | 442 | 649 | -0.16 (-0.28, -0.03) | 0.24 | 0.012 |
| 24 | 24 | Language Z-Score | 107 | Random | 442 | 649 | -0.12 (-0.33, 0.09) | 0.24 | 0.248 |
| 24 | 24 | Language Z-Score | 108 | Fixed | 471 | 620 | -0.14 (-0.26, -0.02) | 0.16 | 0.026 |
| 24 | 24 | Language Z-Score | 108 | Random | 471 | 620 | -0.14 (-0.34, 0.06) | 0.16 | 0.179 |
| 24 | 24 | Language Z-Score | 110 | Fixed | 531 | 560 | -0.21 (-0.33, -0.09) | 0.00 | 0.001 |
| 24 | 24 | Language Z-Score | 110 | Random | 531 | 560 | -0.21 (-0.33, -0.09) | 0.00 | 0.001 |
| 24 | 24 | Social-Emotional Z-Score | 107 | Fixed | 442 | 645 | -0.12 (-0.26, 0.01) | 0.06 | 0.075 |
| 24 | 24 | Social-Emotional Z-Score | 107 | Random | 442 | 645 | -0.13 (-0.27, 0.01) | 0.06 | 0.075 |
| 24 | 24 | Social-Emotional Z-Score | 108 | Fixed | 471 | 616 | -0.13 (-0.27, 0.00) | 0.36 | 0.054 |
| 24 | 24 | Social-Emotional Z-Score | 108 | Random | 471 | 616 | -0.18 (-0.36, 0.00) | 0.36 | 0.056 |
| 24 | 24 | Social-Emotional Z-Score | 110 | Fixed | 531 | 556 | -0.21 (-0.33, -0.08) | 0.47 | 0.001 |
| 24 | 24 | Social-Emotional Z-Score | 110 | Random | 531 | 556 | -0.27 (-0.46, -0.08) | 0.47 | 0.005 |
| 24 | 24 | Social-Emotional Z-Score_lowerhb | 107 | Fixed | 442 | 399 | -0.12 (-0.26, 0.03) | 0.26 | 0.125 |
| 24 | 24 | Social-Emotional Z-Score_lowerhb | 107 | Random | 442 | 399 | -0.15 (-0.33, 0.04) | 0.26 | 0.114 |
| 24 | 24 | Social-Emotional Z-Score_lowerhb | 108 | Fixed | 471 | 370 | -0.11 (-0.27, 0.04) | 0.48 | 0.147 |
| 24 | 24 | Social-Emotional Z-Score_lowerhb | 108 | Random | 471 | 370 | -0.20 (-0.45, 0.05) | 0.48 | 0.116 |
| 24 | 24 | Social-Emotional Z-Score_lowerhb | 110 | Fixed | 531 | 310 | -0.20 (-0.34, -0.06) | 0.51 | 0.005 |
| 24 | 24 | Social-Emotional Z-Score_lowerhb | 110 | Random | 531 | 310 | -0.30 (-0.54, -0.06) | 0.51 | 0.013 |
| 24 | 24 | Motor Z-Score | 107 | Fixed | 440 | 642 | -0.08 (-0.19, 0.03) | 0.00 | 0.165 |
| 24 | 24 | Motor Z-Score | 107 | Random | 440 | 642 | -0.08 (-0.19, 0.03) | 0.00 | 0.165 |
| 24 | 24 | Motor Z-Score | 108 | Fixed | 469 | 613 | -0.10 (-0.22, 0.01) | 0.00 | 0.078 |
| 24 | 24 | Motor Z-Score | 108 | Random | 469 | 613 | -0.10 (-0.22, 0.01) | 0.00 | 0.078 |
| 24 | 24 | Motor Z-Score | 110 | Fixed | 530 | 552 | -0.16 (-0.27, -0.04) | 0.00 | 0.008 |
| 24 | 24 | Motor Z-Score | 110 | Random | 530 | 552 | -0.16 (-0.27, -0.04) | 0.00 | 0.008 |
| 27 | 27 | Gross Motor Z-Score | 110 | Fixed | 276 | 595 | -0.06 (-0.21, 0.09) | 0.00 | 0.431 |
| 27 | 27 | Gross Motor Z-Score | 110 | Random | 276 | 595 | -0.06 (-0.21, 0.09) | 0.00 | 0.431 |
| 27 | 27 | Gross Motor Z-Score | 115 | Fixed | 436 | 435 | -0.11 (-0.25, 0.04) | 0.00 | 0.147 |
| 27 | 27 | Gross Motor Z-Score | 115 | Random | 436 | 435 | -0.11 (-0.25, 0.04) | 0.00 | 0.147 |
| 27 | 27 | Gross Motor Z-Score | 116 | Fixed | 464 | 407 | -0.11 (-0.25, 0.04) | 0.00 | 0.145 |
| 27 | 27 | Gross Motor Z-Score | 116 | Random | 464 | 407 | -0.11 (-0.25, 0.04) | 0.00 | 0.145 |
| 6 | 12 | Walking alone | 109 | Fixed | 2119 | 1863 | -0.05 (-0.08, -0.02) | 0.03 | 0.002 |
| 6 | 12 | Walking alone | 109 | Random | 2119 | 1863 | -0.05 (-0.08, -0.02) | 0.03 | 0.002 |
| 6 | 12 | Walking alone | 110 | Fixed | 2258 | 1724 | -0.04 (-0.07, -0.01) | 0.00 | 0.008 |
| 6 | 12 | Walking alone | 110 | Random | 2258 | 1724 | -0.04 (-0.07, -0.01) | 0.00 | 0.008 |
| 6 | 12 | walkalone_lowerhb | 103 | Fixed | 1418 | 1526 | -0.07 (-0.10, -0.03) | 0.37 | 0.000 |
| 6 | 12 | walkalone_lowerhb | 103 | Random | 1418 | 1526 | -0.08 (-0.12, -0.03) | 0.37 | 0.003 |
| 6 | 12 | walkalone_lowerhb | 110 | Fixed | 2258 | 686 | -0.07 (-0.11, -0.02) | 0.57 | 0.002 |
| 6 | 12 | walkalone_lowerhb | 110 | Random | 2258 | 686 | -0.08 (-0.14, -0.01) | 0.57 | 0.021 |
| 6 | 18 | Gross Motor Z-Score | 108 | Fixed | 1847 | 1774 | -0.06 (-0.13, 0.01) | 0.00 | 0.077 |
| 6 | 18 | Gross Motor Z-Score | 108 | Random | 1847 | 1774 | -0.06 (-0.13, 0.01) | 0.00 | 0.077 |
| 6 | 18 | Gross Motor Z-Score | 110 | Fixed | 2064 | 1557 | -0.05 (-0.12, 0.02) | 0.00 | 0.133 |
| 6 | 18 | Gross Motor Z-Score | 110 | Random | 2064 | 1557 | -0.05 (-0.12, 0.02) | 0.00 | 0.133 |
| 6 | 18 | Fine Motor Z-Score | 106 | Fixed | 1683 | 2018 | 0.00 (-0.07, 0.06) | 0.00 | 0.967 |
| 6 | 18 | Fine Motor Z-Score | 106 | Random | 1683 | 2018 | 0.00 (-0.07, 0.06) | 0.00 | 0.967 |
| 6 | 18 | Fine Motor Z-Score | 110 | Fixed | 2115 | 1586 | 0.03 (-0.04, 0.09) | 0.00 | 0.438 |
| 6 | 18 | Fine Motor Z-Score | 110 | Random | 2115 | 1586 | 0.03 (-0.04, 0.09) | 0.00 | 0.438 |
| 6 | 18 | Nap Index Z-Score | 104 | Fixed | 433 | 1025 | -0.03 (-0.10, 0.04) | 0.37 | 0.440 |
| 6 | 18 | Nap Index Z-Score | 104 | Random | 433 | 1025 | -0.03 (-0.12, 0.06) | 0.37 | 0.475 |
| 6 | 18 | Nap Index Z-Score | 107 | Fixed | 550 | 908 | -0.01 (-0.08, 0.05) | 0.00 | 0.658 |
| 6 | 18 | Nap Index Z-Score | 107 | Random | 550 | 908 | -0.01 (-0.08, 0.05) | 0.00 | 0.658 |
| 6 | 18 | Nap Index Z-Score | 110 | Fixed | 690 | 768 | -0.01 (-0.07, 0.05) | 0.00 | 0.734 |
| 6 | 18 | Nap Index Z-Score | 110 | Random | 690 | 768 | -0.01 (-0.07, 0.05) | 0.00 | 0.734 |
| 6 | 24 | Social-Emotional Z-Score | 106 | Fixed | 631 | 898 | -0.05 (-0.16, 0.06) | 0.00 | 0.371 |
| 6 | 24 | Social-Emotional Z-Score | 106 | Random | 631 | 898 | -0.05 (-0.16, 0.06) | 0.00 | 0.371 |
| 6 | 24 | Social-Emotional Z-Score | 107 | Fixed | 676 | 853 | -0.05 (-0.16, 0.05) | 0.00 | 0.333 |
| 6 | 24 | Social-Emotional Z-Score | 107 | Random | 676 | 853 | -0.05 (-0.16, 0.05) | 0.00 | 0.333 |
| 6 | 24 | Social-Emotional Z-Score | 109 | Fixed | 776 | 753 | -0.03 (-0.12, 0.07) | 0.00 | 0.561 |
| 6 | 24 | Social-Emotional Z-Score | 109 | Random | 776 | 753 | -0.03 (-0.12, 0.07) | 0.00 | 0.561 |
| 6 | 24 | Social-Emotional Z-Score | 110 | Fixed | 834 | 695 | -0.01 (-0.11, 0.08) | 0.00 | 0.769 |
| 6 | 24 | Social-Emotional Z-Score | 110 | Random | 834 | 695 | -0.01 (-0.11, 0.08) | 0.00 | 0.769 |
| 12 | 24 | Language Z-Score | 110 | Fixed | 370 | 485 | -0.11 (-0.26, 0.03) | 0.00 | 0.130 |
| 12 | 24 | Language Z-Score | 110 | Random | 370 | 485 | -0.11 (-0.26, 0.03) | 0.00 | 0.130 |
| 12 | 24 | Language Z-Score | 111 | Fixed | 393 | 462 | -0.12 (-0.25, 0.02) | 0.00 | 0.095 |
| 12 | 24 | Language Z-Score | 111 | Random | 393 | 462 | -0.12 (-0.25, 0.02) | 0.00 | 0.095 |
| 12 | 24 | Social-Emotional Z-Score | 109 | Fixed | 352 | 513 | -0.16 (-0.31, 0.00) | 0.66 | 0.044 |
| 12 | 24 | Social-Emotional Z-Score | 109 | Random | 352 | 513 | -0.10 (-0.41, 0.21) | 0.66 | 0.539 |
| 12 | 24 | Social-Emotional Z-Score | 110 | Fixed | 373 | 492 | -0.16 (-0.30, -0.01) | 0.75 | 0.033 |
| 12 | 24 | Social-Emotional Z-Score | 110 | Random | 373 | 492 | -0.07 (-0.44, 0.31) | 0.75 | 0.731 |
| 18 | 24 | Language Z-Score | 110 | Fixed | 792 | 2047 | -0.12 (-0.21, -0.03) | 0.00 | 0.007 |
| 18 | 24 | Language Z-Score | 110 | Random | 792 | 2047 | -0.12 (-0.21, -0.03) | 0.00 | 0.007 |
| 18 | 24 | Language Z-Score | 116 | Fixed | 1358 | 1481 | -0.10 (-0.17, -0.03) | 0.00 | 0.008 |
| 18 | 24 | Language Z-Score | 116 | Random | 1358 | 1481 | -0.10 (-0.17, -0.03) | 0.00 | 0.008 |
| 18 | 24 | Language Z-Score_lowerhb | 110 | Fixed | 792 | 1636 | -0.15 (-0.24, -0.06) | 0.00 | 0.001 |
| 18 | 24 | Language Z-Score_lowerhb | 110 | Random | 792 | 1636 | -0.15 (-0.24, -0.06) | 0.00 | 0.001 |
| 18 | 24 | Language Z-Score_lowerhb | 113 | Fixed | 1050 | 1378 | -0.18 (-0.26, -0.10) | 0.00 | 0.000 |
| 18 | 24 | Language Z-Score_lowerhb | 113 | Random | 1050 | 1378 | -0.18 (-0.26, -0.10) | 0.00 | 0.000 |
| 18 | 24 | Language Z-Score_lowerhb | 116 | Fixed | 1358 | 1070 | -0.14 (-0.22, -0.06) | 0.00 | 0.000 |
| 18 | 24 | Language Z-Score_lowerhb | 116 | Random | 1358 | 1070 | -0.14 (-0.22, -0.06) | 0.00 | 0.000 |
| 18 | 24 | Language Z-Score_upperhb | 125 | Fixed | 516 | 642 | 0.05 (-0.06, 0.17) | 0.00 | 0.358 |
| 18 | 24 | Language Z-Score_upperhb | 125 | Random | 516 | 642 | 0.05 (-0.06, 0.17) | 0.00 | 0.358 |
| 18 | 24 | Motor Z-Score | 110 | Fixed | 784 | 2040 | -0.09 (-0.17, -0.02) | 0.00 | 0.014 |
| 18 | 24 | Motor Z-Score | 110 | Random | 784 | 2040 | -0.09 (-0.17, -0.02) | 0.00 | 0.014 |
| 18 | 24 | Motor Z-Score | 116 | Fixed | 1349 | 1475 | -0.08 (-0.14, -0.01) | 0.00 | 0.017 |
| 18 | 24 | Motor Z-Score | 116 | Random | 1349 | 1475 | -0.08 (-0.14, -0.01) | 0.00 | 0.017 |
| 18 | 24 | Motor Z-Score | 117 | Fixed | 1467 | 1357 | -0.08 (-0.14, -0.01) | 0.00 | 0.017 |
| 18 | 24 | Motor Z-Score | 117 | Random | 1467 | 1357 | -0.08 (-0.14, -0.01) | 0.00 | 0.017 |
| 18 | 24 | Gross Motor Z-Score | 110 | Fixed | 784 | 2045 | -0.08 (-0.15, 0.00) | 0.00 | 0.022 |
| 18 | 24 | Gross Motor Z-Score | 110 | Random | 784 | 2045 | -0.08 (-0.15, 0.00) | 0.00 | 0.022 |
| 18 | 24 | Gross Motor Z-Score | 116 | Fixed | 1351 | 1478 | -0.06 (-0.13, 0.00) | 0.00 | 0.053 |
| 18 | 24 | Gross Motor Z-Score | 116 | Random | 1351 | 1478 | -0.06 (-0.13, 0.00) | 0.00 | 0.053 |
| 18 | 24 | Gross Motor Z-Score | 117 | Fixed | 1469 | 1360 | -0.07 (-0.13, 0.00) | 0.00 | 0.042 |
| 18 | 24 | Gross Motor Z-Score | 117 | Random | 1469 | 1360 | -0.07 (-0.13, 0.00) | 0.00 | 0.042 |
| 21 | 24 | Language Z-Score | 110 | Fixed | 127 | 282 | -0.16 (-0.34, 0.03) | 0.00 | 0.054 |
| 21 | 24 | Language Z-Score | 110 | Random | 127 | 282 | -0.16 (-0.37, 0.05) | 0.00 | 0.054 |
| 21 | 24 | Language Z-Score | 116 | Fixed | 200 | 209 | -0.24 (-0.41, -0.07) | 0.11 | 0.005 |
| 21 | 24 | Language Z-Score | 116 | Random | 200 | 209 | -0.25 (-0.43, -0.07) | 0.11 | 0.007 |
| 21 | 24 | Language Z-Score | 117 | Fixed | 223 | 186 | -0.21 (-0.37, -0.04) | 0.64 | 0.013 |
| 21 | 24 | Language Z-Score | 117 | Random | 223 | 186 | -0.22 (-0.51, 0.08) | 0.64 | 0.152 |

**Supplemental Figure 1**. Hb distributions by trial


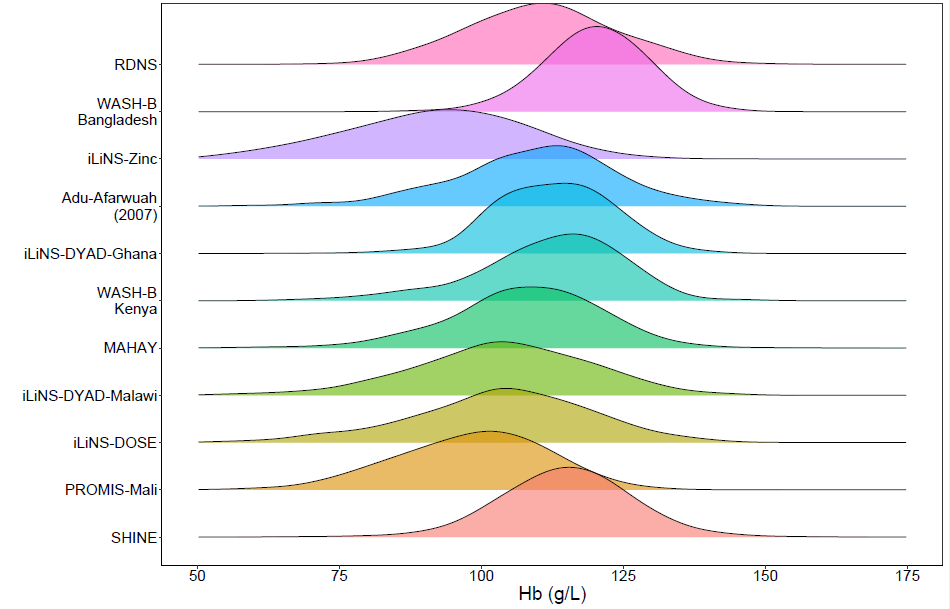

Supplement: online supplemental file 1 [file bmjgh-11-4-s001.docx]
